# Antiapoptotic Protein FAIM2 is targeted by miR-3202, and DUX4 via TRIM21, leading to cell death and defective myogenesis

**DOI:** 10.1038/s41419-022-04804-x

**Published:** 2022-04-25

**Authors:** Hossam A. N. Soliman, Erik A. Toso, Inas E. Darwish, Samia M. Ali, Michael Kyba

**Affiliations:** 1 Lillehei Heart Institute, Minneapolis, USA; 2 Department of Pediatrics, University of Minnesota, Minneapolis, MN 55455 USA; 3 Alexandria University, Faculty of Medicine, Department of Clinical Pharmacology, Alexandria, Egypt

**Keywords:** Apoptosis, Antagomir and RNA sponge, miRNAs, Neuromuscular disease, Muscle stem cells

## Abstract

Inappropriate expression of DUX4, a transcription factor that induces cell death at high levels of expression and impairs myoblast differentiation at low levels of expression, leads to the development of facioscapulohumeral muscular dystrophy (FSHD), however, the pathological mechanisms downstream of DUX4 responsible for muscle loss are poorly defined. We performed a screen of 1972 miR inhibitors for their ability to interfere with DUX4-induced cell death of human immortalized myoblasts. The most potent hit identified by the screen, miR-3202, is known to target the antiapoptotic protein FAIM2. Inhibition of miR-3202 led to the upregulation of FAIM2, and remarkably, expression of DUX4 led to reduced cellular levels of FAIM2. We show that the E3 ubiquitin ligase and DUX4 target gene, TRIM21, is responsible for FAIM2 degradation downstream of DUX4. Human myoblasts overexpressing FAIM2 showed increased resistance to DUX4-induced cell death, whereas in wild-type cells FAIM2 knockdown resulted in increased apoptosis and failure to differentiate into myotubes. The necessity of FAIM2 for myogenic differentiation of WT cells led us to test the effect of FAIM2 overexpression on the impairment of myogenesis by DUX4. Strikingly, FAIM2 overexpression rescued the myogenic differentiation defect caused by low-level expression of DUX4. These data implicate FAIM2 levels, modulated by DUX4 through TRIM21, as an important factor mediating the pathogenicity of DUX4, both in terms of cell viability and myogenic differentiation, and thereby open a new avenue of investigation towards drug targets in FSHD.

## Graphical abstract

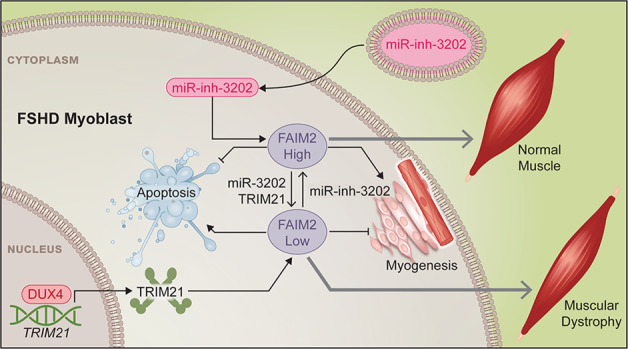

## Introduction

Facioscapulohumeral muscular dystrophy (FSHD) is an autosomal dominant inherited myopathic disorder. Most patients experience a slow and steady disease course as muscles of the face, shoulder girdles, and upper arms atrophy gradually [1]. Although FSHD is one of the most common myopathic disorders [2], the pathological mechanism is still not fully understood, and there is no specific treatment. The disease is caused by mutations that perturb the silencing of a repeated gene, referred to as DUX4 [3]. These typically occur in *cis*, by reducing the copy number of the repeat [4], but can also occur in *trans*, by disrupting proteins involved in silencing of the repeat [5–7]. DUX4 is a transcription factor with hundreds of targets [8, 9], and overexpression studies have shown that it causes cell death when expressed at high levels [8, 10] and interferes with myogenic differentiation when expressed at low levels [8, 11]. Both effects would be deleterious to muscle maintenance, and their relative contributions to FSHD is a topic of current interest. The inhibition of differentiation by DUX4 seems primarily mediated by interference with expression of myogenic regulatory factors [8, 11, 12]. Various pathways have been proposed to be involved in DUX4-mediated cell death, including that of the histone acetyltransferase co-activator p300, which is required by DUX4 to activate its targets [13, 14], the induction of double-stranded RNA [15], activation of p53 [16], however, see Bosnakovski et al. [17], impaired protein degradation and TDP-43 accumulation [18], and hyaluronic acid signaling [19].

The micro RNA (miR) network has not been specifically investigated in the context of DUX4 downstream effects. DUX4 promotes apoptosis, and several miRs are known regulators of both the intrinsic and extrinsic pathways of apoptosis, including miRs targeting *BCL-XL*, *MCL1*, and *EGFR* [20–22]. Other miRs are involved in the external pathway of apoptosis through targeting *TRAIL* and *FASL* [23–26]. Some of these miRs can directly induce apoptosis, while others protect cell viability by preventing it. Because little is known about the intracellular mechanism by which DUX4 induces apoptosis, we wished to perform an unbiased screen of miR inhibitors, covering as much of the known human miR map as possible, with the hope of identifying miRs regulating components of the apoptotic machinery or upstream pathways that impinge on apoptosis in response to DUX4, and perhaps thereby identifying key apoptotic players downstream of DUX4. To do this, we employed a cell-based assay quantifying DUX4-induced cell death [27, 28] in the presence of miR inhibitors. Our approach had the potential to discover both DUX4-induced toxic miRs and miRs targeting factors that antagonize DUX4, and revealed the importance of miR-3202 and its target, FAIM2, in DUX4-induced cytotoxicity.

## Results

### Assay design and development

**Fig. 1:**
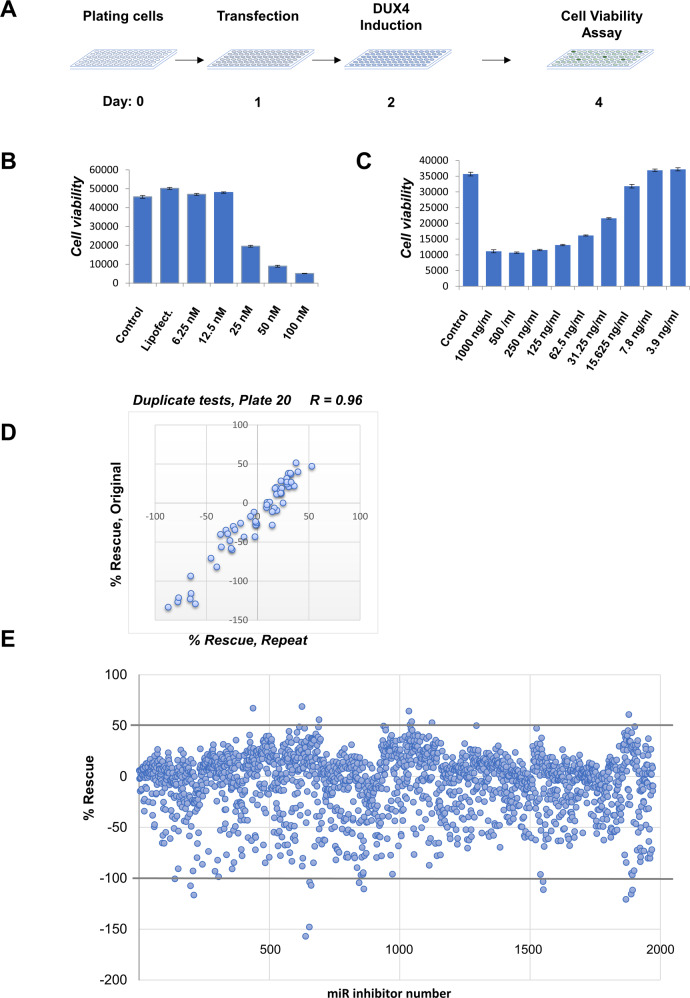
miR inhibitor screen in the LHCN-M2iDUX4 cell disease model. **A** Timeline of the screen indicating plating of the cells at day 0, transfection of individual miRNA inhibitors into individual wells at day 1, then induction of DUX4 expression by 50 ng/ml doxycycline for 48 h followed by cell viability assay. **B** Optimization of the miRNA inhibitors concentration using the control toxic inhibitor. **C** Optimization of doxycycline induction dose. **D** Repeat of a single library plate showing correlation of normalized percentage of rescue scores. **E** Screening results for the 1972 Micro RNA inhibitor library. Normalized viability of cells exposed to 50 ng/mL dox and 50 nM micro RNA inhibitor is indicated on the *y*-axis.

To identify miRs with a potential role in cell death mediated by DUX4 in an unbiased way, we elected to screen the miRCURY library of miR inhibitors targeting 78% of known human miRs (miRbase v20). The inhibitors are oligonucleotides incorporating a locked nucleic acid backbone modification with perfect sequence complementarity to the miRs targeted. We screened miR inhibitors in an immortalized human myoblast cell line with a doxycycline (dox)-inducible DUX4 transgene, referred to as LHCN-M2iDUX4 [13] (Fig. 1A). To determine the optimal concentration of miR inhibitors, we performed a dose-response experiment using a known toxic miR inhibitor (Fig. 1B), which demonstrated full effect at 50 nM; thus we used this concentration for the screen. In order to be able to identify both DUX4-antagonistic and DUX4-augmenting miRs, we induced DUX4 with 50 ng/mL of dox, which gives an intermediate level of cell death, based on a dox dose-response curve (Fig. 1C). LHCN-M2iDUX4 cells in 96-well dishes were transfected with miR inhibitors, dox was provided 24 h later, and after an additional 48 h of continuous DUX4 induction, cells were tested for viability using an indirect ATP-content assay (Fig. 1A). Cell number was optimized in order for cells to be growing exponentially for the duration of the screen, as DUX4 toxicity is greater in proliferating cells [27], and to avoid overgrowth.

### Identification of miR inhibitors that inhibit or augment DUX4-mediated cell death

We assigned to each inhibitor a percent rescue score, normalizing each inhibitor to the two controls on its plate, uninduced and dox-induced LHCN-M2iDUX4 cells. To assess reproducibility, two individual plates were repeated independently at different time points. Results from the independent assays showed excellent correlations (Fig. 1D, S1A).

We then completed the primary screen of 1972 miR inhibitors, using the same internal plate normalization method (Fig. 1E, and see Table S1 for results from the entire library). The screen yielded a number of protective inhibitors whose transfection significantly increased cell viability following DUX4 induction (Table 1). In addition, the screen also identified a number of miR inhibitors whose transfection resulted in a marked increase in DUX4 toxicity (Table 2). The miRs that this latter set targets presumably play a protective role, and notably the screen identified miR-675, recently identified as targeting the DUX4 transcript itself [29]. Of interest, we noted that inhibitors of a large number of previously annotated myomiRs, i.e., miRs showing enriched or exclusive expression in muscle, and/or promoting myogenic differentiation [30], showed enhancement of DUX4 toxicity (Table S2). As DUX4 is less toxic to post-mitotic cells than to proliferating myoblasts [8, 27], inhibition of these myomiRs might enhance cell death by keeping cells in a more proliferative, less differentiated, state.

**Table 1:** List of the top protective miR inhibitors providing >50% rescue of the DUX4-induced myoblast death.

miRBase ID	miRBase accession	microRNA target sequence	% rescue
hsa-miR-3202	MIMAT0015089	UGGAAGGGAGAAGAGCUUUAAU	68.8
hsa-miR-384	MIMAT0001075	AUUCCUAGAAAUUGUUCAUA	67.1
hsa-miR-5091	MIMAT0021083	ACGGAGACGACAAGACUGUGCUG	64.4
hsa-miR-4432	MIMAT0018948	AAAGACUCUGCAAGAUGCCU	60.9
hsa-miR-2115-3p	MIMAT0011159	CAUCAGAAUUCAUGGAGGCUAG	55.9
hsa-miR-3614-3p	MIMAT0017993	UAGCCUUCAGAUCUUGGUGUUUU	54.1
hsa-miR-4767	MIMAT0019919	CGCGGGCGCUCCUGGCCGCCGCC	52.9
hsa-miR-3648	MIMAT0018068	AGCCGCGGGGAUCGCCGAGGG	50.8
hsa-miR-4634	MIMAT0019691	CGGCGCGACCGGCCCGGGG	50.1

**Table 2:** List of the top toxic miR inhibitors.

miRBase ID	miRBase accession	microRNA target sequence	% rescue
hsa-miR-1260a	MIMAT0005911	AUCCCACCUCUGCCACCA	−157
hsa-let-7a-2-3p	MIMAT0010195	CUGUACAGCCUCCUAGCUUUCC	−147.9
hsa-miR-3127-3p	MIMAT0019201	UCCCCUUCUGCAGGCCUGCUGG	−120.7
hsa-miR-766-3p	MIMAT0003888	ACUCCAGCCCCACAGCCUCAGC	−116.5
hsa-miR-4659b-3p	MIMAT0019734	UUUCUUCUUAGACAUGGCAGCU	−115.6
hsa-miR-4701-5p	MIMAT0019798	UUGGCCACCACACCUACCCCUU	−111.6
hsa-miR-4713-5p	MIMAT0019820	UUCUCCCACUACCAGGCUCCCA	−111.2
hsa-miR-1260b	MIMAT0015041	AUCCCACCACUGCCACCAU	−110.4
hsa-miR-605-5p	MIMAT0003273	UAAAUCCCAUGGUGCCUUCUCCU	−107.3
hsa-miR-218-1-3p	MIMAT0004565	AUGGUUCCGUCAAGCACCAUGG	−106.9
hsa-miR-584-3p	MIMAT0022708	UCAGUUCCAGGCCAACCAGGCU	−104.3
hsa-miR-621	MIMAT0003290	GGCUAGCAACAGCGCUUACCU	−103.8
hsa-miR-3161	MIMAT0015035	CUGAUAAGAACAGAGGCCCAGAU	−103.3
hsa-miR-197-3p	MIMAT0000227	UUCACCACCUUCUCCACCCAGC	−100.5
hsa-miR-675-3p*	MIMAT0006790	CUGUAUGCCCUCACCGCUCA	−62.7

* Recently identified as targeting DUX4 [41].

Of greater interest, the screen yielded a number of protective inhibitors whose transfection significantly increased cell viability following DUX4 induction (Table 1). Three of these have been directly linked to apoptosis: miR-4767 in vascular epithelial cells through targeting BCL2L12 and EGFR [31], miR-3202 which was shown to cause apoptosis in endothelial cells [32], and miR-384 has been linked to inducing apoptosis in certain types of cancers [33, 34].

### miR-3202 inhibition protects against DUX4-induced myoblast death

**Fig. 2:**
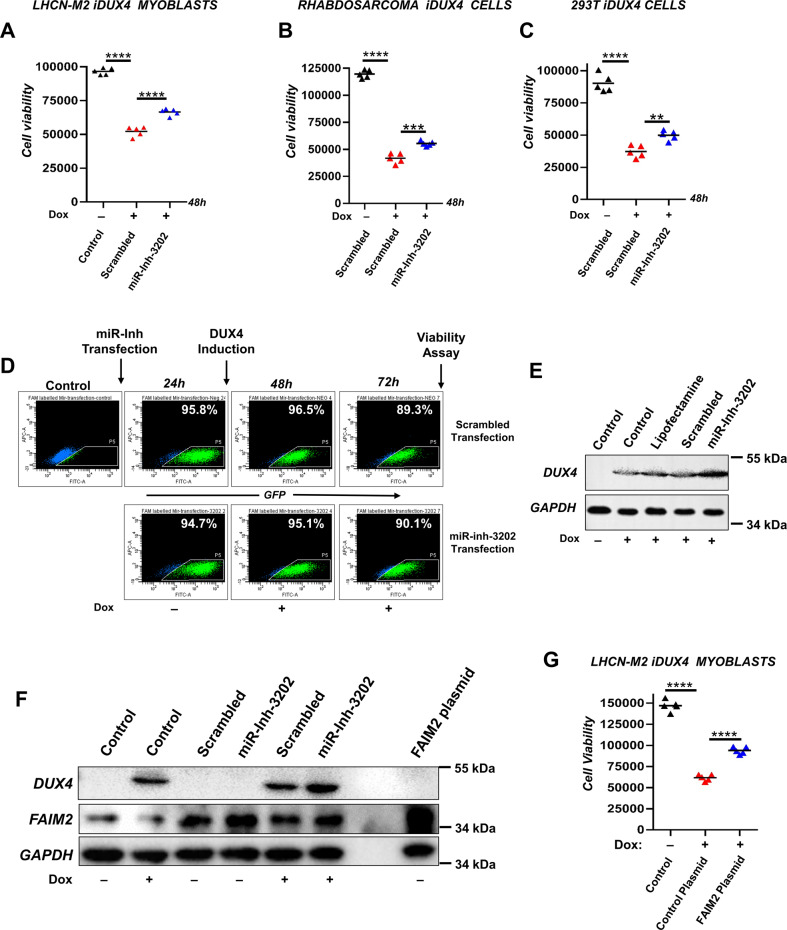
Inhibiting miR-3202 protects against DUX4-induced cell death by upregulating FAIM2. **A** Cell viability indicated by CellTitre-Glo luminescence, measuring total ATP content in control non-transfected, non-induced, and scramble- or miR-3202 inhibitor-transfected doxycycline-induced LHCN-M2iDUX4 cells. ****P < 0.0001 by one-way ANOVA with Tukey’s post hoc test (*n* = 5). The experiment has been repeated five independent times. **B** Cell viability of iDUX4 rhabdosarcoma cells, transfected and induced as in **A**. ****P < 0.0001, ***P = 0.0002 by one-way ANOVA with Tukey’s post hoc test (*n* = 5). **C** Cell viability of 293TiDUX4 cells, transfected and induced as in **A**. Data are presented as individual data points plus mean; ****P < 0.0001, **P = 0.0065 by one-way ANOVA with Tukey’s post hoc test (*n* = 5) the experiment has been repeated 3 independent times. **D** FACS analysis of FAM-labeled miR-3202 inhibitor transfection efficiency across the duration of the assay, showing over 90% transfection efficiency in LHCN-M2iDUX4 cells. **E** Western blot for DUX4 expression in LHCN-M2iDUX4 cells transfected (or not) with miR-3202 inhibitor for 24 h and induced (or not) with 50 ng/ml doxycycline for another 24 h. **F** Western blot for DUX4 and FAIM2 expression in LHCN-M2iDUX4 cells transfected with miR-3202 inhibitor or controls, with or without subsequent induction of DUX4 by 50 ng/ml doxycycline. Far-right lane shows LHCN-M2iDUX4 cells transfected with FAIM2-expressing plasmid. **G** Cell viability Assay of LHCN-M2iDUX4 cells induced with 50 ng/ml for 48 h following transfection with FAIM2 expression plasmid versus a control non-coding plasmid. ****P < 0.0001, by one-way ANOVA with Tukey’s post hoc test (*n* = 5).

We repurchased independently the most potent inhibitor, that of miR-3202, tested for reproducibility, and found that it again decreased DUX4-induced myoblast death (Fig. 2A). To ensure that its activity was generally involved in cell death driven by DUX4, and not specific to a cell type, we tested inhibiting miR-3202 in an additional dox-inducible-DUX4 myogenic cell type, a rhabdomyosarcoma line, and in a non-myogenic cell type, 293 T cells [11]. Inhibition of miR-3202 reduced the toxicity of DUX4 in both cell types (Fig. 2B, C). Transfection of FITC-conjugated versions of the inhibitor of miR-3202 and the scrambled control showed that >90% of cells incorporate inhibitor 24 h post-transfection, and that it is maintained through to the 72-h time point, corresponding to the time point at which we tested viability (Fig. 2D). To exclude the trivial explanation that the inducible system was impaired by transfection of miR-3202 inhibitor, we probed western blots for DUX4 expression, which showed that DUX4 was not reduced, and was in fact slightly increased in the presence of the inhibitor (Fig. 2E). We attribute this effect to the inhibitor enhancing viability of the cells, allowing them to produce more DUX4.

### miR-3202 inhibition protects against DUX4-induced myoblast death by upregulating FAIM2

miR-3202 has been described in previous studies to target *Fas apoptosis inhibitory molecule 2*, *FAIM2* [32, 35], also known as *Lifeguard* [36]. We, therefore, investigated FAIM2 levels and found an increase in FAIM2 protein following miR-3202 inhibition (Fig. 2F). In addition, direct transfection of FAIM2-expressing plasmid showed that FAIM2 significantly increased the viability of the human myoblasts following DUX4 induction (Fig. 2G). These data suggest that miR-3202 inhibition is protective against DUX4 through upregulation of the antiapoptotic protein FAIM2. Surprisingly, we also noted that FAIM2 protein levels decreased following DUX4 induction (Fig. 2F), suggesting that reduction in antiapoptotic activity of FAIM2 might be part of the mechanism of DUX4 toxicity. This directed us to further studies investigating the relationship between DUX4 and FAIM2.

### FAIM2 overexpression improves survival of myoblasts after DUX4 induction

**Fig. 3:**
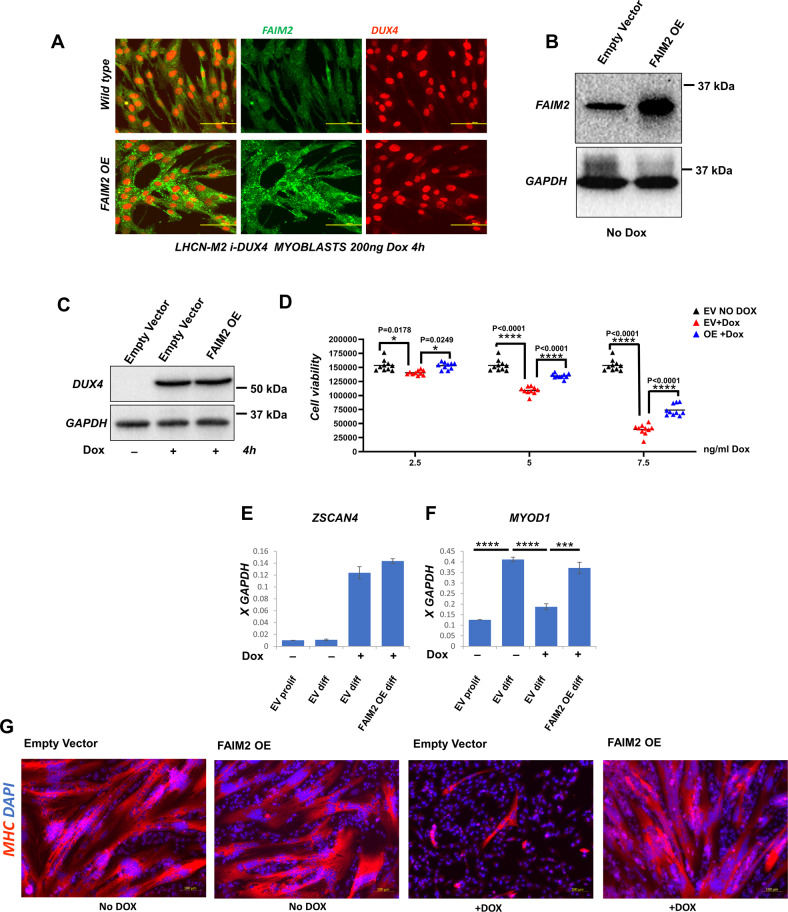
FAIM2 overexpression protects against DUX4-induced myoblast pathology. **A** Immunofluorescent staining for FAIM2 (green) and DUX4 (red) in wild-type LHCN-M2iDUX4 cells (top) and LHCN-M2iDUX4 cells with FAIM2 overexpression (OE) (bottom) showing cytoplasmic FAIM2 protein in green and nuclear DUX4 protein in red following induction by 200 ng/ml doxycycline for 4 h. **B** Western blot for FAIM2 in LHCN-M2iDUX4 cells transduced with either FAIM2 overexpression (OE) lentivirus or empty vector. **C** Western blot for DUX4 expression in LHCN-M2iDUX4 cells carrying either FAIM2 overexpression or empty vector control, induced by 200 ng/ml doxycycline for 4 h. **D** Viability of LHCN-M2iDUX4 cells in the presence of FAIM2 overexpression or empty vector (EV) after 1 week of DUX4 induction by 2.5, 5, and 7.5 ng/ml doxycycline. Data are presented as individual data points plus mean; statistical analysis employed two-way ANOVA with Tukey’s post hoc test (*n* = 10), *p*-values are indicated above the comparisons. **E** RTqPCR for *ZSCAN4* showing equal expression in both the empty vector (EV) and the overexpression (OE) cell line after 48 h in differentiation medium preceded by 48 h in the presence or absence of DUX4 induction in proliferation medium. **F** RTqPCR for *MYOD1* levels in both the empty vector and the overexpression (OE) cell line in the same samples in **E**. Data are presented as means ± SEM; ****P < 0.0001, ***P = 0.0002 by one-way ANOVA with Tukey’s post hoc test (*n* = 3). **G** Immunofluorescent staining for sarcomeric myosin heavy chain (MHC) (red) and DAPI (blue) of LHCN-M2iDUX4 myoblasts in the presence of FAIM2 overexpression or the empty vector after 2 days in proliferation medium followed by 2 days in differentiation medium, all with or without DUX4 induction (DOX).

We modified LHCN-M2iDUX4 cells using a constitutive FAIM2-expressing lentivector. In the derived cells, FAIM2 could be visualized in clear excess in the overexpression cell line (Fig. 3A, B). DUX4 was equally inducible after 4 h of doxycycline induction in the empty vector control and the FAIM2 overexpression lines (Fig. 3A, C). The stable cell line allowed us to study the effects of longer-term, very low, levels of DUX4 expression, physiologically closer to what is observed in FSHD, where DUX4 is extremely difficult to detect in patient muscle biopsies. We thus treated cells with a low dose series of doxycycline (2.5, 5, and 7.5 ng/mL) and measured viability after one week of continuous DUX4 induction (Fig. 3D). This revealed that FAIM2 overexpression gave DUX4-expressing cells a survival advantage, with the degree of advantage correlating with the levels of DUX4 expression, but reaching significance at all doses.

As it had not previously been shown that FAIM2 regulated apoptosis in myogenic cells, we also tested the simple effect of overexpressing FAIM2 in LHCN-M2 cells exposed to other apoptotic signals. FAIM2 overexpression protected cells against apoptosis induced by oxidative stress (Supplemental Fig. S2A) and, to a lesser extent, BCL-2 inhibition (Supplemental Fig. S2B). Thus, FAIM2 is a bona fide inhibitor of apoptosis in myogenic cells, whose activity is negatively regulated by both DUX4 and miR-3202.

### FAIM2 overexpression counters DUX4 effects on myogenic differentiation

As mentioned, DUX4 has two pathological effects: cell death and inhibition of myogenic differentiation. To test whether FAIM2 has any effect on DUX4-induced inhibition of myogenesis, we subjected both the overexpression line and the empty vector line to DUX4 induction by doxycycline for 48 h followed by myogenic differentiation for another 48 h. At the end of the 96h-duration of the experiment, we tested for *ZSCAN4* and *MYOD1*. *ZSCAN4*, a DUX4 target gene [9], was equally stimulated in both cell lines (Fig. 3E), indicating that FAIM2 overexpression does not interfere with the inducible system or the transcriptional activity of DUX4. We then evaluated *MYOD1*, due to its central role in myogenesis [37] and the fact that DUX4 rapidly downregulates it [8, 11]. In the absence of DUX4 induction, the control empty vector cells upregulate *MYOD1* in response to the differentiation (Fig. 3F). On the other hand, significant inhibition of *MYOD1* expression was observed in the DUX4-induced empty vector cells (Fig. 3F). Remarkably, almost normal upregulation of MYOD1 was observed in the FAIM2 overexpression line following differentiation (Fig. 3F).

We then evaluated terminal differentiation by immunostaining for sarcomeric myosin following 2 days of proliferation and 2 days of differentiation in the continuous presence or absence of DUX4. In the absence of DUX4, both the empty vector and the overexpression lines differentiated normally into myosin heavy chain positive myotubes (Fig. G). In the presence of DUX4 induction, the empty vector line failed to form myotubes as expected, while the FAIM2 overexpression line differentiated robustly (Fig. G). These data indicate that FAIM2 can counteract both pathological effects of DUX4: cell death, and inhibition of differentiation.

### FAIM2 is necessary for viability and differentiation potential of wild-type myoblasts

**Fig. 4:**
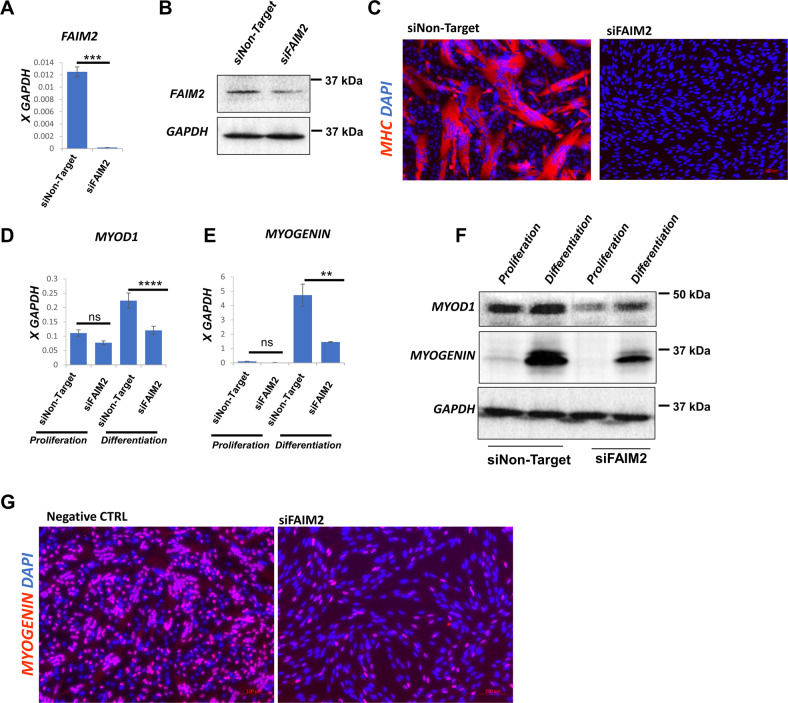
siRNA knockdown of FAIM2 blocks myogenic differentiation. **A** RTqPCR for *FAIM2* expression in LHCN-M2 myoblasts transfected with non-targeting siRNA (siNon-Target) or siRNA targeting FAIM2 (siFAIM2) for 72 h. Data are presented as mean ± SEM; ***P = 0.0001, by unpaired *t*-test (*n* = 3). **B** Western blot for FAIM2 expression in LHCN-M2 myoblasts transfected with non-targeting siRNA (siNon-Target) or siRNA targeting *FAIM2* (siFAIM2) for 72 h. **C** Immunofluorescent staining for MHC (red) and DAPI (blue) of LHCN-M2 myoblasts transfected with non-targeting siRNA (left) or siRNA targeting *FAIM2* (right) for 72 h, then cultured for 24 h in differentiation medium. **D** RTqPCR for *MYOD1* expression in LHCN-M2 myoblasts transfected with non-targeting siRNA or siRNA targeting *FAIM2* mRNA for 72 h and then differentiated for 24 h. Data are presented as mean ± SEM; for ns *P* = 0.1463, ****P < 0.0001 by two-way ANOVA with Tukey’s post hoc test (*n* = 3). **E** RTqPCR for *MYOGENIN* expression in the same cells shown in **D**. Data are presented as means ± SEM; for ns *P* = 0.9988, **P = 0.0014 by two-way ANOVA with Tukey’s post hoc test (*n* = 3). **F** Western blot for MYOD1 and MYOGENIN for the same cells shown in **D**. **G** Immunofluorescent staining of MYOGENIN (red) and DAPI (blue) of LHCN-M2iDUX4 myoblasts after transfection with 50 nM *FAIM2* siRNA or Non-Target siRNA for 72 h followed by 24 h in differentiation medium.

To investigate the normal function of FAIM2 in healthy myoblasts, we performed siRNA knockdown of FAIM2 in unmodified LHCN-M2 cells. 72 h after transfection, both RNA and protein levels of FAIM2 were effectively reduced (Fig. 4A, B). Upon change to differentiation medium, FAIM2 knockdown cells showed a complete failure of differentiation in comparison with the control non-target siRNA (Fig. 4C). FAIM2 knockdown reduced both MYOD1 and MYOGENIN protein levels in the proliferating state, and prevented their upregulation at both the RNA and protein levels in the differentiating state (Fig. 4D–G). These are the first data to demonstrate a necessity for FAIM2 in myogenesis.

**Fig. 5:**
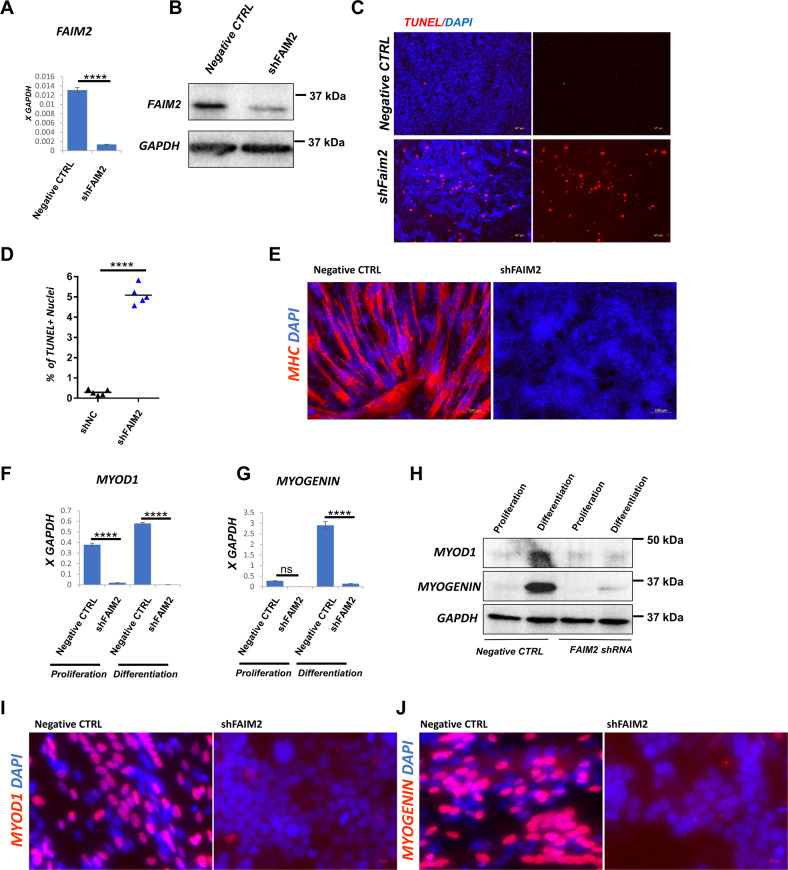
shRNA knockdown of FAIM2 promotes myoblast apoptosis and blocks myogenic differentiation. **A** RTqPCR for *FAIM2* expression in LHCN-M2 myoblasts transduced with a lentivector expressing shRNA targeting FAIM2 or empty vector control. Data are presented as mean ± SEM; ****P < 0.0001, by unpaired *t*-test (*n* = 3). **B** Western blot analysis of *FAIM2* expression in shRNA knockdown and control LHCN-M2 myoblasts, as in A. **C** Immunofluorescent staining of TUNEL positive cells (red) and DAPI (blue) in shRNA knockdown (bottom) or control (top) LHCN-M2 myoblasts. **D** Frequency of TUNEL positive LHCN-M2 cells in shRNA knockdown or control LHCN-M2 myoblasts. Data were taken from Immunofluorescence images of five different wells of each cell line. Data are presented as individual data points plus mean; ****P < 0.0001, by unpaired *t*-test (*n* = 5). **E** Immunofluorescent staining for MHC (red) and DAPI (blue) in FAIM2 knockdown (right) or control (left) LHCN-M2 myoblasts, differentiated for 48 h. **F** RTqPCR for *MYOD1* expression in *FAIM2* knockdown and control LHCN-M2 myoblasts during proliferation or after 48 h of differentiation. Data are presented as means ± SEM; ****P < 0.0001 by two-way ANOVA with Tukey’s post hoc test (*n* = 3). **G** RTqPCR for *MYOGENIN* expression in the same cells as in **H**. Data are presented as means ± SEM; for ns *P* = 0.1686, ****P < 0.0001 by two-way ANOVA with Tukey’s post hoc test (*n* = 3). **H** Western blot analysis of *MYOD1* and *MYOGENIN* expression in *FAIM2* knockdown or control LHCN-M2 myoblasts during proliferation or after 24 h of differentiation. **I** Immunofluorescent staining of MYOD1 (red) and DAPI (blue) in *FAIM2* knockdown or control LHCN-M2 myoblasts, differentiated for 48 h. **J** Immunofluorescent staining of MYOGENIN (red) and DAPI (blue) in *FAIM2* knockdown or control LHCN-M2 myoblasts, differentiated for 48 h.

To better evaluate long-term effects of FAIM2 inhibition, we generated a stable FAIM2 knockdown LHCN-M2 cell line using an shRNA lentivector targeting FAIM2, which reduced both RNA and protein levels of FAIM2 (Fig. 5A, B). Shortly after the line was established, we observed a noticeable increase in cell death in the FAIM2 shRNA line compared to the non-targeting shRNA control line. This was confirmed by TUNEL assay (Fig. 5C, D). As with siRNA-treated myoblasts, shRNA-expressing myoblasts showed severely impaired differentiation (Fig. 5E), in this case with a more significant reduction in MYOD1 and MYOGENIN levels, and a failure to upregulate both with differentiation (Fig. 5F–J).

### The E3 ubiquitin ligase TRIM21 downregulates FAIM2 downstream of DUX4

**Fig. 6:**
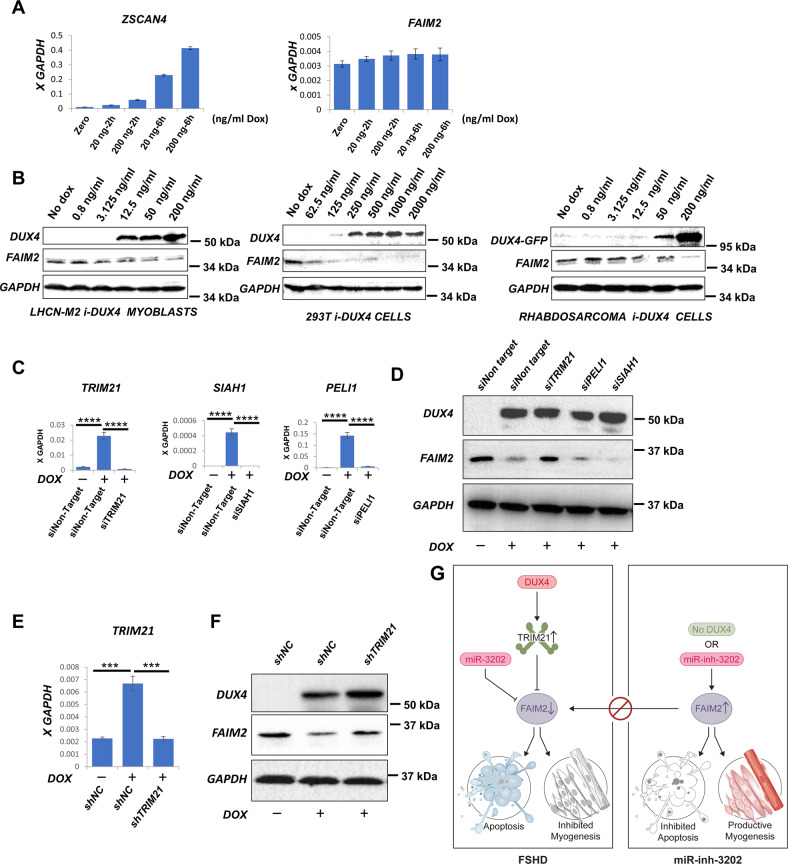
DUX4 downregulates FAIM2 by upregulating E3 ubiquitin ligase TRIM21. **A** RTqPCR for *ZSCAN4* and *FAIM2* expression showing an increase in DUX4 target gene *ZSCAN4* accompanying DUX4 induction by doxycycline, which is not associated with any significant change in *FAIM2* expression levels. **B** Western blot for FAIM2 and DUX4 expression in three independent cell lines following a dose series induction of DUX4 expression showing dose-dependent reduction of FAIM2 expression in each. **C** RTqPCR for *TRIM21*, *SIAH1,* and *PELI1* following transfection of LHCN-M2iDUX4 cells with the corresponding siRNA for each gene or control non-targeting siRNA (siNon-Target). Data are presented as means ± SEM; ****P < 0.0001, by one-way ANOVA with Tukey’s post hoc test (*n* = 3). **D** Western blot for FAIM2 and DUX4 in LHCN-M2iDUX4 cells transfected with non-targeting siRNA (siNon-Target) or siRNAs targeting *TRIM21*, *PELI1,* or *SIAH1* for 24 h followed by induction of DUX4 expression with 200 ng/ml doxycycline for another 24 h. The western blot was repeated twice from two independent transfections. **E** RTqPCR for *TRIM21* expression in LHCN-M2iDUX4 cells transduced with lentivector expressing shRNA targeting *TRIM21* or negative control non-silencing shRNA (shNC), with or without induction of DUX4 with 200 ng/ml doxycycline for 24 h. Data are presented as means ± SEM; in both cases, ***P = 0.0003 by one-way ANOVA with Tukey’s post hoc test (*n* = 3). **F** Western blot for FAIM2 and DUX4 in the same cells shown in **E**. The western blot was repeated twice. **G** Schematic diagram showing pathways influencing FAIM2 levels, including DUX4 via TRIM21 and miR-3202, or its inhibitor, and the consequences of FAIM2 levels on myogenic differentiation and cell viability.

The involvement of FAIM2 in myoblast viability and differentiation heightens the significance of its downregulation by DUX4. This decline in protein abundance was not accomplished through a reduction in *FAIM2* transcription as FAIM2 RNA levels were not decreased, even as the DUX4 target gene *ZSCAN4* was upregulated in a dose-dependent manner (Fig. 6A). To determine the generality of DUX4 reducing FAIM2 protein levels, we evaluated FAIM2 in the additional human cell lines engineered with the inducible DUX4 transgene described above. In all 3 cell lines, immortalized myoblasts, rhabdomyosarcoma cells, and 293 T epithelial cells, we observed a DUX4 dose-responsive reduction in FAIM2 protein, shortly after induction (Fig. 6B).

To understand the potential post-translational effect of DUX4 on FAIM2, we noted that E3 ubiquitin ligases are a prominent subset of DUX4 target genes [9]. We evaluated three E3 ubiquitin ligases that are significantly upregulated by DUX4 in LHCN-M2 cells [13], SIAH1, PELI1, and TRIM21. Of note, FAIM2 has been previously linked to TRIM21 in a breast cancer study [38]. We confirmed the upregulation of all 3 genes in LHCN-M2iDUX4 cells in response to dox, as well as their knockdown by transfection with specific siRNAs (Fig. 6C). Western blot analysis of FAIM2 protein levels showed that FAIM2 levels decreased in response to DUX4 expression in control and with PELI1 or SIAH1 knockdowns, but not with TRIM21 knockdown (Fig. 6D). To extend these results, we also knocked down TRIM21 using shRNA. The shRNA knockdown was less efficient than the siRNA knockdown, but reduced TRIM21 levels in the presence of DUX4 to levels normally seen in the absence of DUX4 (Fig. 6E). Under these conditions, we again observed attenuation of the decrease in FAIM2 levels by DUX4 (Fig. 6F). These data indicate that the DUX4 target gene and E3 ubiquitin ligase TRIM21 is necessary for the reduction in FAIM2 levels upon DUX4 expression (Fig. 6G).

## Discussion

The pro-apoptotic effects of DUX4 when overexpressed at high levels are well known [8, 10], however, the apoptotic regulatory network downstream of DUX4 has not been deeply investigated to date. In particular, the role that miRNAs may play has been understudied, a situation made more significant by the fact that many miR mimics and antagonists have already reached phase I and II clinical trials [39]. The inhibitor screen we present has identified both miRs that antagonize DUX4 activity (increase DUX4 toxicity when inhibited), as well as miRs that enhance or are required for DUX4 activity (increase viability when inhibited).

With regard to the first category of miRs identified in our screen, those that antagonize DUX4, we noted that a significant number of these are specifically associated with skeletal muscle, or expressed during myogenic differentiation, and have been referred to as myomiRs [40]. These include miR-1, miR-133a and b, miR-208b, miR-499a and b, and miR-675. Inhibiting these led to increased toxicity of DUX4 in human myoblasts. Conversely, it is notable that one of these, miR-675, has been shown to antagonize DUX4 toxicity when overexpressed and has been proposed as a potential therapeutic in FSHD [41].

Among the second category, miRs that contribute to DUX4 toxicity, our top hit, miR-3202 targeted the antiapoptotic protein, FAIM2. As FAIM2 had not been investigated in muscle to date, we studied the effect of knocking it down and overexpressing it in wild-type human myoblasts. The knockdown led to increased apoptosis, and the overexpression protected cells from death stimuli (oxidative stress and BCL-2 inhibition), confirming its role in protecting myogenic cells from death-inducing signals. More remarkably, however, we found that FAIM2 was necessary for differentiation of myoblasts into myotubes. Thus, FAIM2 is necessary for the very processes that DUX4 inhibits: cell viability and myogenic differentiation. In accordance with this, overexpression of FAIM2 partially protects cells from DUX4-induced apoptosis and fully restores the ability of DUX4-expressing myoblasts to differentiate.

FAIM2 was originally discovered to antagonize Fas-mediated cell death [36], and has been identified as a protective factor in a number of diseases [42–46] as well as a factor promoting tumor growth and aggressiveness of various cancers [47–50]. While it is possible that DUX4 engages the death receptor pathway, Fas ligand is not among the genes upregulated by DUX4 [13] so another apoptosis entry point is likely, and indeed FAIM2 is reported to interact with multiple pro- and anti-apoptotic proteins [51, 52], and has documented Fas-independent activities [50]. Interestingly, maintaining FAIM2 expression when very low levels of DUX4 were induced led to a survival and selective advantage of FAIM2-expressing cells. This is important because it is not yet settled in the field whether DUX4 acts through sporadic high-level expression, or extremely low-level expression. Although excellent monoclonal antibodies to DUX4 exist, to date, no study has shown direct evidence of DUX4 expression in muscle tissue sections by immunostaining, favoring the latter theory. The ability of FAIM2 to inhibit phenotypes associated with very low levels of DUX4 expression supports the physiological relevance of the relationships demonstrated in this study.

TRIM21 is a ubiquitous E3 ubiquitin ligase expressed in most tissues [53]. It is an antiviral factor that detects intracellular viruses ligated by antibodies prior to infection or during cell internalization, by binding the Fc domain and moving internalized viral particles to the proteasome for degradation [53]. The efficient antibody-mediated protein degrading activity of TRIM21 has enabled a protein knockout method known as TRIM-away, in which electroporation of antibodies leads to depletion of the selected protein target within minutes [54]. TRIM21 also has Fc-independent activity, targeting certain ubiquitinated proteins for proteasomal degradation, for example, TRIM21 targets the SQSTM1 protein, also known as P62 [55]. In this context, the knockdown of TRIM21 was associated with an antioxidant response and the ability of cells to better withstand oxidative stress through enhanced p62 activity.

In the DUX4 context, we found that TRIM21 is the DUX4 target responsible for FAIM2 downregulation. DUX4 upregulates TRIM21 which in turn causes reduction in FAIM2 protein levels. As RNA levels of FAIM2 are not reduced after DUX4 induction, TRIM21-mediated inhibition of FAIM2 is post-translational, likely through FAIM2 ubiquitination and subsequent proteasomal degradation. This was supported by TRIM21 knockdown, which rescued FAIM2 protein levels from being downregulated by DUX4. Future studies are warranted to explore the relationship between FAIM2 protein levels and TRIM21 induction by DUX4; however our data confirms the necessity of TRIM21 in the mechanism of DUX4-induced FAIM2 reduction, making TRIM21 a candidate therapeutic target for FSHD together with FAIM2 itself.

In summary, our screen has identified a miR inhibitor that counteracts DUX4 cytotoxicity, and has identified a new member of the apoptosis regulatory system, FAIM2, which antagonizes DUX4 toxicity, both in terms of its ability to promote cell death and to block differentiation. We find also that FAIM2 is necessary for differentiation of normal human myoblasts into myotubes. We show that the DUX4 target gene and E3 ubiquitin ligase TRIM21 is necessary for DUX4-mediated downregulation of FAIM2, making the DUX4-TRIM21-FAIM2 axis a potentially attractive target for drug discovery and development in FSHD.

## Methods

### miR inhibitor library

Exiqon 1972 miRCURY human LNA (locked nucleic acid) miR inhibitor library was purchased from Qiagen, Germantown, MD. miR inhibitors were screened at 50 nM.

### Cell Culture

LHCN-M2 iDUX4 cells were cultured in Ham’s F10 medium (SH30025.01, Hyclone, Logan, UT) supplemented with 20% fetal bovine serum (FBS) (PS-FB3, PeakSerum, Wellington CO), 1% glutamax (35050079, Gibco, Gaithersburg, MD), penicillin and streptomycin (CX30324, Gibco Gaithersburg, MD), 10 ng/ml hb-FGF (PeproTech, Cranbury, NJ) and 100 nM dexamethasone (11015, Cayman Chemical Company, Ann Arbor, MI). 293 T cells and rhabdosarcoma cells were cultured in DMEM-HG medium supplemented with 10% FBS (PS-FB3, PeakSerum, Wellington CO), 1% glutamax (Gibco), 1% penicillin and streptomycin (Gibco). Cultures were maintained at 37 °C in a 5% CO_2_ atmosphere.

### Transfection

RNAiMAX transfection reagent (Invitrogen by Thermo Fisher, Waltham, MA) was used for transfecting miR inhibitors and siRNAs. For secondary screening, miR-3202 inhibitor was used at 100 nM. Repurchased miR inhibitors were obtained from Qiagen, Germantown, MD: YI04105161-ACA (miR-3202) and YI00199006-ACA (negative control). siRNA for *FAIM2* (Dharmacon, Lafayette, CO, L-017340-00-0005), *TRIM21* (Dharmacon, L-006563-00-0005), *SIAH1* (Dharmacon, L-012598-01-0005), *PELI1* (Dharmacon, L-013814-01-0005) were used at a concentration of 50 nM. For differentiation assays, cells were incubated with *FAIM2* siRNA for 72 h followed by differentiation for 24 h. Cells were incubated with *TRIM21*, *SIAH1* and *PELI1* siRNA for 24 h followed by 24 h induction with 200 ng/ml doxycycline. FAIM2 expression plasmid was purchased from GenScript, Piscataway, NJ, (NM_012306.4 CloneID OHu20849) and was transfected using TransIT®-LT1 Transfection Reagent, Mirus, Madison, WI.

### Viability assays

A single subclone of LHCN-M2 iDUX4 cells was expanded to make a frozen stock of cells at the same proliferative stage to be used for the entire screen. For viability assays, cells were plated by FACS into 96-well plates. CellTiter-Glo Luminescent Cell Viability Assay (Promega, Madison, WI) was used in the analysis. Medium was replaced with CellTitre-Glo reagent then read on a Cytation3 plate reader (BioTek, Winooski, VT). Viability data from the primary screen was normalized to uninduced cells, representing maximum possible viability and dox-induced cells treated with a negative control (scramble) LNA, representing DUX4-induced viability loss. Percent rescue was calculated as (inhibitor+dox - scramble+dox)/(no dox - scramble+dox).

### Western blot analysis

Antibodies used included rabbit anti-DUX4 1:50 (MAB95351, R&D Systems, Minneapolis, MN), mouse anti-DUX4 1:500 (9A12, gift of A. Belayew), rabbit anti-DUX4 1:1000 (ab124699, Abcam, Cambridge MA), rabbit anti-FAIM2 1 µgm/ml (AS-54488, Anaspec, Fremont, CA), rabbit anti-FAIM2 1:500 (LS-C404955-200, LifeSpan Biosciences, Seattle, WA), rabbit anti-FAIM2 1:500 (TA306043, OriGene, Rockville, MD), Mouse Anti-MyoD (554130, BDbiosciences, Franklin Lakes, NJ, USA), Mouse anti-Myogenin (556358, BDbiosciences, Franklin Lakes, NJ, USA), HRP-conjugated anti-GAPDH 1:5000 (HRP-600004, Proteintech Rosemont, IL), secondary HRP-conjugated anti-rabbit 1:10,000 (111-035-003, Jackson ImmunoResearch, West Grove, PA), secondary HRP-conjugated anti-mouse 1:5,000 (NB1-75130, Novusbio, Centennial, CO).

### RTqPCR

RNA was extracted using Quick-RNA MiniPrep kit (Zymo Research, Irvine, CA) according the manufacturer’s instructions. cDNA was synthesized using the high-capacity cDNA reverse transcription kit (Thermo Fisher, Waltham, MA). qPCR was performed with Premix Ex Taq Master Mix (Takara, Mountain View, CA). TaqMan probes (Thermo Fisher) were used for the assay including *GAPDH*: Hs99999905_m1; *ZSCAN4*: Hs00537549_m1, *FAIM2*: HS00392342_m1, *MYOD1*: Hs00159528_m1, *MYOG*: Hs01072232_m1 *TRIM21*: Hs00172616_m1, *SIAH1*: Hs02339360_m1, *PELI1*: Hs00900505_m1 (Applied Biosystems, Beverly, MA).

### Generation of FAIM2 vector-expressing lentivirus plasmids

The cDNA for *FAIM2* was obtained from GenScript (Piscataway, NJ), amplified using primers: EcoRI-FAIM2 forward (5'- GCT TGA TAT CGA ATT CCC ATG ACC CAG GGA AAG CTC TCC G -3'), BAMH1-FAIM2 reverse (5'- TAG AAC TAG TGG ATC CTC ATT CTC GGT TAG TGC CAA AAA GC -3'), and subcloned into the pSAM-CAGGS-ires-mCherry lentiviral vector by In-Fusion HD Cloning (Takara).

### Lentivector production and generation of FAIM2/control-expressing LHCN-M2 iDUX4 cells

Viral supernatants were produced in 293 T cells by transfecting with Mirus LTI transfection reagent (Mirus Bio) together with pVSVG and ∆8.9 packaging constructs. Medium was changed after 24 h and the viral supernatants collected 48 h post-transfection. Cells were incubated with 0.45 µm-filtered supernatant supplemented with 10 µg/mL polybrene (Millipore Sigma, St. Louis, MO) overnight at 37 °C, after which the supernatant was replaced with fresh medium. Several days after infection, LHCN-M2 iDUX4 cells were FACS sorted for mCherry expression on a BD FACSAria (BDbiosciences, Franklin Lakes, NJ).

### Generation of shRNA knockdown lines

For *FAIM2* and *TRIM21* knock downs, pGIPZ V2LHS_51135 and pGIPZ V2LHS_153528 resp. lentiviral clones were purchased from University of Minnesota Genomics Centre together with a negative control nonsilencing lentiviral vector. Lentivirus production and LHCN-M2 iDUX4 myoblast transduction was done in the same way as for the FAIM2 overexpression.

### Immunofluorescence

Cells were fixed with 4% paraformaldehyde for 20 min, permeabilized by 0.3% Triton X for 30 min, and blocked by 3% BSA (bovine serum albumin) for 1 h at room temperature. Primary antibodies included mouse monoclonal anti-DUX4 9A12, a gift of A. Belayew (1:500), 10 µg/ml rabbit polyclonal anti-FAIM2 (AS-54488, Anaspec, Fermont, CA), mouse antiMHC (MF 20 S, Developmental Studies Hybridoma Bank, Iowa City, IA) mouse Anti-MyoD (554130, BDbiosciences, Franklin Lakes, NJ, USA), mouse anti-Myogenin (556358, BDbiosciences, Franklin Lakes, NJ, USA) diluted in 3% BSA in PBS at 4 °C overnight.

### FACS analysis

Transfection efficiency was tested by FACS. The FAM label on the miR-3202 inhibitor was detected in the FITC channel. FAIM2/Empty Vector overexpressing cell lines were established by sorting on endogenous expression of mCherry. All samples were analyzed on BD FACSAria.

### TUNEL assay

Cells were fixed with 4% PFA in PBS for 20 min at room temperature, blocked, and permeabilized using 0.3% triton in 3% BSA in PBS for 1 h at room temperature. Procedure was performed using In Situ Cell Death Detection Kit, TMR red (12156792910, Sigma-Aldrich, St. Louis, MO).

### Statistics

Statistical analysis was performed using Prism (GraphPad, San Diego, CA). Sample size was selected in a balance of cost of experiments and increasing statistical confidence.

## Supplementary information

Supplemental western blots uncropped

Supplemental Material Figures

Supplemental Table S1

Supplemental Table S2

Checklist

## Data Availability

The data that support the findings of this study are available from the corresponding author upon reasonable request.
