# Supplementary material for: Antiapoptotic Protein FAIM2 is targeted by miR-3202, and DUX4 via TRIM21, leading to cell death and defective myogenesis
Source: Cell Death Dis. 2022 Apr 25;13(4):405. doi: 10.1038/s41419-022-04804-x (PMC9038730; doi:10.1038/s41419-022-04804-x)
Supplement: Supplementary file 1 — Supplemental western blots uncropped [file 41419_2022_4804_MOESM1_ESM.pdf]

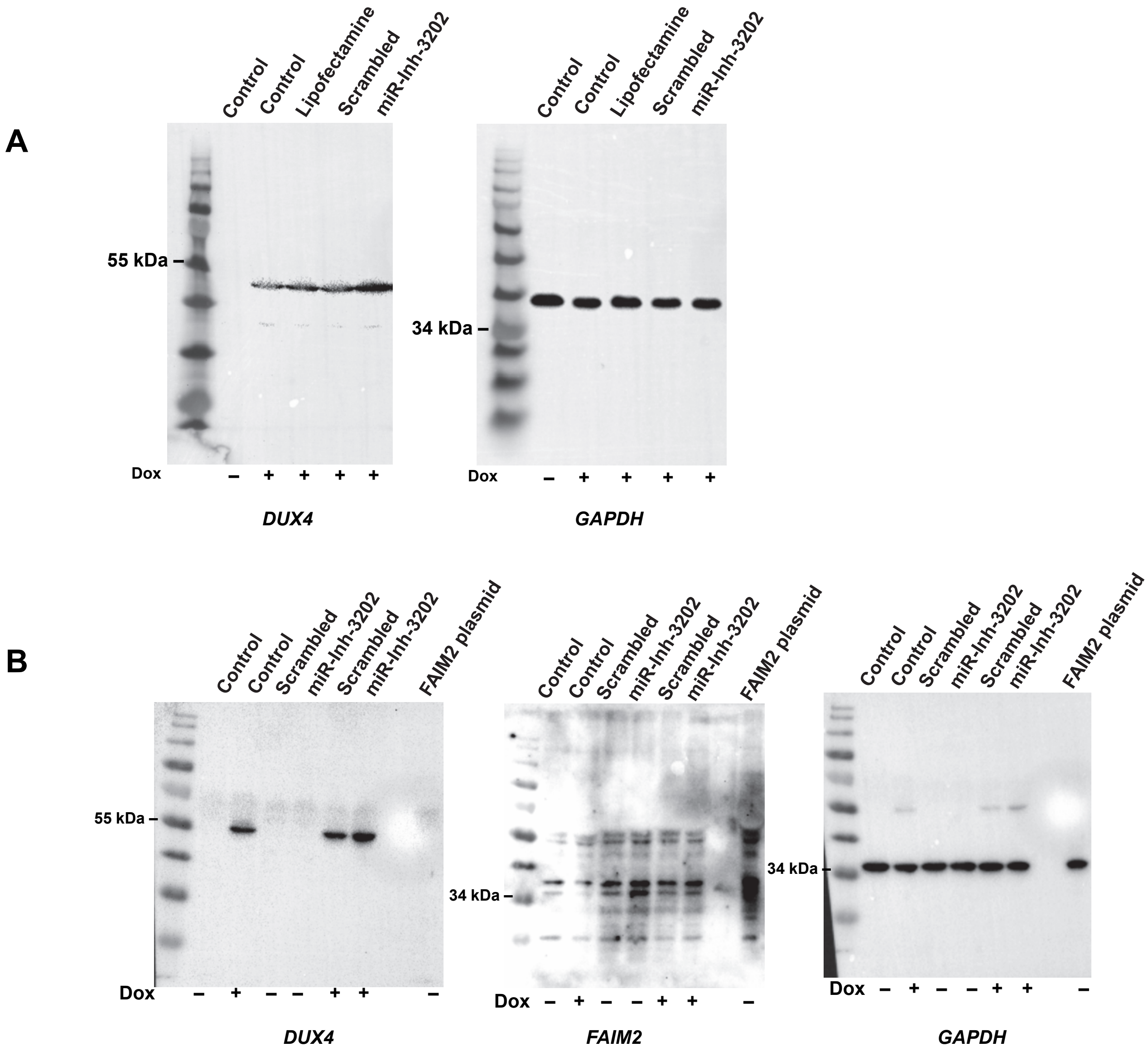

Uncropped western blots from Figure 2 of the main article.
A) Western blots of Fig. 2E
B) Western blots of Fig. 2F

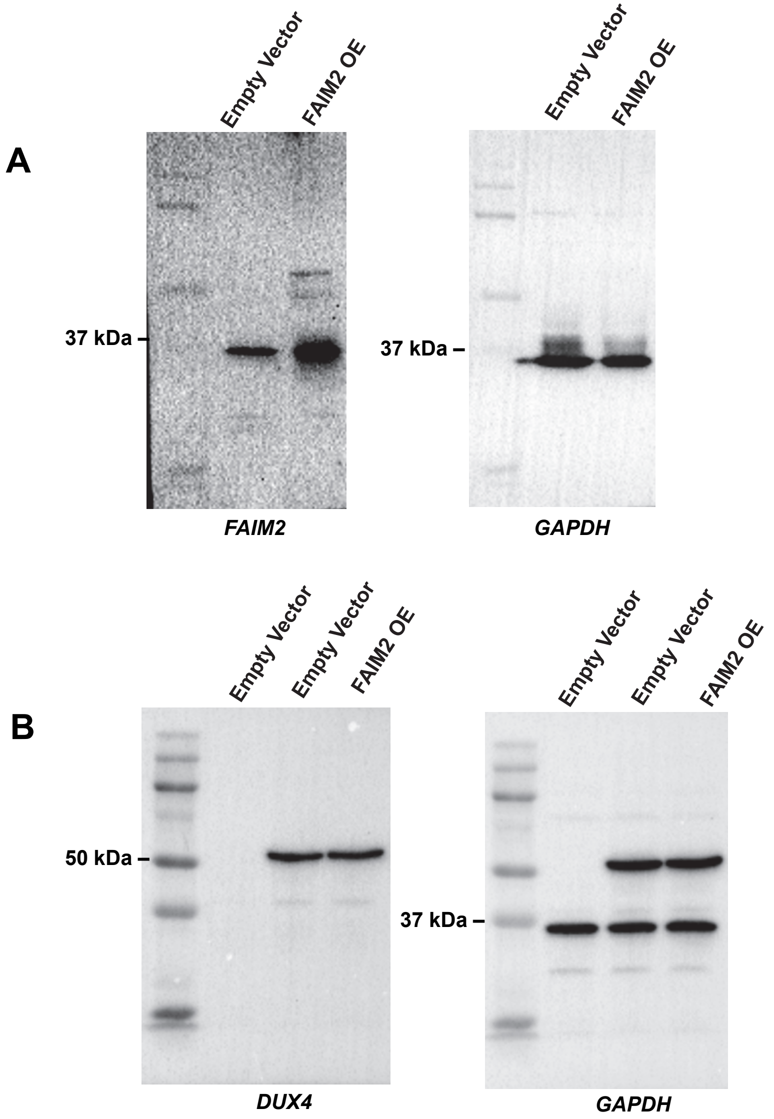

Uncropped western blots from Figure 3 of the main article.

A) Western blots of Fig. 3B

B) Western blots of Fig. 3C

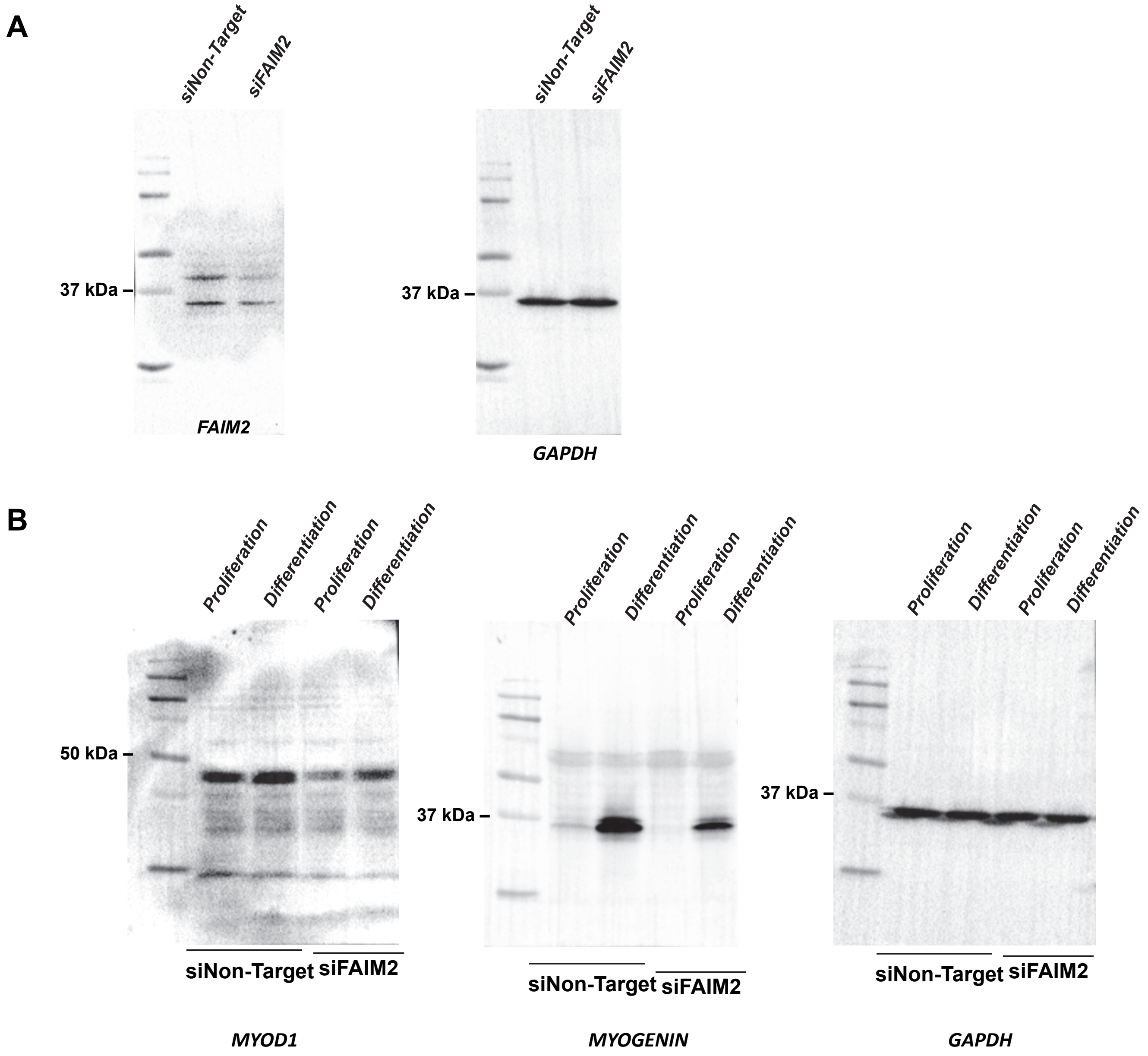

Uncropped western blots from Figure 4 of the main article

A) Western blots of Fig. 4B
B) Western blots of Fig. 4F

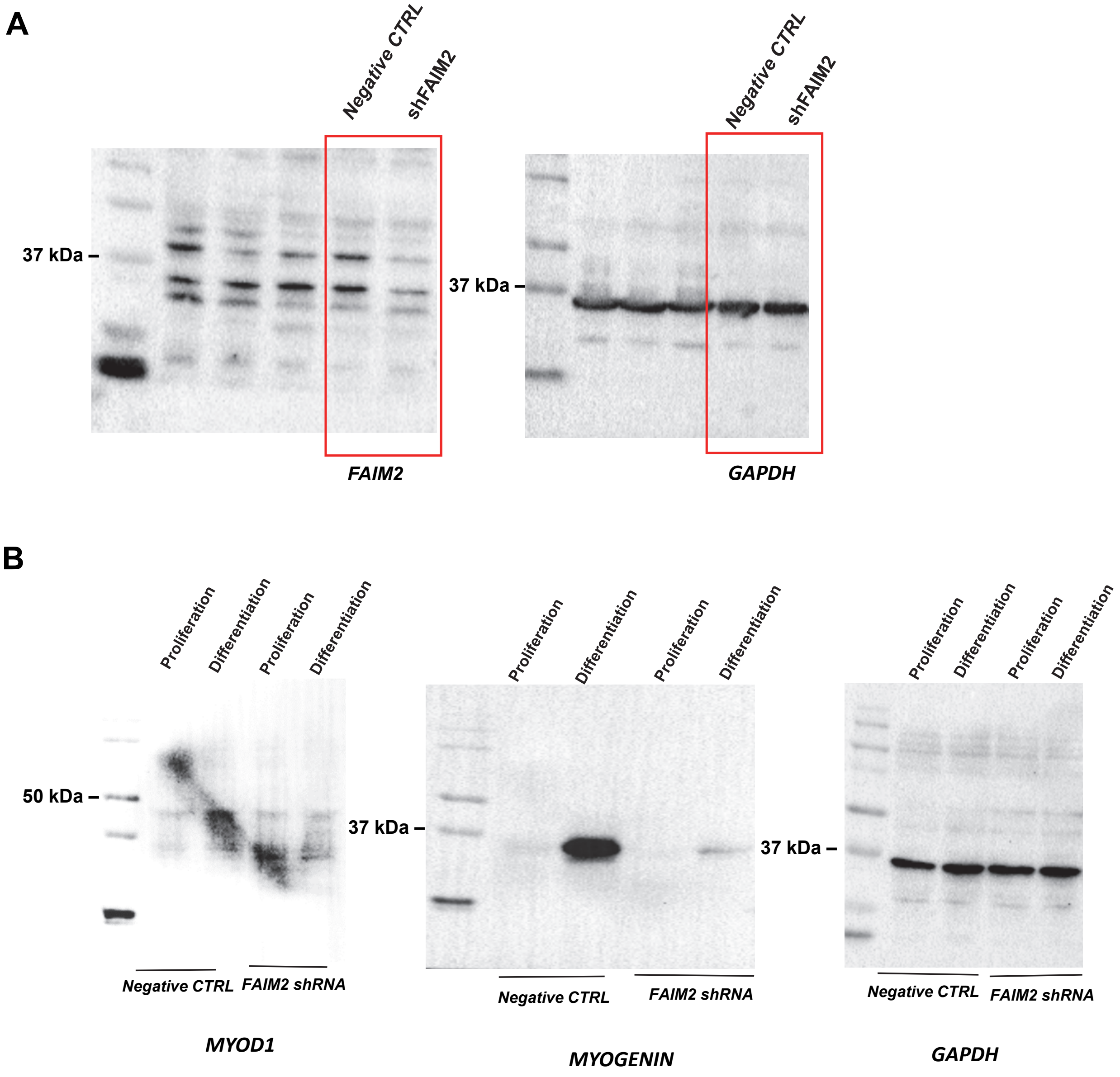

Uncropped western blots from Figure 5 of the main article

A) Western blots of Fig. 5B (relevant lanes highlighted in red)

B) Western blots of Fig. 5H

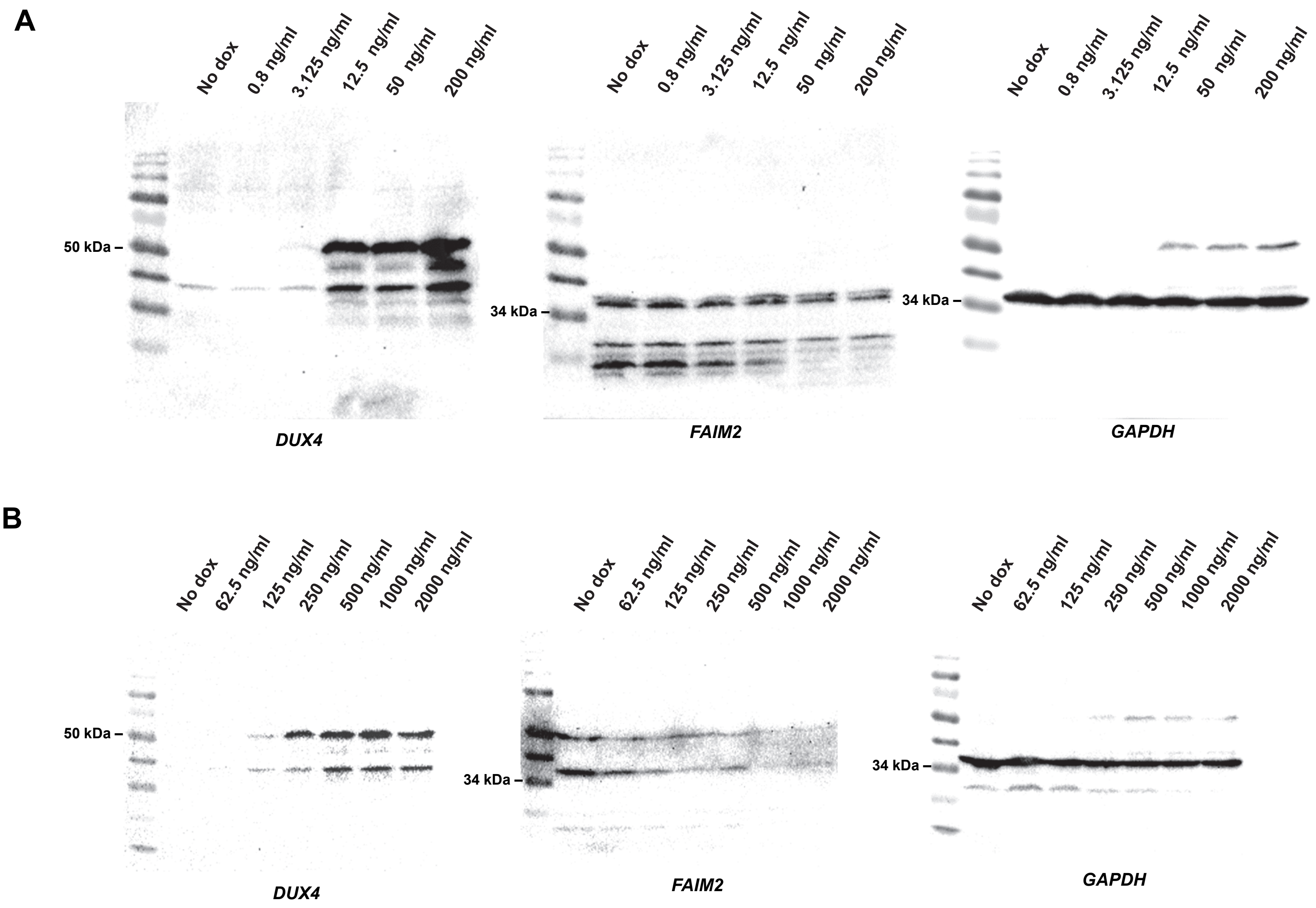

Uncropped western blots from Figure 6 of the main article

A) Western blots of Fig. 6B of LHCN-M2 i-DUX4 myoblasts

B) Western blots of Fig. 6B of 293T cells

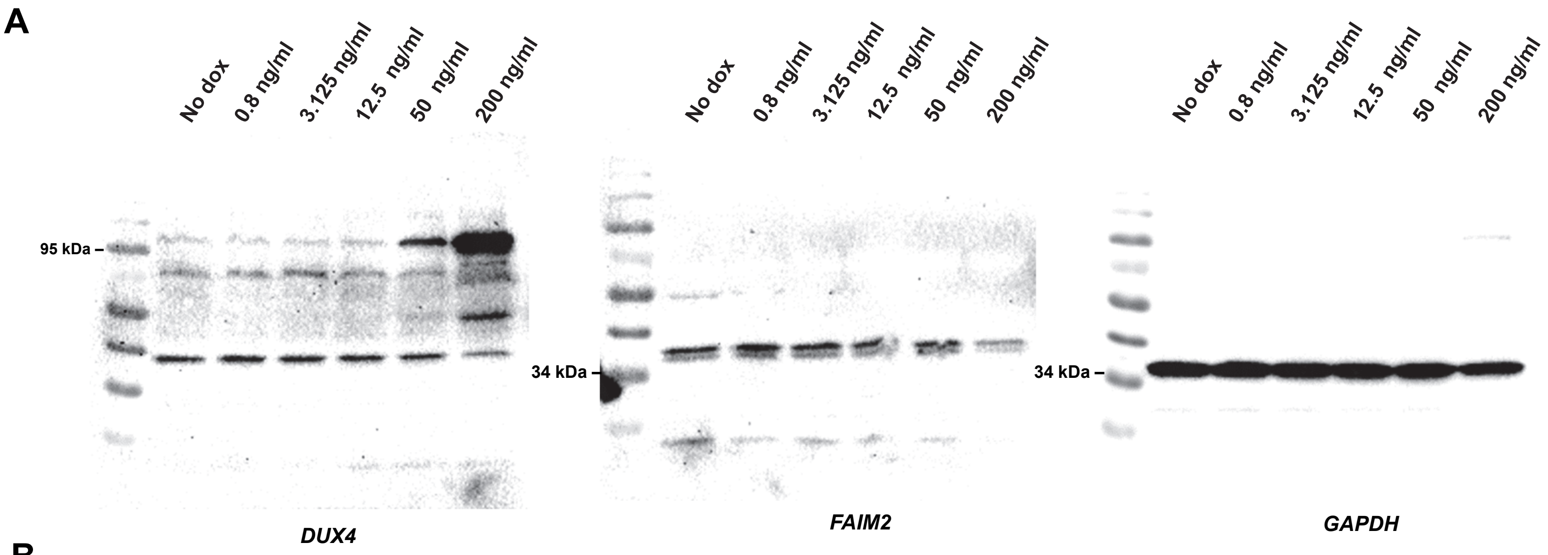

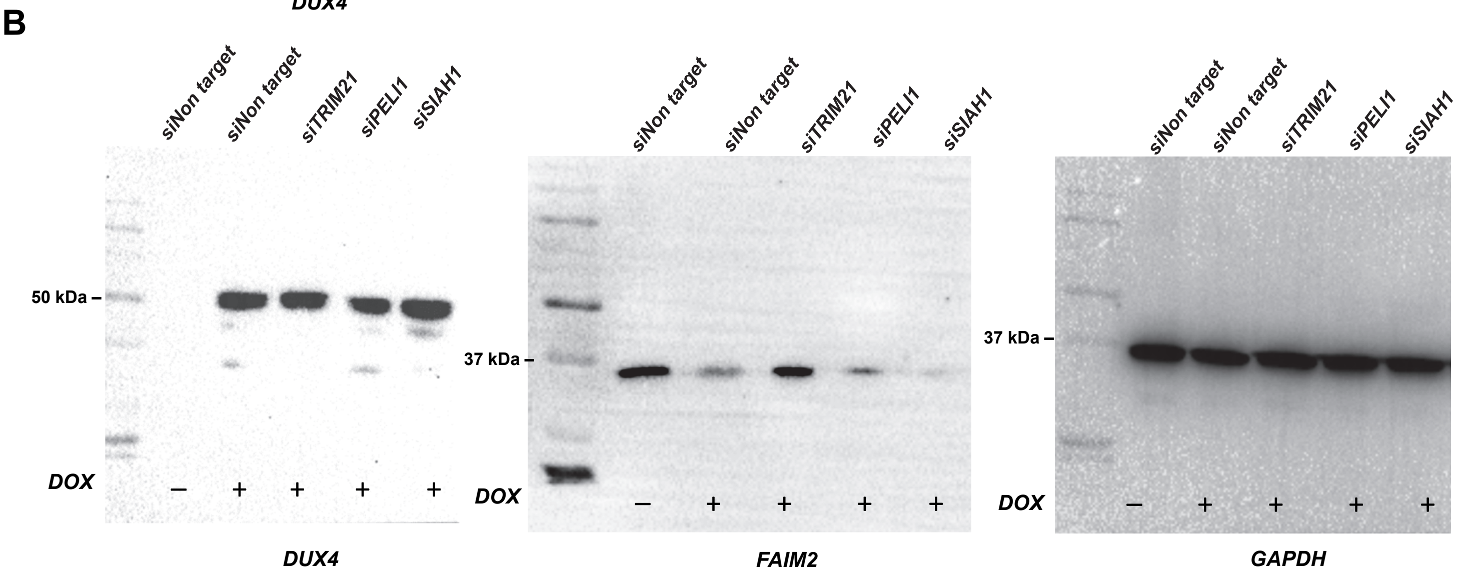

Uncropped western blots from Figure 6 of the main article

A) Western blots of Fig. 6B

B) Western blots of Fig. 6D

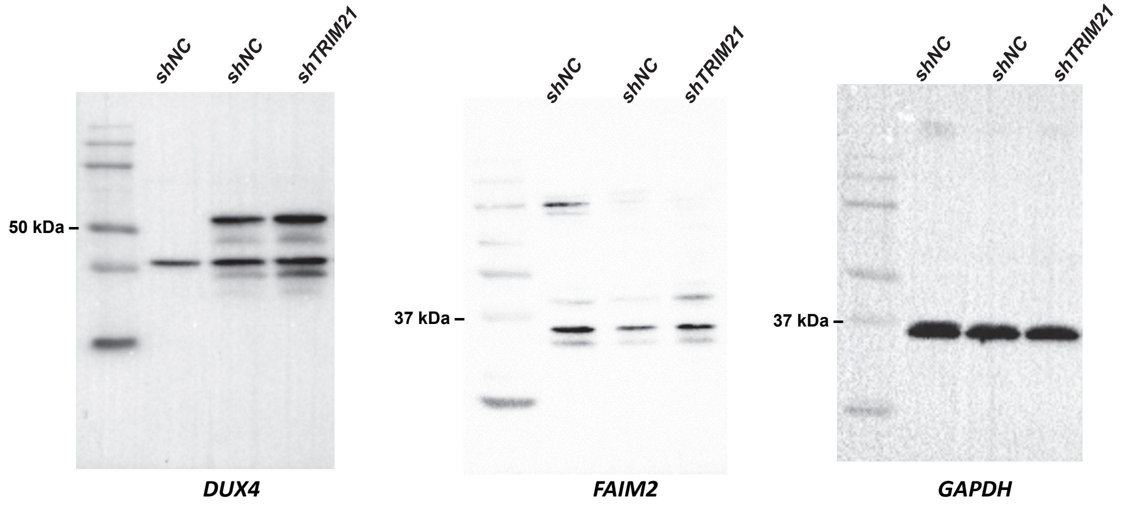

Uncropped western blots from Figure 6F of the main article
