# Supplementary material for: Antiapoptotic Protein FAIM2 is targeted by miR-3202, and DUX4 via TRIM21, leading to cell death and defective myogenesis
Source: Cell Death Dis. 2022 Apr 25;13(4):405. doi: 10.1038/s41419-022-04804-x (PMC9038730; doi:10.1038/s41419-022-04804-x)
Supplement: Supplementary file 2 — Supplemental Material Figures [file 41419_2022_4804_MOESM2_ESM.pdf]

Figure S1

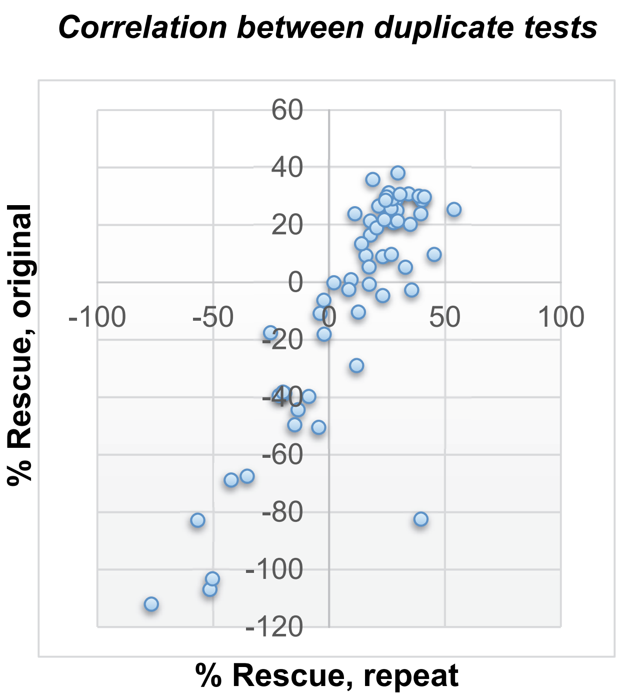

Replicate testing: Plate 19. Correlation coefficient for this data is 0.85.

Figure S2

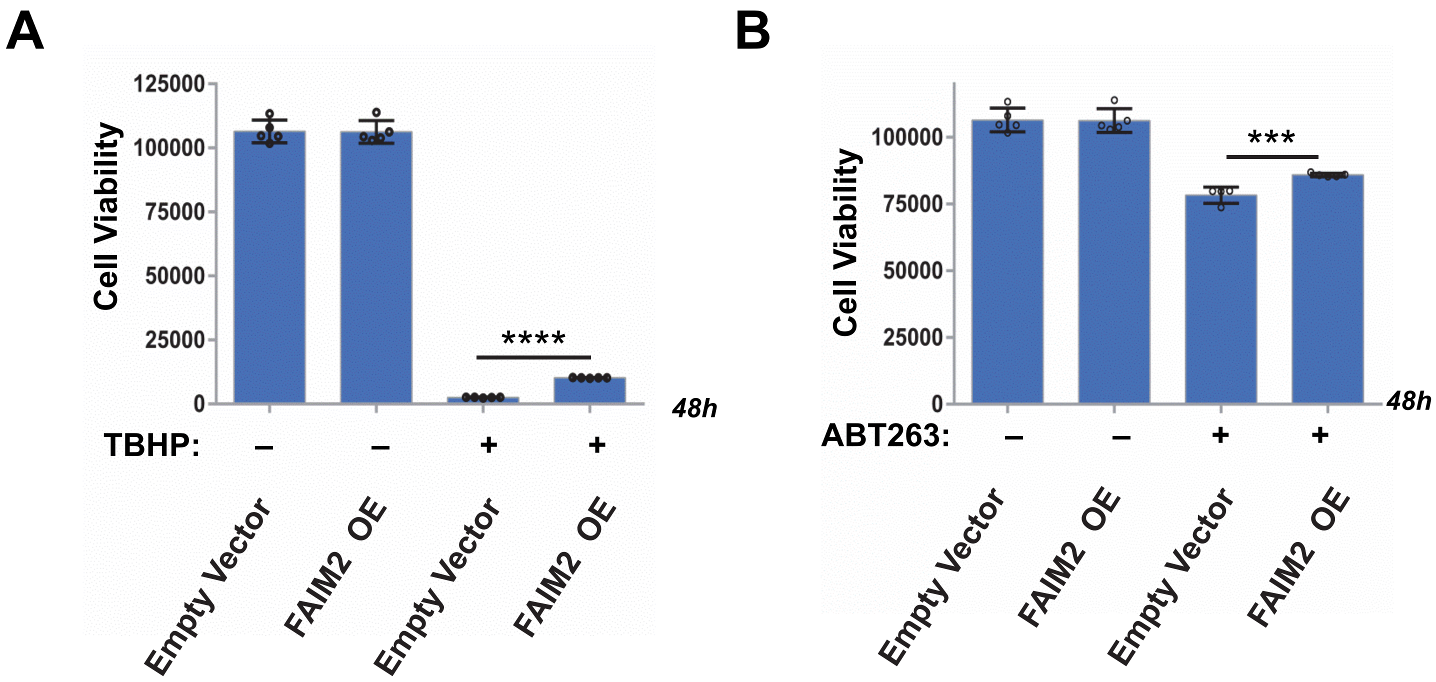

Testing the effect of FAIM2 overexpression on cell death due to oxidative stress or BCL2 inhibition.
(A) LHCN-M2 cells transduced with FAIM2 or an empty vector control exposed to 100 µM tert-butyl hydrogen peroxide (tBHP). **(B)** The same cells exposed to 25 µM ABT263.
