# Supplementary material for: Antiapoptotic Protein FAIM2 is targeted by miR-3202, and DUX4 via TRIM21, leading to cell death and defective myogenesis
Source: Cell Death Dis. 2022 Apr 25;13(4):405. doi: 10.1038/s41419-022-04804-x (PMC9038730; doi:10.1038/s41419-022-04804-x)
Supplement: Supplementary file 3 — Supplemental Table S1 [file 41419_2022_4804_MOESM3_ESM.pdf]

Table S1

Full miR inhibitor screen results

Plate	Panel p	miRBase ID	miRBase accession	microRNA target sequence	% rescue
plate 01	A01				
plate 01	A02				
plate 01	A03				
plate 01	A04				
plate 01	A05				
plate 01	A06				
plate 01	A07				
plate 01	A08				
plate 01	A09				
plate 01	A10				
plate 01	A11				
plate 01	A12				
plate 01	B01				
plate 01	B02				
plate 01	B03				
plate 01	B04	hsa-miR-21-5p	MIMAT0000076	UAGCUUAUCAGACUGAUGUUGA	5.974508644
plate 01	B05	hsa-miR-29a-3p	MIMAT0000086	UAGCACCAUCUGAAAUCGGUUA	-1.984255075
plate 01	B06	hsa-miR-34a-5p	MIMAT0000255	UGGCAGUGUCUUAGCUGGUUGU	5.508482175
plate 01	B07	hsa-miR-125b-5p	MIMAT0000423	UCCCUGAGACCCUAACUUGUGA	-14.3410175
plate 01	B08	hsa-miR-146a-5p	MIMAT0000449	UGAGAACUGAAUUCCAUGGGUU	-1.566646941
plate 01	B09	hsa-miR-155-5p	MIMAT0000646	UUAAUGCUAAUCGUGAUAGGGGU	2.456109676
plate 01	B10	hsa-miR-200c-3p	MIMAT0000617	UAAUACUGCCGGGUAAUGAUGGA	-0.92510401
plate 01	B11	hsa-miR-101-3p	MIMAT0000099	UACAGUACUGUGAUAACUGAA	6.141955383
plate 01	B12				
plate 01	C01				
plate 01	C02	hsa-miR-145-5p	MIMAT0000437	GUCCAGUUUUCCCAGGAAUCCCU	-13.39887741
plate 01	C03	hsa-miR-20a-5p	MIMAT0000075	UAAAGUGCUUAUAGUGCAGGUAG	-12.44261531
plate 01	C04	hsa-miR-126-3p	MIMAT0000445	UCGUACCGUGAGUAAUAAUGCG	1.901316261
plate 01	C05	hsa-miR-181a-5p	MIMAT0000256	AACAUUCAACGCUGUCGGUGAGU	8.002026397
plate 01	C06	hsa-let-7a-5p	MIMAT0000062	UGAGGUAGUAGGUUGUAUAGUU	1.072152285
plate 01	C07	hsa-miR-221-3p	MIMAT0000278	AGCUACAUUGUCUGCUGGGUUUC	7.201106449
plate 01	C08	hsa-miR-222-3p	MIMAT0000279	AGCUACAUCUGGCUACUGGGU	9.702720393
plate 01	C09	hsa-miR-27a-3p	MIMAT0000084	UUCACAGUGGCUAAGUUCCGC	7.540034789
plate 01	C10	hsa-miR-210-3p	MIMAT0000267	CUGUGCGUGUGACAGCGGCUGA	-0.214968438
plate 01	C11	hsa-miR-205-5p	MIMAT0000266	UCCUUCAUUCCACCGGAGUCUG	-24.12353848
plate 01	C12				
plate 01	D01				
plate 01	D02	hsa-miR-17-5p	MIMAT0000070	CAAAGUGCUUACAGUGCAGGUAG	0.640422136
plate 01	D03	hsa-miR-133a-3p	MIMAT0000427	UUUGGUCCCCUUCAACCAGCUG	-34.08762822
plate 01	D04	hsa-miR-125a-5p	MIMAT0000443	UCCCUGAGACCCUUUAACCUGUGA	-22.1666308
plate 01	D05	hsa-miR-122-5p	MIMAT0000421	UGGAGUGUGACAAUGGUGUUUG	9.656319489
plate 01	D06	hsa-miR-204-5p	MIMAT0000265	UUCCCUUUGUCAUCCUAUGCCU	-1.183335127
plate 01	D07	hsa-miR-143-3p	MIMAT0000435	UGAGAUGAAGCACUGUAGCUC	9.262920522
plate 01	D08	hsa-miR-132-3p	MIMAT0000426	UAACAGUCUACAGCCAUGGUCG	-10.37474894
plate 01	D09	hsa-let-7b-5p	MIMAT0000063	UGAGGUAGUAGGUUGUGUGGUU	0.967245893
plate 01	D10	hsa-miR-148a-3p	MIMAT0000243	UCAGUGCACUACAGAACUUUGU	12.83377268

plate	01	D11	hsa-miR-15a-5p	MIMAT0000068	UAGCAGCACAUAAUGGUUUGUG	3.670602898
plate	01	D12				
plate	01	E01				
plate	01	E02	hsa-miR-223-3p	MIMAT0000280	UGUCAGUUUGUCAAAUACCCCA	-11.78089807
plate	01	E03	hsa-miR-106b-5p	MIMAT0000680	UAAAGUGCUGACAGUGCAGAU	2.161564809
plate	01	E04	hsa-miR-18a-5p	MIMAT0000072	UAAGGUGCAUCUAGUGCAGAUAG	3.228785596
plate	01	E05	hsa-miR-19a-3p	MIMAT0000073	UGUGCAAAUCUAUGCAAAACUGA	-0.92712144
plate	01	E06	hsa-miR-200a-3p	MIMAT0000682	UAACACUGUCUGGUAACGAUGU	13.19892762
plate	01	E07	hsa-miR-375	MIMAT0000728	UUUGUUCGUUCGGCUCGCGUGA	9.367826913
plate	01	E08	hsa-miR-130a-3p	MIMAT0000425	CAGUGCAAUGUUAAAAGGGCAU	4.154786242
plate	01	E09	hsa-miR-182-5p	MIMAT0000259	UUUGGCAAUGGUAGAACUCACACU	3.763404706
plate	01	E10	hsa-miR-199a-5p	MIMAT0000231	CCCAGUGUUCAGACUACCUGUUC	-24.86797038
plate	01	E11	hsa-miR-22-3p	MIMAT0000077	AAGCUGCCAGUUGAAGAACUGU	0.503236855
plate	01	E12				
plate	01	F01				
plate	01	F02	hsa-miR-128-3p	MIMAT0000424	UCACAGUGAACCGGUCUCUUU	2.793020587
plate	01	F03	hsa-miR-141-3p	MIMAT0000432	UAACACUGUCUGGUAAAGAUGG	12.63001219
plate	01	F04	hsa-miR-93-5p	MIMAT0000093	CAAAGUGCUGUUCGUGCAGGUAG	12.81359838
plate	01	F05	hsa-miR-195-5p	MIMAT0000461	UAGCAGCACAGAAAUAUUGGC	16.8303027
plate	01	F06	hsa-let-7c-5p	MIMAT0000064	UGAGGUAGUAGGUUGUAUGGUU	5.964421491
plate	01	F07	hsa-let-7g-5p	MIMAT0000414	UGAGGUAGUAGUUUGUACAGUU	2.151477656
plate	01	F08	hsa-miR-10a-5p	MIMAT0000253	UACCCUGUAGAUCCGAAUUUGUG	4.836677785
plate	01	F09	hsa-miR-107	MIMAT0000104	AGCAGCAUUGUACAGGGCUAUCA	9.809644215
plate	01	F10	hsa-miR-23a-3p	MIMAT0000078	AUCACAUUGCCAGGGAUUUCC	9.250815939
plate	01	F11	hsa-miR-29c-3p	MIMAT0000681	UAGCACCAUUUGAAAUCGGUUA	2.661887598
plate	01	F12				
plate	01	G01				
plate	01	G02	hsa-miR-25-3p	MIMAT0000081	CAUUGCACUUGUCUCGGUCUGA	-16.92131124
plate	01	G03	hsa-miR-26a-5p	MIMAT0000082	UUCAAGUAAUCCAGGAUAGGCU	10.17883401
plate	01	G04	hsa-miR-142-3p	MIMAT0000434	UGUAGUGUUUCCUACUUUAUGGA	13.47733305
plate	01	G05	hsa-miR-23b-3p	MIMAT0000418	AUCACAUUGCCAGGGAUUACC	-38.10029768
plate	01	G06	hsa-miR-150-5p	MIMAT0000451	UCUCCCAACCCUUGUACCAGUG	-25.67292518
plate	01	G07	hsa-miR-10b-5p	MIMAT0000254	UACCCUGUAGAACCGAAUUUGUG	2.853543505
plate	01	G08	hsa-miR-199a-3p	MIMAT0000232	ACAGUAGUCUGCACAUUGGUUA	-2.012499103
plate	01	G09	hsa-miR-30a-5p	MIMAT0000087	UGUAAACAUCCUCGACUGGAAG	13.6125009
plate	01	G10	hsa-miR-26b-5p	MIMAT0000083	UUCAAGUAAUUCAGGAUAGGU	9.08740406
plate	01	G11	hsa-miR-133b	MIMAT0000770	UUUGGUCCCCUUCAACCAGCUA	-49.355543
plate	01	G12				
plate	01	H01				
plate	01	H02				
plate	01	H03				
plate	01	H04				
plate	01	H05				
plate	01	H06				
plate	01	H07				
plate	01	H08				
plate	01	H09				
plate	01	H10				
plate	01	H11				

plate	01	H12				
plate	02	A01				
plate	02	A02				
plate	02	A03				
plate	02	A04				
plate	02	A05				
plate	02	A06				
plate	02	A07				
plate	02	A08				
plate	02	A09				
plate	02	A10				
plate	02	A11				
plate	02	A12				
plate	02	B01				
plate	02	B02				
plate	02	B03				
plate	02	B04	hsa-miR-106a-5p	MIMAT0000103	AAAAGUGCUUACAGUGCAGGUAG	-47.1967695
plate	02	B05	hsa-miR-27b-3p	MIMAT0000419	UUCACAGUGGCUAAGUUCUGC	20.39857395
plate	02	B06	hsa-miR-335-5p	MIMAT0000765	UCAAGAGCAAUAACGAAAAAUGU	11.13337138
plate	02	B07	hsa-let-7d-5p	MIMAT0000065	AGAGGUAGUAGGUUGCAUAGUU	-1.913905524
plate	02	B08	hsa-miR-192-5p	MIMAT0000222	CUGACCUAUGAAUUGACAGCC	5.453223461
plate	02	B09	hsa-miR-193b-3p	MIMAT0002819	AACUGGCCCUCAAAGUCCCGCU	-4.112785952
plate	02	B10	hsa-miR-320a	MIMAT0000510	AAAAGCUGGGUUGAGAGGGCGA	-17.30958673
plate	02	B11	hsa-miR-30c-5p	MIMAT0000244	UGUAAACAUCCUACACUCUCAGC	-16.49688052
plate	02	B12				
plate	02	C01				
plate	02	C02	hsa-miR-424-5p	MIMAT0001341	CAGCAGCAAUUCAUGUUUUGAA	8.702289181
plate	02	C03	hsa-miR-144-3p	MIMAT0000436	UACAGUAUAGAUGAUGUACU	13.56797179
plate	02	C04	hsa-let-7e-5p	MIMAT0000066	UGAGGUAGGAGGUUGUAUAGUU	-7.843846262
plate	02	C05	hsa-miR-15b-5p	MIMAT0000417	UAGCAGCACAUCAUGGUUUACA	22.46728066
plate	02	C06	hsa-miR-146b-5p	MIMAT0002809	UGAGAACUGAAUUCCAUAGGCU	26.58710303
plate	02	C07	hsa-miR-30b-5p	MIMAT0000420	UGUAAACAUCCUACACUCAGCU	15.08959705
plate	02	C08	hsa-miR-140-3p	MIMAT0004597	UACCACAGGGUAGAACCACGG	2.402936532
plate	02	C09	hsa-miR-130b-3p	MIMAT0000691	CAGUGCAAUGAUGAAAGGGCAU	0.1213782
plate	02	C10	hsa-miR-20b-5p	MIMAT0001413	CAAAGUGCUCAUAGUGCAGGUAG	-5.588674495
plate	02	C11	hsa-miR-99a-5p	MIMAT0000097	AACCCGUAGAUCCGAUCUUGUG	10.75164574
plate	02	C12				
plate	02	D01				
plate	02	D02	hsa-miR-148b-3p	MIMAT0000759	UCAGUGCAUCACAGAACUUUGU	10.09549982
plate	02	D03	hsa-miR-32-5p	MIMAT0000090	UAUUGCACAUUACUAAGUUGCA	7.768204775
plate	02	D04	hsa-miR-29b-3p	MIMAT0000100	UAGCACCAUUUGAAAUCAGUGUU	6.908002752
plate	02	D05	hsa-miR-33a-5p	MIMAT0000091	GUGCAUUGUAGUUGCAUUGCA	16.80120557
plate	02	D06	hsa-miR-191-5p	MIMAT0000440	CAACGGAAUCCCAAAAGCAGCUG	1.287664379
plate	02	D07	hsa-let-7f-5p	MIMAT0000067	UGAGGUAGUAGAUUGUAUAGUU	-1.479406752
plate	02	D08	hsa-miR-328-3p	MIMAT0000752	CUGGCCCUCUCUGCCCUUCCGU	2.471541601
plate	02	D09	hsa-miR-24-3p	MIMAT0000080	UGGCUCAGUUCAGCAGGAACAG	5.177044079
plate	02	D10	hsa-miR-331-3p	MIMAT0000760	GCCCCUGGGCCUAUCCUAGAA	-4.03890357
plate	02	D11	hsa-miR-140-5p	MIMAT0000431	CAGUGGUUUUACCCUAUGGUAG	6.89920723
plate	02	D12				

plate	02	E01				
plate	02	E02	hsa-let-7i-5p	MIMAT0000415	UGAGGUAGUAGUUUGUGCUGUU	-6.786624552
plate	02	E03	hsa-miR-215-5p	MIMAT0000272	AUGACCUAUGAAUUGACAGAC	-15.85480744
plate	02	E04	hsa-miR-19b-3p	MIMAT0000074	UGUGCAAAUCCAUGCAAAACUGA	-65.32609885
plate	02	E05	hsa-miR-9-5p	MIMAT0000441	UCUUUGGUUAUCUAGCUGUAUGA	14.73777618
plate	02	E06	hsa-miR-421	MIMAT0003339	AUCAACAGACAUUAAUUGGGCGC	-57.77250481
plate	02	E07	hsa-miR-124-3p	MIMAT0000422	UAAGGCACGCGGUGAAUGCC	8.568597251
plate	02	E08	hsa-miR-378a-3p	MIMAT0000732	ACUGGACUUGGAGUCAGAAGGC	-31.59175488
plate	02	E09	hsa-miR-185-5p	MIMAT0000455	UGGAGAGAAAGGCAGUUCCUGA	-25.20268791
plate	02	E10	hsa-miR-326	MIMAT0000756	CCUCUGGGCCCUUCCUCCAG	-5.690702547
plate	02	E11	hsa-miR-346	MIMAT0000773	UGUCUGCCCGCAUGCCUGCCUCU	-65.57589167
plate	02	E12				
plate	02	F01				
plate	02	F02	hsa-miR-200b-3p	MIMAT0000318	UAAUACUGCCUGGUAAUGAUGA	6.288798024
plate	02	F03	hsa-miR-30d-5p	MIMAT0000245	UGUAAACAUCCCCGACUGGAAG	10.24678279
plate	02	F04	hsa-miR-31-5p	MIMAT0000089	AGGCAAGAUGCUGGCAUAGCU	-11.27761794
plate	02	F05	hsa-miR-338-3p	MIMAT0000763	UCCAGCAUCAGUGAUUUUGUUG	-6.382030554
plate	02	F06	hsa-miR-425-5p	MIMAT0003393	AAUGACACGAUCACUCCCGUUGA	-32.58740794
plate	02	F07	hsa-miR-214-3p	MIMAT0000271	ACAGCAGGCACAGACAGGCAGU	-54.83655966
plate	02	F08	hsa-miR-196a-5p	MIMAT0000226	UAGGUAGUUUCAUGUUGUUGGG	8.526378747
plate	02	F09	hsa-miR-301a-3p	MIMAT0000688	CAGUGCAAUAGUAUUGUCAAAGC	-2.003619846
plate	02	F10	hsa-miR-96-5p	MIMAT0000095	UUUGGCACUAGCACAUUUUUGCU	3.78383344
plate	02	F11	hsa-miR-142-5p	MIMAT0000433	CAUAAAGUAGAAAGCACUACU	-3.205088112
plate	02	F12				
plate	02	G01				
plate	02	G02	hsa-miR-206	MIMAT0000462	UGGAAUGUAAGGAAGUGUGUGG	0.131932826
plate	02	G03	hsa-miR-152-3p	MIMAT0000438	UCAGUGCAUGACAGAACUUGG	3.785592545
plate	02	G04	hsa-miR-296-5p	MIMAT0000690	AGGGCCCCCCCUCAAUCCUGU	5.20167154
plate	02	G05	hsa-miR-486-5p	MIMAT0002177	UCCUGUACUGAGCUGCCCCGAG	11.65934358
plate	02	G06	hsa-miR-218-5p	MIMAT0000275	UUGUGCUUGAUCUAACCAUGU	8.626647694
plate	02	G07	hsa-miR-103a-3p	MIMAT0000101	AGCAGCAUUGUACAGGGCUAUGA	23.3362782
plate	02	G08	hsa-miR-183-5p	MIMAT0000261	UAUGGCACUGGUAGAAUUCACU	18.42485888
plate	02	G09	hsa-miR-186-5p	MIMAT0000456	CAAAGAAUUCUCCUUUUGGGCU	15.80731162
plate	02	G10	hsa-miR-92a-3p	MIMAT0000092	UAUUGCACUUGUCCCGGCCUGU	3.368684815
plate	02	G11	hsa-miR-365a-3p	MIMAT0000710	UAAUGCCCCUAAAAAUCCUUAU	9.627578065
plate	02	G12				
plate	02	H01				
plate	02	H02				
plate	02	H03				
plate	02	H04				
plate	02	H05				
plate	02	H06				
plate	02	H07				
plate	02	H08				
plate	02	H09				
plate	02	H10				
plate	02	H11				
plate	02	H12				
plate	03	A01				

plate 03	A02				
plate 03	A03				
plate 03	A04				
plate 03	A05				
plate 03	A06				
plate 03	A07				
plate 03	A08				
plate 03	A09				
plate 03	A10				
plate 03	A11				
plate 03	A12				
plate 03	B01				
plate 03	B02				
plate 03	B03				
plate 03	B04	hsa-miR-181b-5p	MIMAT0000257	AACAUUCAUUGCUGUCGGUGGGU	-8.196036055
plate 03	B05	hsa-miR-30e-5p	MIMAT0000692	UGUAAACAUCCUUGACUGGAAG	12.25781205
plate 03	B06	hsa-miR-92b-3p	MIMAT0003218	UAUUGCACUCGUCCCGGCCUCC	-60.97955771
plate 03	B07	hsa-miR-100-5p	MIMAT0000098	AACCCGUAGAUCCGAACUUGUG	-3.417423747
plate 03	B08	hsa-miR-137	MIMAT0000429	UUAUUGCUUAAGAAUACGCGUAG	-3.983066298
plate 03	B09	hsa-miR-451a	MIMAT0001631	AAACCGUUACCAUUACUGAGUU	-17.18228187
plate 03	B10	hsa-miR-34c-5p	MIMAT0000686	AGGCAGUGUAGUUAGCUGAUUGC	-33.32443958
plate 03	B11	hsa-miR-99b-5p	MIMAT0000689	CACCCGUAGAACCGACCUUGCG	-6.008173357
plate 03	B12				
plate 03	C01				
plate 03	C02	hsa-miR-138-5p	MIMAT0000430	AGCUGGUGUUGUGAAUCAGGCCG	-4.361940083
plate 03	C03	hsa-miR-429	MIMAT0001536	UAAUACUGUCUGGUAAAACCGU	8.274301062
plate 03	C04	hsa-miR-194-5p	MIMAT0000460	UGUAACAGCAACUCCAUGUGGA	-39.35973888
plate 03	C05	hsa-miR-203a	MIMAT0000264	GUGAAAUGUUUAGGACCACUAG	-1.749845471
plate 03	C06	hsa-miR-98-5p	MIMAT0000096	UGAGGUAGUAAGUUGUAUUGUU	3.469007502
plate 03	C07	hsa-miR-18b-5p	MIMAT0001412	UAAGGUGCAUCUAGUGCAGUUAG	-4.804848873
plate 03	C08	hsa-miR-497-5p	MIMAT0002820	CAGCAGCACACUGUGGUUUGU	-6.323012136
plate 03	C09	hsa-miR-127-3p	MIMAT0000446	UCGGAUCCGUCUGAGCUUGGCU	-6.285658382
plate 03	C10	hsa-miR-374a-5p	MIMAT0000727	UUAUAAUACAACCUGAUAAGUG	-19.14602205
plate 03	C11	hsa-miR-342-3p	MIMAT0000753	UCUCACACAGAAAUCGCACCCGU	-51.08348119
plate 03	C12				
plate 03	D01				
plate 03	D02	hsa-miR-139-5p	MIMAT0000250	UCUACAGUGCACGUGUCUCCAGU	-20.71221156
plate 03	D03	hsa-miR-409-3p	MIMAT0001639	GAAUGUUGCUCGGUGAACCCCU	-7.438288487
plate 03	D04	hsa-miR-197-3p	MIMAT0000227	UUCACCACCUUCUCCACCCAGC	-100.5158375
plate 03	D05	hsa-miR-495-3p	MIMAT0002817	AAACAAACAUGGUGCACUUCUU	-41.43020407
plate 03	D06	hsa-miR-28-5p	MIMAT0000085	AAGGAGCUCACAGUCUAUUGAG	-5.074329522
plate 03	D07	hsa-miR-34b-3p	MIMAT0004676	CAAUCACUAACUCCACUGCCAU	-0.183655954
plate 03	D08	hsa-miR-135b-5p	MIMAT0000758	UAUGGCUUUUCAUUCCUAUGUGA	-8.74833798
plate 03	D09	hsa-miR-423-5p	MIMAT0004748	UGAGGGGCAGAGAGCGAGACUUU	-7.577030999
plate 03	D10	hsa-miR-363-3p	MIMAT0000707	AAUUGCACGGUAUCCAUCUGUA	-18.98860266
plate 03	D11	hsa-miR-135a-5p	MIMAT0000428	UAUGGCUUUUUAUUCCUAUGUGA	2.409761781
plate 03	D12				
plate 03	E01				
plate 03	E02	hsa-miR-584-5p	MIMAT0003249	UUAUGGUUUGCCUGGGACUGAG	-38.75407445

plate	03	E03	hsa-miR-181c-5p	MIMAT0000258	AACAUUCAACCUGUCGGUGAGU	0.237907834
plate	03	E04	hsa-miR-532-5p	MIMAT0002888	CAUGCCUUGAGUGUAGGACCGU	8.506427958
plate	03	E05	hsa-miR-373-3p	MIMAT0000726	GAAGUGCUUCGAUUUUGGGGUGU	16.09724427
plate	03	E06	hsa-miR-208a-3p	MIMAT0000241	AUAAGACGAGCAAAAAGCUUGU	5.189948283
plate	03	E07	hsa-miR-885-5p	MIMAT0004947	UCCAUUACACUACCCUGCCUCU	-90.55839414
plate	03	E08	hsa-miR-574-3p	MIMAT0003239	CACGCUCAUGCACACACCCACA	-53.57284204
plate	03	E09	hsa-miR-382-5p	MIMAT0000737	GAAGUUGUUCGUGGUGGAUUCG	2.257678642
plate	03	E10	hsa-miR-181d-5p	MIMAT0002821	AACAUUCAUUGUUGUCGGUGGGU	2.428438658
plate	03	E11	hsa-miR-129-5p	MIMAT0000242	CUUUUUGCGGUCUGGGCUUGC	-19.7223371
plate	03	E12				
plate	03	F01				
plate	03	F02	hsa-miR-324-5p	MIMAT0000761	CGCAUCCCCUAGGGCAUUGGUGU	9.656389938
plate	03	F03	hsa-miR-153-3p	MIMAT0000439	UUGCAUAGUCACAAAAGUGAUC	-25.36809011
plate	03	F04	hsa-miR-134-5p	MIMAT0000447	UGUGACUGGUUGACCAGAGGGG	-26.95562463
plate	03	F05	hsa-miR-339-5p	MIMAT0000764	UCCCUGUCCUCCAGGAGCUCACG	3.292911236
plate	03	F06	hsa-miR-340-5p	MIMAT0004692	UUAUAAAGCAAUGAGACUGAUU	0.896934769
plate	03	F07	hsa-miR-503-5p	MIMAT0002874	UAGCAGCGGGAACAGUUCUGCAG	26.14006768
plate	03	F08	hsa-miR-190a-5p	MIMAT0000458	UGAUAUGUUUGAUAUAUUAGGU	-12.84924648
plate	03	F09	hsa-miR-483-3p	MIMAT0002173	UCACUCCUCUCCUCCCGUCUU	-6.347025263
plate	03	F10	hsa-miR-449a	MIMAT0001541	UGGCAGUGUAUUGUUAGCUGGU	-18.54836199
plate	03	F11	hsa-miR-184	MIMAT0000454	UGGACGGAGAACUGAUAAGGGU	-0.610555993
plate	03	F12				
plate	03	G01				
plate	03	G02	hsa-miR-504-5p	MIMAT0002875	AGACCCUGGUCUGCACUCUAUC	-30.03464116
plate	03	G03	hsa-miR-302b-3p	MIMAT0000715	UAAGUGCUUCCAUGUUUUAGUAG	6.230517127
plate	03	G04	hsa-miR-485-3p	MIMAT0002176	GUCAUACACGGCUCUCCUCUCU	-11.87004451
plate	03	G05	hsa-miR-376a-3p	MIMAT0000729	AUCAUAGAGGAAAAUCCACGU	12.67937584
plate	03	G06	hsa-miR-370-3p	MIMAT0000722	GCCUGCUGGGGUGGAACCUGGU	16.34537992
plate	03	G07	hsa-miR-590-5p	MIMAT0003258	GAGCUUAUUCAUAAAAGUGCAG	22.58345673
plate	03	G08	hsa-miR-494-3p	MIMAT0002816	UGAAACAUACACGGGAAACCUC	-23.36432805
plate	03	G09	hsa-miR-126-5p	MIMAT0000444	CAUUAUUACUUUUGGUACGCG	-17.01418998
plate	03	G10	hsa-miR-151a-5p	MIMAT0004697	UCGAGGAGCUCACAGUCUAGU	-43.68743802
plate	03	G11	hsa-miR-151a-5p	MIMAT0004697	UCGAGGAGCUCACAGUCUAGU	-25.31739573
plate	03	G12				
plate	03	H01				
plate	03	H02				
plate	03	H03				
plate	03	H04				
plate	03	H05				
plate	03	H06				
plate	03	H07				
plate	03	H08				
plate	03	H09				
plate	03	H10				
plate	03	H11				
plate	03	H12				
plate	04	A01				
plate	04	A02				
plate	04	A03				

plate	04	A04				
plate	04	A05				
plate	04	A06				
plate	04	A07				
plate	04	A08				
plate	04	A09				
plate	04	A10				
plate	04	A11				
plate	04	A12				
plate	04	B01				
plate	04	B02				
plate	04	B03				
plate	04	B04	hsa-miR-302a-3p	MIMAT0000684	UAAGUGCUUCCAUGUUUUGGUGA	10.86169942
plate	04	B05	hsa-miR-28-3p	MIMAT0004502	CACUAGAUUGUGAGCUCCUGGA	0.91516449
plate	04	B06	hsa-miR-9-3p	MIMAT0000442	AUAAAGCUAGAUAACCGAAAGU	-5.737681274
plate	04	B07	hsa-miR-193a-3p	MIMAT0000459	AACUGGCCUACAAAGUCCCAGU	-31.54088015
plate	04	B08	hsa-miR-216a-5p	MIMAT0000273	UAAUCUCAGCUGGCAACUGUGA	10.46212232
plate	04	B09	hsa-miR-372-3p	MIMAT0000724	AAAGUGCUGCGACAUUUGAGCGU	-44.39732963
plate	04	B10	hsa-miR-95-3p	MIMAT0000094	UUCAACGGGUAUUUAUUGAGCA	7.224870601
plate	04	B11	hsa-miR-590-3p	MIMAT0004801	UAAUUUUAUGUAUAAGCUAGU	3.283279592
plate	04	B12				
plate	04	C01				
plate	04	C02	hsa-miR-652-3p	MIMAT0003322	AAUGGCGCCACUAGGGUUGUG	-4.771472252
plate	04	C03	hsa-miR-500a-5p	MIMAT0004773	UAAUCCUUGCUACCUGGGUGAGA	-23.54256575
plate	04	C04	hsa-miR-500a-5p	MIMAT0004773	UAAUCCUUGCUACCUGGGUGAGA	-26.58115767
plate	04	C05	hsa-miR-217	MIMAT0000274	UACUGCAUCAGGAACUGAUUGGA	6.746281086
plate	04	C06	hsa-miR-324-3p	MIMAT0000762	ACUGCCCCAGGUGCUGCUGG	-39.91393917
plate	04	C07	hsa-miR-423-3p	MIMAT0001340	AGCUCGGUCUGAGGCCCCUCAGU	14.8348954
plate	04	C08	hsa-miR-433-3p	MIMAT0001627	AUCAUGAUGGGCUCCUCGGUGU	5.114110239
plate	04	C09	hsa-miR-629-5p	MIMAT0004810	UGGGUUUACGUUGGGAGAACU	8.477781833
plate	04	C10	hsa-miR-374b-5p	MIMAT0004955	AUAUAAUACAACCUGCUAAGUG	-2.760041789
plate	04	C11	hsa-miR-151a-3p	MIMAT0000757	CUAGACUGAAGCUCCUUGAGG	-18.66185569
plate	04	C12				
plate	04	D01				
plate	04	D02	hsa-miR-17-3p	MIMAT0000071	ACUGCAGUGAAGGCACUUGUAG	-58.43670792
plate	04	D03	hsa-miR-505-3p	MIMAT0002876	CGUCAACACUUGCUGGUUUCCU	-36.78278984
plate	04	D04	hsa-miR-1	MIMAT0000416	UGGAAUGUAAAGAAGUAUGUAU	-20.29176904
plate	04	D05	hsa-miR-187-3p	MIMAT0000262	UCGUGUCUUGUGUUGCAGCCGG	4.594885764
plate	04	D06	hsa-miR-605-5p	MIMAT0003273	UAAAUCCCAUGGUGCCUUCUCCU	-107.291216
plate	04	D07	hsa-miR-219a-5p	MIMAT0000276	UGAUUGUCCAAACGCAAUUCU	-3.385368656
plate	04	D08	hsa-miR-199b-5p	MIMAT0000263	CCCAGUGUUUAGACUAUCUGUUC	16.79666091
plate	04	D09	hsa-miR-615-3p	MIMAT0003283	UCCGAGCCUGGGUCUCCCUCUU	-49.03648743
plate	04	D10	hsa-miR-136-5p	MIMAT0000448	ACUCCAUUUGUUUUGAUGAUGGA	3.691886679
plate	04	D11	hsa-miR-154-5p	MIMAT0000452	UAGGUUAUCCGUGUUGCCUUCG	2.983032396
plate	04	D12				
plate	04	E01				
plate	04	E02	hsa-miR-519a-3p	MIMAT0002869	AAAGUGCAUCCUUUUAGAGUGU	3.533861839
plate	04	E03	hsa-miR-16-5p	MIMAT0000069	UAGCAGCACGUAAAUAUUGGCG	-7.205054788
plate	04	E04	hsa-miR-149-5p	MIMAT0000450	UCUGGCUCCGUGUCUUCACUCCC	-23.44775084

plate 04	E05	hsa-miR-212-3p	MIMAT0000269	UAACAGUCUCCAGUCACGGCC	3.886031482
plate 04	E06	hsa-miR-345-5p	MIMAT0000772	GCUGACUCCUAGUCCAGGGCUC	-92.73938568
plate 04	E07	hsa-miR-193a-5p	MIMAT0004614	UGGGUCUUUGCGGGCGAGAUGA	13.11242464
plate 04	E08	hsa-miR-708-5p	MIMAT0004926	AAGGAGCUUACAAUCUAGCUGGG	-26.12288563
plate 04	E09	hsa-miR-766-3p	MIMAT0003888	ACUCCAGCCCCACAGCCUCAGC	-116.5221241
plate 04	E10	hsa-miR-483-5p	MIMAT0004761	AAGACGGGAGGAAAGAAGGGAG	-24.04824523
plate 04	E11	hsa-miR-33b-5p	MIMAT0003301	GUGCAUUGCUGUUGCAUUGC	0.039255377
plate 04	E12				
plate 04	F01				
plate 04	F02	hsa-miR-144-5p	MIMAT0004600	GGAUAUCAUCAUAUACUGUAAG	-25.92196834
plate 04	F03	hsa-miR-208b-3p	MIMAT0004960	AUAAGACGAACAAAAGGUUUGU	-23.20168359
plate 04	F04	hsa-miR-30e-3p	MIMAT0000693	CUUUCAGUCGGAUGUUUACAGC	-9.753769708
plate 04	F05	hsa-miR-499a-5p	MIMAT0002870	UUAAGACUUGCAGUGAUGUUU	-34.28599737
plate 04	F06	hsa-miR-660-5p	MIMAT0003338	UACCCAUUGCAUAUCGGAGUUG	-16.16280572
plate 04	F07	hsa-miR-18a-3p	MIMAT0002891	ACUGCCCUAAGUGCUCCUUCUGG	-23.82926796
plate 04	F08	hsa-miR-410-3p	MIMAT0002171	AAUAUAACACAGAUGGCCUGU	-8.769500704
plate 04	F09	hsa-miR-376c-3p	MIMAT0000720	AACAUAGAGGAAAUUCCACGU	-13.25289116
plate 04	F10	hsa-miR-125a-3p	MIMAT0004602	ACAGGUGAGGUUCUUGGGAGCC	-25.07992169
plate 04	F11	hsa-miR-517a-3p	MIMAT0002852	AUCGUGCAUCCCUUUAGAGUGU	-79.75425883
plate 04	F12				
plate 04	G01				
plate 04	G02	hsa-miR-202-3p	MIMAT0002811	AGAGGUAUAGGGCAUGGGAA	-31.30610039
plate 04	G03	hsa-miR-455-3p	MIMAT0004784	GCAGUCCAUGGGCAUAUACAC	-65.87516288
plate 04	G04	hsa-miR-1246	MIMAT0005898	AAUGGAUUUUUGGAGCAGG	-13.50347341
plate 04	G05	hsa-miR-449b-5p	MIMAT0003327	AGGCAGUGUAUUGUUAGCUGGC	-22.55152425
plate 04	G06	hsa-miR-302d-3p	MIMAT0000718	UAAGUGCUUCCAUGUUUGAGUGU	-16.32308806
plate 04	G07	hsa-miR-532-3p	MIMAT0004780	CCUCCCACACCCAAGGCUUGCA	-55.25589364
plate 04	G08	hsa-miR-422a	MIMAT0001339	ACUGGACUUAGGGUCAGAAGGC	-28.44359328
plate 04	G09	hsa-miR-30a-3p	MIMAT0000088	CUUUCAGUCGGAUGUUUGCAGC	-16.88294749
plate 04	G10	hsa-miR-211-5p	MIMAT0000268	UUCCCUUUGUCAUCCUUCGCCU	-17.57374179
plate 04	G11	hsa-miR-582-5p	MIMAT0003247	UUACAGUUGUUCAACCAGUUACU	-27.72796651
plate 04	G12				
plate 04	H01				
plate 04	H02				
plate 04	H03				
plate 04	H04				
plate 04	H05				
plate 04	H06				
plate 04	H07				
plate 04	H08				
plate 04	H09				
plate 04	H10				
plate 04	H11				
plate 04	H12				
plate 05	A01				
plate 05	A02				
plate 05	A03				
plate 05	A04				
plate 05	A05				

plate	05	A06				
plate	05	A07				
plate	05	A08				
plate	05	A09				
plate	05	A10				
plate	05	A11				
plate	05	A12				
plate	05	B01				
plate	05	B02				
plate	05	B03				
plate	05	B04	hsa-miR-361-5p	MIMAT0000703	UUAUCAGAAUCUCCAGGGGUAC	7.754317348
plate	05	B05	hsa-miR-638	MIMAT0003308	AGGGAUCGCGGGCGGGUGGCGGCCU	8.814047041
plate	05	B06	hsa-miR-299-5p	MIMAT0002890	UGGUUUACCGUCCCACAUACAU	27.25512976
plate	05	B07	hsa-miR-542-5p	MIMAT0003340	UCGGGGAUCAUCAUGUCACGAGA	11.24309038
plate	05	B08	hsa-miR-425-3p	MIMAT0001343	AUCGGGAAUGUCGUGUCCGCCC	-5.146998632
plate	05	B09	hsa-miR-379-5p	MIMAT0000733	UGGUAGACUAUGGAACGUAGG	2.652135625
plate	05	B10	hsa-miR-491-5p	MIMAT0002807	AGUGGGGAACCCUUCCAUGAGG	1.047657242
plate	05	B11	hsa-miR-377-3p	MIMAT0000730	AUCACACAAAGGCAACUUUUGU	5.779231249
plate	05	B12				
plate	05	C01				
plate	05	C02	hsa-miR-675-5p	MIMAT0004284	UGGUGCGGAGAGGGCCCACAGUG	4.615314649
plate	05	C03	hsa-miR-489-3p	MIMAT0002805	GUGACAUCACAUAUACGGCAGC	-8.303861944
plate	05	C04	hsa-miR-361-3p	MIMAT0004682	UCCCCCAGGUGUGAUUCUGAUUU	-86.24608782
plate	05	C05	hsa-miR-487b-3p	MIMAT0003180	AAUCGUACAGGGUCAUCCACUU	-19.37744188
plate	05	C06	hsa-miR-574-5p	MIMAT0004795	UGAGUGUGUGUGUGUGAGUGUGU	11.56755818
plate	05	C07	hsa-miR-519d-3p	MIMAT0002853	CAAAGUGCCUCCCUUUAGAGUG	10.05684802
plate	05	C08	hsa-miR-513a-5p	MIMAT0002877	UUCACAGGGAGGUGUCAU	-13.50278865
plate	05	C09	hsa-miR-339-3p	MIMAT0004702	UGAGCGCCUCGACGACAGAGCCG	4.606384342
plate	05	C10	hsa-miR-378a-5p	MIMAT0000731	CUCCUGACUCCAGGUCCUGUGU	12.50226358
plate	05	C11	hsa-miR-744-5p	MIMAT0004945	UGCGGGGCUAGGGCUAACAGCA	12.9681279
plate	05	C12				
plate	05	D01				
plate	05	D02	hsa-miR-30c-1-3p	MIMAT0004674	CUGGGAGAGGGUUGUUUACUCC	17.29188458
plate	05	D03	hsa-miR-520b	MIMAT0002843	AAAGUGCUUCCUUUUAGAGGG	18.34417235
plate	05	D04	hsa-miR-542-3p	MIMAT0003389	UGUGACAGAUUGAUAACUGAAA	8.273763504
plate	05	D05	hsa-miR-450a-5p	MIMAT0001545	UUUUGCGAUGUGUUCCUAAUAU	12.36235545
plate	05	D06	hsa-miR-320b	MIMAT0005792	AAAAGCUGGGUUGAGAGGGCAA	-2.488744093
plate	05	D07	hsa-miR-381-3p	MIMAT0000736	UAUACAAGGGCAAGCUCUCUGU	15.94043155
plate	05	D08	hsa-miR-432-5p	MIMAT0002814	UCUUGGAGUAGGUCAUUGGGUGG	-14.0133045
plate	05	D09	hsa-miR-376b-3p	MIMAT0002172	AUCAUAGAGGAAAAUCCAUGUU	6.710959884
plate	05	D10	hsa-miR-650	MIMAT0003320	AGGAGGCAGCGCUCUCAGGAC	6.303142558
plate	05	D11	hsa-miR-146b-3p	MIMAT0004766	UGCCCUGUGGACUCAGUUCUGG	-6.272217238
plate	05	D12				
plate	05	E01				
plate	05	E02	hsa-miR-223-5p	MIMAT0004570	CGUGUAUUUGACAAGCUGAGUU	14.09334651
plate	05	E03	hsa-miR-7-1-3p	MIMAT0004553	CAACAAAUCACAGUCUGCCAUA	21.79424739
plate	05	E04	hsa-miR-493-3p	MIMAT0003161	UGAAGGUCUACUGUGUGCCAGG	7.720084507
plate	05	E05	hsa-miR-302c-3p	MIMAT0000717	UAAGUGCUUCCAUGUUUCAGUGG	16.7560662
plate	05	E06	hsa-miR-342-5p	MIMAT0004694	AGGGGUGCUAUCUGUGAUUGA	8.46874186

plate 05	E07	hsa-let-7b-3p	MIMAT0004482	CUAUACAACCUACUGCCUUCCC	-73.66030934
plate 05	E08	hsa-miR-520g-3p	MIMAT0002858	ACAAAGUGCUUCCCUUUAGAGUGU	9.340935119
plate 05	E09	hsa-miR-520g-3p	MIMAT0002858	ACAAAGUGCUUCCCUUUAGAGUGU	8.430043866
plate 05	E10	hsa-miR-20a-3p	MIMAT0004493	ACUGCAUUAUGAGCACUUAAAG	5.750951946
plate 05	E11	hsa-miR-625-5p	MIMAT0003294	AGGGGGAAAGUUCUAUAGUCC	8.566975231
plate 05	E12				
plate 05	F01				
plate 05	F02	hsa-miR-874-3p	MIMAT0004911	CUGCCCUGGCCCGAGGGACCGA	20.49191104
plate 05	F03	hsa-miR-21-3p	MIMAT0004494	CAACACCAGUCGAUGGGCUGU	1.815663592
plate 05	F04	hsa-miR-1290	MIMAT0005880	UGGAUUUUUGGAUCAGGGA	11.24606715
plate 05	F05	hsa-miR-383-5p	MIMAT0000738	AGAUCAGAAGGUGAUUGUGGCU	9.104281999
plate 05	F06	hsa-miR-506-3p	MIMAT0002878	UAAGGCACCCUUCUGAGUAGA	20.53209742
plate 05	F07	hsa-miR-512-3p	MIMAT0002823	AAGUGCUGUCAUAGCUGAGGUC	8.958420328
plate 05	F08	hsa-miR-608	MIMAT0003276	AGGGGUGGUGUUGGGACAGCUCCGU	12.64663687
plate 05	F09	hsa-miR-502-3p	MIMAT0004775	AAUGCACCUGGGCAAGGAUUCA	-6.992595288
plate 05	F10	hsa-miR-431-5p	MIMAT0001625	UGUCUUGCAGGCCGUCAUGCA	14.34934863
plate 05	F11	hsa-miR-34c-3p	MIMAT0004677	AAUCACUAACCACACGGCCAGG	25.73548929
plate 05	F12				
plate 05	G01				
plate 05	G02	hsa-miR-517c-3p	MIMAT0002866	AUCGUGCAUCCUUUUAGAGUGU	20.21060639
plate 05	G03	hsa-miR-1296-5p	MIMAT0005794	UUAGGGCCCUGGCUCCAUCUCC	8.596742919
plate 05	G04	hsa-miR-7-5p	MIMAT0000252	UGGAAGACUAGUGAUUUUGUUGU	26.50200725
plate 05	G05	hsa-miR-320c	MIMAT0005793	AAAAGCUGGGUUGAGAGGGU	21.70047918
plate 05	G06	hsa-miR-598-3p	MIMAT0003266	UACGUCAUCGUUGUCAUCGUCA	31.26037218
plate 05	G07	hsa-miR-129-2-3p	MIMAT0004605	AAGCCCUUACCCCAAAAAGCAU	11.44551066
plate 05	G08	hsa-miR-34b-5p	MIMAT0000685	UAGGCAGUGUCAUUAGCUGAUUG	19.89060375
plate 05	G09	hsa-miR-296-3p	MIMAT0004679	GAGGGUUGGGUGGAGGCUCUCC	14.52795475
plate 05	G10	hsa-miR-486-3p	MIMAT0004762	CGGGGCAGCUCAGUACAGGAU	10.86355236
plate 05	G11	hsa-miR-664a-3p	MIMAT0005949	UAUUCAUUUAUCCCCAGCCUACA	-9.859223642
plate 05	G12				
plate 05	H01				
plate 05	H02				
plate 05	H03				
plate 05	H04				
plate 05	H05				
plate 05	H06				
plate 05	H07				
plate 05	H08				
plate 05	H09				
plate 05	H10				
plate 05	H11				
plate 05	H12				
plate 06	A01				
plate 06	A02				
plate 06	A03				
plate 06	A04				
plate 06	A05				
plate 06	A06				
plate 06	A07				

plate	06	A08				
plate	06	A09				
plate	06	A10				
plate	06	A11				
plate	06	A12				
plate	06	B01				
plate	06	B02				
plate	06	B03				
plate	06	B04	hsa-miR-512-5p	MIMAT0002822	CACUCAGCCUUGAGGGCACUUUC	-93.05454864
plate	06	B05	hsa-miR-661	MIMAT0003324	UGCCUGGGUCUCUGGCCUGCGCGU	13.67662535
plate	06	B06	hsa-miR-323a-3p	MIMAT0000755	CACAUUACACGGUCGACCUCU	-0.967317587
plate	06	B07	hsa-miR-16-2-3p	MIMAT0004518	CCAAUAUUACUGUGCUGCUUUA	18.65967025
plate	06	B08	hsa-miR-622	MIMAT0003291	ACAGUCUGCUGAGGUUGGAGC	11.49117166
plate	06	B09	hsa-miR-149-3p	MIMAT0004609	AGGGAGGGACGGGGGCUGUGC	3.468486904
plate	06	B10	hsa-miR-671-5p	MIMAT0003880	AGGAAGCCCUGGAGGGGCUGGAG	-1.125175398
plate	06	B11	hsa-miR-105-5p	MIMAT0000102	UCAAAUGCUCAGACUCCUGUGGU	-24.25485267
plate	06	B12				
plate	06	C01				
plate	06	C02	hsa-miR-582-3p	MIMAT0004797	UAACUGGUUGAACAACUGAACC	9.028589804
plate	06	C03	hsa-miR-491-3p	MIMAT0004765	CUUAUGCAAGAUUCCCUUCUAC	13.990587
plate	06	C04	hsa-miR-498	MIMAT0002824	UUUCAAGCCAGGGGGCGUUUUUC	-82.11851029
plate	06	C05	hsa-miR-367-3p	MIMAT0000719	AAUUGCACUUUAGCAAUGGUGA	7.125526193
plate	06	C06	hsa-miR-1275	MIMAT0005929	GUGGGGGAGAGGCUGUC	9.949427035
plate	06	C07	hsa-miR-320d	MIMAT0006764	AAAAGCUGGGUUGAGAGGA	3.001929373
plate	06	C08	hsa-miR-1271-5p	MIMAT0005796	CUUGGCACCUAGCAAGCACUCA	-98.49713517
plate	06	C09	hsa-miR-873-5p	MIMAT0004953	GCAGGAACUUGUGAGUCUCCU	6.460769411
plate	06	C10	hsa-miR-337-3p	MIMAT0000754	CUCCUAUAUGAUGCCUUUCUUC	7.09395463
plate	06	C11	hsa-miR-301b	MIMAT0004958	CAGUGCAAUGAUAUUGUCAAAGC	3.603543031
plate	06	C12				
plate	06	D01				
plate	06	D02	hsa-miR-455-5p	MIMAT0003150	UAUGUGCCUUUGGACUACAUCG	-7.343019177
plate	06	D03	hsa-miR-450b-5p	MIMAT0004909	UUUUGCAAUAUGUUCCUGAAUA	21.99222404
plate	06	D04	hsa-miR-539-5p	MIMAT0003163	GGAGAAAUUAUCCUUGGUGUGU	13.13815482
plate	06	D05	hsa-miR-330-3p	MIMAT0000751	GCAAAGCACACGGCCUGCAGAGA	-52.81659261
plate	06	D06	hsa-miR-485-5p	MIMAT0002175	AGAGGCUGGCCGUGAUGAAUUC	-8.391896632
plate	06	D07	hsa-miR-520c-3p	MIMAT0002846	AAAGUGCUUCCUUUUAGAGGGU	15.23590973
plate	06	D08	hsa-miR-29c-5p	MIMAT0004673	UGACCGAUUUCUCCUGGUGUUC	2.612546773
plate	06	D09	hsa-miR-454-3p	MIMAT0003885	UAGUGCAAUAUUGCUUAUAGGGU	17.44416511
plate	06	D10	hsa-miR-92a-1-5p	MIMAT0004507	AGGUUGGGAUCGGUUGCAAUGCU	8.618159495
plate	06	D11	hsa-miR-1224-5p	MIMAT0005458	GUGAGGACUCGGGAGGUGG	-16.48123246
plate	06	D12				
plate	06	E01				
plate	06	E02	hsa-miR-765	MIMAT0003945	UGGAGGAGAAGGAAGGUGAUG	18.84734565
plate	06	E03	hsa-miR-767-5p	MIMAT0003882	UGCACCAUGGUUGUCUGAGCAUG	1.358454163
plate	06	E04	hsa-miR-550a-5p	MIMAT0004800	AGUGCCUGAGGGAGUAAGAGCCC	-7.730647802
plate	06	E05	hsa-miR-550a-5p	MIMAT0004800	AGUGCCUGAGGGAGUAAGAGCCC	-1.344422357
plate	06	E06	hsa-miR-369-5p	MIMAT0001621	AGAUCGACCGUGUUAUAUUCGC	31.68469364
plate	06	E07	hsa-miR-939-5p	MIMAT0004982	UGGGGAGCUGAGGCUCUGGGGGUG	10.00380028
plate	06	E08	hsa-miR-769-5p	MIMAT0003886	UGAGACCUCUGGGUUCUGAGCU	17.36699018

plate 06	E09	hsa-miR-570-3p	MIMAT0003235	CGAAAACAGCAAUUACCUUUGC	-51.24327643
plate 06	E10	hsa-miR-518b	MIMAT0002844	CAAAGCGCUCCCCUUUAGAGGU	4.010465388
plate 06	E11	hsa-miR-24-1-5p	MIMAT0000079	UGCCUACUGAGCUGAUAUCAGU	10.1107928
plate 06	E12				
plate 06	F01				
plate 06	F02	hsa-miR-29b-2-5p	MIMAT0004515	CUGGUUUCACAUGGUGGCUUAG	-32.65990412
plate 06	F03	hsa-miR-516a-5p	MIMAT0004770	UUCUCGAGGAAAGAAGCACUUUC	-22.09220065
plate 06	F04	hsa-miR-106b-3p	MIMAT0004672	CCGCACUGUGGGUACUUGCUGC	-46.3917797
plate 06	F05	hsa-miR-299-3p	MIMAT0000687	UAUGUGGGAUGGUAAACCGCUU	23.35681712
plate 06	F06	hsa-miR-492	MIMAT0002812	AGGACCUGCGGGACAAGAUUCUU	-32.32664874
plate 06	F07	hsa-miR-181a-3p	MIMAT0000270	ACCAUCGACCGUUGAUUGUACC	24.23555893
plate 06	F08	hsa-miR-34a-3p	MIMAT0004557	CAAUCAGCAAGUAUACUGCCCU	-76.86184518
plate 06	F09	hsa-miR-551b-3p	MIMAT0003233	GCGACCCAUACUUGGUUUCAG	-4.803262395
plate 06	F10	hsa-miR-572	MIMAT0003237	GUCCGCUCGGCGGUGGCCCA	15.75508653
plate 06	F11	hsa-miR-548c-5p	MIMAT0004806	AAAAGUAAUUGCGGUUUUUGCC	11.45433817
plate 06	F12				
plate 06	G01				
plate 06	G02	hsa-miR-561-3p	MIMAT0003225	CAAAGUUUAAGAUCCUUGAAGU	21.87821562
plate 06	G03	hsa-miR-411-5p	MIMAT0003329	UAGUAGACCGUAUAGCGUACG	13.96778531
plate 06	G04	hsa-miR-188-5p	MIMAT0000457	CAUCCCUUGCAUGGUGGAGGG	19.72257951
plate 06	G05	hsa-miR-31-3p	MIMAT0004504	UGCUAUGCCAACAUAUUGCCAU	29.62728017
plate 06	G06	hsa-miR-329-3p	MIMAT0001629	AACACACCUGGUUAACCUCUUU	-0.346410196
plate 06	G07	hsa-miR-139-3p	MIMAT0004552	UGGAGACGCGGCCCUGUUGGAGU	4.085886342
plate 06	G08	hsa-miR-760	MIMAT0004957	CGGCUCUGGGUCUGUGGGGA	13.23462348
plate 06	G09	hsa-miR-484	MIMAT0002174	UCAGGCUCAGUCCCCUCCCGAU	-56.91037184
plate 06	G10	hsa-miR-548b-5p	MIMAT0004798	AAAAGUAAUUGUGGUUUUGGCC	17.03198082
plate 06	G11	hsa-miR-490-3p	MIMAT0002806	CAACCUGGAGGACUCCAUGCUG	-1.58296305
plate 06	G12				
plate 06	H01				
plate 06	H02				
plate 06	H03				
plate 06	H04				
plate 06	H05				
plate 06	H06				
plate 06	H07				
plate 06	H08				
plate 06	H09				
plate 06	H10				
plate 06	H11				
plate 06	H12				
plate 07	A01				
plate 07	A02				
plate 07	A03				
plate 07	A04				
plate 07	A05				
plate 07	A06				
plate 07	A07				
plate 07	A08				
plate 07	A09				

plate	07	A10				
plate	07	A11				
plate	07	A12				
plate	07	B01				
plate	07	B02				
plate	07	B03				
plate	07	B04	hsa-miR-22-5p	MIMAT0004495	AGUUCUUCAGUGGCAAGCUUUA	13.39313518
plate	07	B05	hsa-miR-510-5p	MIMAT0002882	UACUCAGGAGAGUGGCAAUCAC	0.313702013
plate	07	B06	hsa-miR-603	MIMAT0003271	CACACACUGCAAUUACUUUUGC	27.43373991
plate	07	B07	hsa-miR-885-3p	MIMAT0004948	AGGCAGCGGGGUGUAGUGGAUA	6.108362609
plate	07	B08	hsa-miR-511-5p	MIMAT0002808	GUGUCUUUUGCUCUGCAGUCA	-12.74204725
plate	07	B09	hsa-miR-615-5p	MIMAT0004804	GGGGGUCCCCGGUGCUCGGAUC	-9.126305356
plate	07	B10	hsa-miR-642a-5p	MIMAT0003312	GUCCCUCUCCAAAUGUGUCUUG	-11.27167186
plate	07	B11	hsa-miR-544a	MIMAT0003164	AUUCUGCAUUUUUAGCAAGUUC	-5.842138042
plate	07	B12				
plate	07	C01				
plate	07	C02	hsa-miR-1285-3p	MIMAT0005876	UCUGGGCAACAAAGUGAGACCU	-90.71338974
plate	07	C03	hsa-miR-592	MIMAT0003260	UUGUGUCAAUAUGCGAUGAUGU	8.320833498
plate	07	C04	hsa-miR-214-5p	MIMAT0004564	UGCCUGUCUACACUUGCUGUGC	-10.61444367
plate	07	C05	hsa-miR-16-1-3p	MIMAT0004489	CCAGUAUUAACUGUGCUGCUGA	21.7574988
plate	07	C06	hsa-miR-340-3p	MIMAT0000750	UCCGUCUCAGUUACUUUAUAGC	-6.201343842
plate	07	C07	hsa-miR-127-5p	MIMAT0004604	CUGAAGCUCAGAGGGCUCUGAU	-52.74612615
plate	07	C08	hsa-miR-193b-5p	MIMAT0004767	CGGGGUUUUGAGGGCGAGAUGA	18.88582606
plate	07	C09	hsa-miR-23b-5p	MIMAT0004587	UGGGUUCCUGGCAUGCUGAUUU	-84.4904453
plate	07	C10	hsa-miR-616-3p	MIMAT0004805	AGUCAUUGGAGGGUUUGAGCAG	-1.300750429
plate	07	C11	hsa-miR-637	MIMAT0003307	ACUGGGGGCUUUCGGGCUCUGCGU	7.933996483
plate	07	C12				
plate	07	D01				
plate	07	D02	hsa-miR-488-3p	MIMAT0004763	UUGAAAGGCUAUUUCUUGGUC	13.46023956
plate	07	D03	hsa-miR-518c-3p	MIMAT0002848	CAAAGCGCUUCUCUUUAGAGUGU	15.09048127
plate	07	D04	hsa-miR-216b-5p	MIMAT0004959	AAAUCUCUGCAGGCAAAUGUGA	-48.9389394
plate	07	D05	hsa-miR-625-3p	MIMAT0004808	GACUAUAGAACUUUCCCCCUCA	-34.42663035
plate	07	D06	hsa-miR-516a-3p	MIMAT0006778	UGCUUCCUUUCAGAGGGU	22.75814353
plate	07	D07	hsa-miR-802	MIMAT0004185	CAGUAACAAAGAUUCAUCCUUGU	35.82573475
plate	07	D08	hsa-miR-1291	MIMAT0005881	UGGCCCUGACUGAAGACCAGCAGU	-28.04974057
plate	07	D09	hsa-miR-331-5p	MIMAT0004700	CUAGGUAUGGUCCCAGGGAUCC	-9.448011649
plate	07	D10	hsa-miR-190b	MIMAT0004929	UGAUAUGUUUGAUAUUGGGUU	-0.448130068
plate	07	D11	hsa-miR-155-3p	MIMAT0004658	CUCCUACAUAUUAGCAUUAACA	-8.443419604
plate	07	D12				
plate	07	E01				
plate	07	E02	hsa-miR-371a-5p	MIMAT0004687	ACUCAAACUGUGGGGGCACU	-25.74056043
plate	07	E03	hsa-miR-758-3p	MIMAT0003879	UUUGUGACCUGGUCCACUAACC	13.29642592
plate	07	E04	hsa-miR-362-5p	MIMAT0000705	AAUCCUUGGAACCUAGGUGUGAGU	-28.80565168
plate	07	E05	hsa-let-7i-3p	MIMAT0004585	CUGCGCAAGCUACUGCCUUGCU	-55.62371986
plate	07	E06	hsa-miR-769-3p	MIMAT0003887	CUGGGAUCUCCGGGGUCUUGGUU	-4.255316816
plate	07	E07	hsa-miR-362-3p	MIMAT0004683	AACACACCUAUUCAAGGAUUCA	18.33912273
plate	07	E08	hsa-miR-519e-3p	MIMAT0002829	AAGUGCCUCCUUUUAGAGUGUU	11.1471121
plate	07	E09	hsa-miR-224-5p	MIMAT0000281	CAAGUCACUAGUGGUUCCGUU	20.27330781
plate	07	E10	hsa-miR-29b-1-5p	MIMAT0004514	GCUGGUUUCAUAUGGUGGUUUAGA	-6.795414973

plate	07	E11	hsa-miR-509-3p	MIMAT0002881	UGAUUGGUACGUCUGUGGGUAG	-4.985570365
plate	07	E12				
plate	07	F01				
plate	07	F02	hsa-miR-181a-2-3p	MIMAT0004558	ACCACUGACCGUUGACUGUACC	-25.02806981
plate	07	F03	hsa-miR-496	MIMAT0002818	UGAGUAUUACAUGGCCAAUCUC	-13.52361592
plate	07	F04	hsa-miR-518c-5p	MIMAT0002847	UCUCUGGAGGGAAGCACUUUCUG	-26.26752718
plate	07	F05	hsa-miR-596	MIMAT0003264	AAGCCUGCCCGGCUCCUCGGG	-42.86994031
plate	07	F06	hsa-miR-409-5p	MIMAT0001638	AGGUUACCCGAGCAACUUUGCAU	-38.47065609
plate	07	F07	hsa-miR-557	MIMAT0003221	GUUUGCACGGGUGGGCCUUGUCU	-71.7011399
plate	07	F08	hsa-miR-1224-3p	MIMAT0005459	CCCCACCUCCUCUCUCCUCAG	-26.23002768
plate	07	F09	hsa-miR-297	MIMAT0004450	AUGUAUGUGUGCAUGUGCAUG	-28.38920979
plate	07	F10	hsa-miR-1228-3p	MIMAT0005583	UCACACCUGCCUCGCCCCCC	-77.77408631
plate	07	F11	hsa-miR-30c-2-3p	MIMAT0004550	CUGGGAGAAGGCUGUUUACUCU	-6.493445262
plate	07	F12				
plate	07	G01				
plate	07	G02	hsa-miR-25-5p	MIMAT0004498	AGGCGGAGACUUGGGCAAUUG	-17.84790112
plate	07	G03	hsa-miR-369-3p	MIMAT0000721	AAUAAUACAUGGUUGAUCUUU	-4.162554879
plate	07	G04	hsa-miR-513a-3p	MIMAT0004777	UAAAUUUCACCUUUCUGAGAAGG	8.048468661
plate	07	G05	hsa-miR-1228-5p	MIMAT0005582	GUGGGCGGGGGCAGGUGUGUG	9.355030416
plate	07	G06	hsa-miR-509-5p	MIMAT0004779	UACUGCAGACAGUGGCAAUCA	-32.25560629
plate	07	G07	hsa-miR-942-5p	MIMAT0004985	UCUUCUCUGUUUUGGCCAUGUG	21.02724526
plate	07	G08	hsa-miR-10a-3p	MIMAT0004555	CAAAUUCGUAUCUAGGGGAAUA	20.69567067
plate	07	G09	hsa-miR-665	MIMAT0004952	ACCAGGAGGCUGAGGCCCCU	-42.70612667
plate	07	G10	hsa-miR-612	MIMAT0003280	GCUGGGCAGGGCUUCUGAGCUCCUU	-15.41240685
plate	07	G11	hsa-miR-363-5p	MIMAT0003385	CGGGUGGAUCACGAUGCAAUUU	-13.60650956
plate	07	G12				
plate	07	H01				
plate	07	H02				
plate	07	H03				
plate	07	H04				
plate	07	H05				
plate	07	H06				
plate	07	H07				
plate	07	H08				
plate	07	H09				
plate	07	H10				
plate	07	H11				
plate	07	H12				
plate	08	A01				
plate	08	A02				
plate	08	A03				
plate	08	A04				
plate	08	A05				
plate	08	A06				
plate	08	A07				
plate	08	A08				
plate	08	A09				
plate	08	A10				
plate	08	A11				

plate	08	A12				
plate	08	B01				
plate	08	B02				
plate	08	B03				
plate	08	B04	hsa-miR-921	MIMAT0004971	CUAGUGAGGGACAGAACCAGGAUUC	-33.74250126
plate	08	B05	hsa-miR-200c-5p	MIMAT0004657	CGUCUUACCCAGCAGUGUUUGG	10.82948374
plate	08	B06	hsa-miR-1207-5p	MIMAT0005871	UGGCAGGGAGGCUGGGAGGGG	9.452323957
plate	08	B07	hsa-miR-200a-5p	MIMAT0001620	CAUCUUACCGGACAGUGCUGGA	-3.075656846
plate	08	B08	hsa-miR-124-5p	MIMAT0004591	CGUGUUCACAGCGGACCUUGAU	8.039691878
plate	08	B09	hsa-let-7e-3p	MIMAT0004485	CUAUACGGCCUCCUAGCUUUCC	23.36789757
plate	08	B10	hsa-miR-1179	MIMAT0005824	AAGCAUUCUUUCAUUGGUUGG	15.19674953
plate	08	B11	hsa-miR-524-3p	MIMAT0002850	GAAGGCGCUUCCCUUUGGAGU	25.10186693
plate	08	B12				
plate	08	C01				
plate	08	C02	hsa-miR-516b-5p	MIMAT0002859	AUCUGGAGGUAAGAAGCACUUU	1.15806618
plate	08	C03	hsa-miR-1202	MIMAT0005865	GUGCCAGCUGCAGUGGGGGAG	-42.95277895
plate	08	C04	hsa-miR-10b-3p	MIMAT0004556	ACAGAUUCGAUUCUAGGGGAAU	8.296344383
plate	08	C05	hsa-miR-636	MIMAT0003306	UGUGCUUGCUCGUCCCGCCCGCA	-59.98991474
plate	08	C06	hsa-miR-92a-2-5p	MIMAT0004508	GGGUGGGGAUUUGUUGCAUUAC	11.27810397
plate	08	C07	hsa-miR-26a-2-3p	MIMAT0004681	CCUAUUCUUGAUUACUUGUUUC	24.53848339
plate	08	C08	hsa-miR-147a	MIMAT0000251	GUGUGUGGAAAUGCUUCUGC	15.86029015
plate	08	C09	hsa-miR-182-3p	MIMAT0000260	UGGUUCUAGACUUGCCAACUA	15.28438697
plate	08	C10	hsa-miR-196b-5p	MIMAT0001080	UAGGUAGUUUCCUGUUGUUGGG	16.26091845
plate	08	C11	hsa-miR-1231	MIMAT0005586	GUGUCUGGGCGGACAGCUGC	40.78270899
plate	08	C12				
plate	08	D01				
plate	08	D02	hsa-miR-424-3p	MIMAT0004749	CAAAACGUGAGGCGCUGCUAU	3.789276006
plate	08	D03	hsa-miR-145-3p	MIMAT0004601	GGAUUCCUGGAAAUACUGUUCU	33.68198969
plate	08	D04	hsa-miR-548c-3p	MIMAT0003285	CAAAAAUCUCAAUUACUUUUGC	24.51135751
plate	08	D05	hsa-miR-520e	MIMAT0002825	AAAGUGCUUCCUUUUUGAGGG	28.04189441
plate	08	D06	hsa-miR-602	MIMAT0003270	GACACGGGCGACAGCUGCGGCCC	2.731366901
plate	08	D07	hsa-miR-15b-3p	MIMAT0004586	CGAAUCAUUAUUUGCUGCUCUA	23.52230639
plate	08	D08	hsa-miR-30b-3p	MIMAT0004589	CUGGGAGGUGGAUGUUUACUUC	-63.0363591
plate	08	D09	hsa-miR-141-5p	MIMAT0004598	CAUCUUCCAGUACAGUGUUGGA	17.24370975
plate	08	D10	hsa-miR-1323	MIMAT0005795	UCAAAACUGAGGGGCAUUUUCU	15.87072318
plate	08	D11	hsa-miR-490-5p	MIMAT0004764	CCAUGGAUCUCCAGGUGGGU	18.67094807
plate	08	D12				
plate	08	E01				
plate	08	E02	hsa-miR-19b-1-5p	MIMAT0004491	AGUUUUGCAGGUUUGCAUCCAGC	-23.01734781
plate	08	E03	hsa-miR-518e-3p	MIMAT0002861	AAAGCGCUUCCCUUCAGAGUG	18.72102661
plate	08	E04	hsa-miR-384	MIMAT0001075	AUUCCUAGAAAUUGUUCAUA	67.12819294
plate	08	E05	hsa-miR-595	MIMAT0003263	GAAGUGUGCCGUGGUGUGUCU	23.58490457
plate	08	E06	hsa-miR-515-3p	MIMAT0002827	GAGUGCCUUCUUUUGGAGCGUU	27.6016206
plate	08	E07	hsa-miR-1270	MIMAT0005924	CUGGAGAUAUGGAAGAGCUGUGU	0.091810652
plate	08	E08	hsa-miR-515-5p	MIMAT0002826	UUCUCCAAAAGAAAGCACUUUCUG	15.77682592
plate	08	E09	hsa-miR-219a-2-3p	MIMAT0004675	AGAAUUGUGGCUGGACAUCUGU	7.91658214
plate	08	E10	hsa-miR-150-3p	MIMAT0004610	CUGGUACAGGCCUGGGGGACAG	1.973929021
plate	08	E11	hsa-miR-520a-5p	MIMAT0002833	CUCCAGAGGGAAGUACUUUCU	12.35479253
plate	08	E12				

plate	08	F01				
plate	08	F02	hsa-miR-300	MIMAT0004903	UAUACAAGGGCAGACUCUCUCU	-57.97008039
plate	08	F03	hsa-miR-657	MIMAT0003335	GGCAGGUUCUCACCCUCUCUAGG	-71.90860667
plate	08	F04	hsa-miR-96-3p	MIMAT0004510	AAUCAUGUGCAGUGCCAAUAUG	-59.85428537
plate	08	F05	hsa-miR-1303	MIMAT0005891	UUUAGAGACGGGGUCUUGCUCU	-16.62816106
plate	08	F06	hsa-miR-585-3p	MIMAT0003250	UGGGCGUAUCUGUAUGCUA	25.12899281
plate	08	F07	hsa-miR-552-3p	MIMAT0003215	AACAGGUGACUGGUUAGACAA	21.76538437
plate	08	F08	hsa-miR-148b-5p	MIMAT0004699	AAGUUCUGUUAUACACUCAGGC	21.10601696
plate	08	F09	hsa-miR-99a-3p	MIMAT0004511	CAAGCUCGCUUCUAUGGGUCUG	24.78887607
plate	08	F10	hsa-miR-654-5p	MIMAT0003330	UGGUGGGCCGCAGAACAUGUGC	0.828382475
plate	08	F11	hsa-miR-589-5p	MIMAT0004799	UGAGAACCACGUCUGCUCUGAG	-27.91669806
plate	08	F12				
plate	08	G01				
plate	08	G02	hsa-miR-195-3p	MIMAT0004615	CCAAUAUUGGCUGUGCUGCUCC	6.992215801
plate	08	G03	hsa-miR-338-5p	MIMAT0004701	AACAAUAUCCUGGUGCUGAGUG	9.302088345
plate	08	G04	hsa-miR-222-5p	MIMAT0004569	CUCAGUAGCCAGUGUAGAUCCU	21.7403451
plate	08	G05	hsa-miR-545-3p	MIMAT0003165	UCAGCAAACAUUUAUUGUGUGC	12.66569678
plate	08	G06	hsa-miR-1908-5p	MIMAT0007881	CGGCGGGGACGGCGAUUGGUC	18.87543543
plate	08	G07	hsa-miR-205-3p	MIMAT0009197	GAUUUCAGUGGAGUGAAGUUC	26.78993097
plate	08	G08	hsa-miR-618	MIMAT0003287	AAACUCUACUUGUCCUUCUGAGU	16.5676495
plate	08	G09	hsa-miR-646	MIMAT0003316	AAGCAGCUGCCUCUGAGGC	-8.363115766
plate	08	G10	hsa-miR-571	MIMAT0003236	UGAGUUGGCCAUCUGAGUGAG	-7.413710159
plate	08	G11	hsa-miR-508-3p	MIMAT0002880	UGAUUGUAGCCUUUUGGAGUAGA	10.11169137
plate	08	G12				
plate	08	H01				
plate	08	H02				
plate	08	H03				
plate	08	H04				
plate	08	H05				
plate	08	H06				
plate	08	H07				
plate	08	H08				
plate	08	H09				
plate	08	H10				
plate	08	H11				
plate	08	H12				
plate	09	A01				
plate	09	A02				
plate	09	A03				
plate	09	A04				
plate	09	A05				
plate	09	A06				
plate	09	A07				
plate	09	A08				
plate	09	A09				
plate	09	A10				
plate	09	A11				
plate	09	A12				
plate	09	B01				

plate	09	B02				
plate	09	B03				
plate	09	B04	hsa-miR-298	MIMAT0004901	AGCAGAAGCAGGGAGGUUCUCCCA	-18.12011089
plate	09	B05	hsa-miR-502-5p	MIMAT0002873	AUCCUUGCUAUCUGGGUGCUA	22.51000843
plate	09	B06	hsa-miR-132-5p	MIMAT0004594	ACCGUGGCUUUCGAUUGUUACU	21.74447234
plate	09	B07	hsa-miR-325	MIMAT0000771	CCUAGUAGGUGUCCAGUAAGUGU	-10.2750017
plate	09	B08	hsa-miR-181c-3p	MIMAT0004559	AACCAUCGACCGUUGAGUGGAC	25.82296948
plate	09	B09	hsa-miR-374a-3p	MIMAT0004688	CUUAUCAGAUUGUAUUGUAAUU	21.30172639
plate	09	B10	hsa-miR-493-5p	MIMAT0002813	UUGUACAUGGUAGGCUUUCAUU	24.01272743
plate	09	B11	hsa-miR-32-3p	MIMAT0004505	CAAUUUAGUGUGUGUGAUAUUU	17.35627114
plate	09	B12				
plate	09	C01				
plate	09	C02	hsa-miR-135b-3p	MIMAT0004698	AUGUAGGGCUAAAAGCCAUGGG	-11.51817997
plate	09	C03	hsa-miR-548d-5p	MIMAT0004812	AAAAGUAAUUGUGGUUUUUGCC	22.53399959
plate	09	C04	hsa-miR-548d-5p	MIMAT0004812	AAAAGUAAUUGUGGUUUUUGCC	28.73462385
plate	09	C05	hsa-miR-188-3p	MIMAT0004613	CUCCCACAUGCAGGGUUUGCA	34.56229462
plate	09	C06	hsa-miR-185-3p	MIMAT0004611	AGGGGCUGGCUUUCCUCUGGUC	-67.2692729
plate	09	C07	hsa-miR-1256	MIMAT0005907	AGGCAUUGACUUCUCACUAGCU	36.02357434
plate	09	C08	hsa-miR-521	MIMAT0002854	AACGCACUUCCCUUUAGAGUGU	-11.45929258
plate	09	C09	hsa-miR-1973	MIMAT0009448	ACCGUGCAAAGGUAGCAUA	-31.9782771
plate	09	C10	hsa-miR-373-5p	MIMAT0000725	ACUCAAAAUGGGGGCGCUUUCC	-7.068910365
plate	09	C11	hsa-miR-580-3p	MIMAT0003245	UUGAGAAUGAUGAAUCAUUAGG	33.116282
plate	09	C12				
plate	09	D01				
plate	09	D02	hsa-miR-639	MIMAT0003309	AUCGCUGCGGUUGCGAGCGCUGU	29.93636284
plate	09	D03	hsa-miR-183-3p	MIMAT0004560	GUGAAUUACCGAAGGGCCAUAA	-84.69557884
plate	09	D04	hsa-miR-376a-5p	MIMAT0003386	GUAGAUUCUCCUUCUAUGAGUA	24.82188381
plate	09	D05	hsa-miR-663b	MIMAT0005867	GGUGGCCCGGCCGUGCCUGAGG	-35.80595756
plate	09	D06	hsa-miR-543	MIMAT0004954	AAACAUUCGCGGUGCACUUCUU	38.17405465
plate	09	D07	hsa-miR-154-3p	MIMAT0000453	AAUCAUACACGGUUGACCUAUU	39.58735205
plate	09	D08	hsa-miR-1227-3p	MIMAT0005580	CGUGCCACCCUUUUCCCCAG	7.480637438
plate	09	D09	hsa-miR-632	MIMAT0003302	GUGUCUGCUUCCUGUGGGA	23.74446265
plate	09	D10	hsa-miR-29a-5p	MIMAT0004503	ACUGAUUUCUUUUGGUGUUCAG	29.14683559
plate	09	D11	hsa-miR-93-3p	MIMAT0004509	ACUGCUGAGCUAGCACUUCCCG	-16.55196146
plate	09	D12				
plate	09	E01				
plate	09	E02	hsa-miR-1258	MIMAT0005909	AGUUAGGAUUAGGUCGUGGAA	16.75213012
plate	09	E03	hsa-let-7d-3p	MIMAT0004484	CUAUACGACCUGCUGCCUUUCU	36.28093406
plate	09	E04	hsa-miR-196a-3p	MIMAT0004562	CGGCAACAAGAAACUGCCUGAG	3.201486967
plate	09	E05	hsa-miR-27a-5p	MIMAT0004501	AGGGCUUAGCUGCUUGUGAGCA	20.18504696
plate	09	E06	hsa-miR-125b-2-3p	MIMAT0004603	UCACAAGUCAGGCUCUUGGGAC	-17.36329886
plate	09	E07	hsa-miR-219a-1-3p	MIMAT0004567	AGAGUUGAGUCUGGACGUCCCG	39.79454843
plate	09	E08	hsa-miR-143-5p	MIMAT0004599	GGUGCAGUGCUGCAUCUCUGGU	-79.37826546
plate	09	E09	hsa-miR-1283	MIMAT0005799	UCUACAAAGGAAAGCGCUUUCU	14.49696112
plate	09	E10	hsa-miR-23a-5p	MIMAT0004496	GGGGUUCCUGGGGAUGGGAUUU	15.56347722
plate	09	E11	hsa-miR-522-3p	MIMAT0002868	AAAAUGGUUCCCUUUAGAGUGU	19.2581158
plate	09	E12				
plate	09	F01				
plate	09	F02	hsa-miR-1282	MIMAT0005940	UCGUUUGCCUUUUUCUGCUU	19.03129029

plate 09	F03	hsa-miR-525-3p	MIMAT0002839	GAAGGCGCUUCCCUUUAGAGCG	33.51104563
plate 09	F04	hsa-miR-576-5p	MIMAT0003241	AUUCUAAUUUCUCCACGUCUUU	34.23514244
plate 09	F05	hsa-miR-517-5p	MIMAT0002851	CCUCUAGAUGGAAGCACUGUCU	-11.08415808
plate 09	F06	hsa-miR-579-3p	MIMAT0003244	UUCAUUUGGUAUAAACCGCGAUU	34.47287303
plate 09	F07	hsa-miR-129-1-3p	MIMAT0004548	AAGCCCUUACCCCAAAAAGUAU	36.62553435
plate 09	F08	hsa-miR-1299	MIMAT0005887	UUCUGGAAUUCUGUGUGAGGGA	23.06834815
plate 09	F09	hsa-miR-99b-3p	MIMAT0004678	CAAGCUCGUGUCUGUGGGUCCG	29.93854386
plate 09	F10	hsa-miR-302b-5p	MIMAT0000714	ACUUUAACAUGGAAGUGCUUUC	-11.67521301
plate 09	F11	hsa-miR-601	MIMAT0003269	UGGUCUAGGAUUGUUGGAGGAG	20.80445509
plate 09	F12				
plate 09	G01				
plate 09	G02	hsa-miR-566	MIMAT0003230	GGGCGCCUGUGAUCCCAAC	13.52859068
plate 09	G03	hsa-miR-643	MIMAT0003313	ACUUGUAUGCUAGCUCAGGUAG	-9.18885647
plate 09	G04	hsa-miR-371a-3p	MIMAT0000723	AAGUGCCGCCAUCUUUUGAGUGU	19.86443783
plate 09	G05	hsa-miR-302f	MIMAT0005932	UAAUUGCUUCCAUGUUU	26.44455861
plate 09	G06	hsa-miR-624-5p	MIMAT0003293	UAGUACCAGUACCUUGUGUUCA	-54.67827613
plate 09	G07	hsa-miR-628-5p	MIMAT0004809	AUGCUGACAUAUUUACUAGAGG	11.90155385
plate 09	G08	hsa-miR-634	MIMAT0003304	AACCAGCACCCCAACUUUGGAC	-40.6063705
plate 09	G09	hsa-miR-454-5p	MIMAT0003884	ACCCUAUCAAUAUUGUCUCUGC	-6.414606012
plate 09	G10	hsa-miR-1297	MIMAT0005886	UUCAAGUAAUUCAGGUG	19.09890174
plate 09	G11	hsa-miR-448	MIMAT0001532	UUGCAUAUGUAGGAUGUCCCAU	0.53410622
plate 09	G12				
plate 09	H01				
plate 09	H02				
plate 09	H03				
plate 09	H04				
plate 09	H05				
plate 09	H06				
plate 09	H07				
plate 09	H08				
plate 09	H09				
plate 09	H10				
plate 09	H11				
plate 09	H12				
plate 10	A01				
plate 10	A02				
plate 10	A03				
plate 10	A04				
plate 10	A05				
plate 10	A06				
plate 10	A07				
plate 10	A08				
plate 10	A09				
plate 10	A10				
plate 10	A11				
plate 10	A12				
plate 10	B01				
plate 10	B02				
plate 10	B03				

plate	10	B04	hsa-miR-519c-3p	MIMAT0002832	AAAGUGCAUCUUUUUAGAGGAU	-11.82140238
plate	10	B05	hsa-miR-452-3p	MIMAT0001636	CUCAUCUGCAAAGAAGUAAGUG	3.258959092
plate	10	B06	hsa-miR-655-3p	MIMAT0003331	AUAAUACAUGGUUAACCUCUUU	-13.34510062
plate	10	B07	hsa-miR-1183	MIMAT0005828	CACUGUAGGUGAUGGUGAGAGUGGGCA	18.45119268
plate	10	B08	hsa-miR-1233-3p	MIMAT0005588	UGAGCCCUGUCCUCCCGCAG	-26.36477769
plate	10	B09	hsa-miR-641	MIMAT0003311	AAAGACAUAGGAUAGAGUCACCUC	20.12479956
plate	10	B10	hsa-miR-1204	MIMAT0005868	UCGUGGCCUGGUCUCCAUUAU	-31.63619187
plate	10	B11	hsa-miR-1468-5p	MIMAT0006789	CUCCGUUUGCCUGUUUCGCUG	-2.533779164
plate	10	B12				
plate	10	C01				
plate	10	C02	hsa-miR-647	MIMAT0003317	GUGGCUGCACUCACUUCCUUC	-55.49851459
plate	10	C03	hsa-miR-200b-5p	MIMAT0004571	CAUCUUACUGGGCAGCAUUGGA	-5.878755485
plate	10	C04	hsa-miR-302c-5p	MIMAT0000716	UUUAACAUGGGGGUACCUGCUG	-68.22284926
plate	10	C05	hsa-miR-1185-5p	MIMAT0005798	AGAGGAUACCCUUUGUAUGUU	15.90945817
plate	10	C06	hsa-miR-944	MIMAT0004987	AAAUUAUUGUACAUCGGAUGAG	-13.01172171
plate	10	C07	hsa-miR-659-3p	MIMAT0003337	CUUGGUUCAGGGAGGGUCCCCA	-11.88181332
plate	10	C08	hsa-miR-520f-3p	MIMAT0002830	AAGUGCUUCCUUUUAGAGGGUU	7.541423759
plate	10	C09	hsa-miR-1225-3p	MIMAT0005573	UGAGCCCCUGUGCCGCCCCCAG	-53.75778443
plate	10	C10	hsa-miR-524-5p	MIMAT0002849	CUACAAAGGGAAGCACUUUCUC	-35.99472958
plate	10	C11	hsa-miR-518a-3p	MIMAT0002863	GAAAGCGCUUCCCUUUGCUGGA	-1.547067086
plate	10	C12				
plate	10	D01				
plate	10	D02	hsa-miR-1286	MIMAT0005877	UGCAGGACCAAGAUGAGCCCU	-42.15664583
plate	10	D03	hsa-miR-3065-3p	MIMAT0015378	UCAGCACCAGGAUAUUGUUGGAG	-77.6491939
plate	10	D04	hsa-miR-1915-3p	MIMAT0007892	CCCCAGGGCGACGCGGCGGG	-3.050628348
plate	10	D05	hsa-miR-92b-5p	MIMAT0004792	AGGGACGGGACGCGGUGCAGUG	13.73913908
plate	10	D06	hsa-miR-19b-2-5p	MIMAT0004492	AGUUUUGCAGGUUUGCAUUUCA	-13.93131052
plate	10	D07	hsa-miR-380-3p	MIMAT0000735	UAUGUAAUAUGGUCCACAUCUU	24.84132805
plate	10	D08	hsa-miR-569	MIMAT0003234	AGUUAAUGAAUCCUGGAAAGU	1.840420639
plate	10	D09	hsa-miR-20b-3p	MIMAT0004752	ACUGUAGUAUGGGCACUUCCAG	-59.44088802
plate	10	D10	hsa-miR-488-5p	MIMAT0002804	CCCAGAUAAUGGCACUCUCAA	-2.10195279
plate	10	D11	hsa-miR-2110	MIMAT0010133	UUGGGGAAACGGCCGCUGAGUG	-3.104326965
plate	10	D12				
plate	10	E01				
plate	10	E02	hsa-miR-487a-3p	MIMAT0002178	AAUCAUACAGGGACAUCCAGUU	14.00763216
plate	10	E03	hsa-miR-1293	MIMAT0005883	UGGGUGGUCUGGAGAUUUGUGC	30.94059591
plate	10	E04	hsa-miR-614	MIMAT0003282	GAACGCCUGUUCUUGCCAGGUGG	-36.81587092
plate	10	E05	hsa-miR-628-3p	MIMAT0003297	UCUAGUAAGAGUGGCAGUCGA	29.53548211
plate	10	E06	hsa-miR-508-5p	MIMAT0004778	UACUCCAGAGGGCGUCACUCAUG	-13.41222389
plate	10	E07	hsa-miR-513b-5p	MIMAT0005788	UUCACAAGGAGGUGUCAUUUAU	-5.281358376
plate	10	E08	hsa-miR-412-3p	MIMAT0002170	ACUUCACCUGGUCCACUAGCCGU	-47.45714676
plate	10	E09	hsa-miR-938	MIMAT0004981	UGCCCUUAAAGGUGAACCCAGU	18.18046216
plate	10	E10	hsa-miR-501-3p	MIMAT0004774	AAUGCACCCGGGCAAGGAUUCU	-67.40394536
plate	10	E11	hsa-miR-548b-3p	MIMAT0003254	CAAGAACCUCAGUUGCUUUUGU	20.47160313
plate	10	E12				
plate	10	F01				
plate	10	F02	hsa-miR-514a-3p	MIMAT0002883	AUUGACACUUCUGUGAGUAGA	22.99096322
plate	10	F03	hsa-miR-101-5p	MIMAT0004513	CAGUUAUCACAGUGCUGAUGCU	32.10854081
plate	10	F04	hsa-miR-365a-5p	MIMAT0009199	AGGGACUUUUGGGGGCAGAUGUG	16.42183247

plate 10	F05	hsa-miR-1247-5p	MIMAT0005899	ACCCGUCCCGUUCGUCCCCGGA	3.225397457
plate 10	F06	hsa-miR-1226-3p	MIMAT0005577	UCACCAGCCCUGUGUUCCCUAG	-47.12824274
plate 10	F07	hsa-miR-633	MIMAT0003303	CUAAUAGUAUCUACCACAAUAAA	-2.641176397
plate 10	F08	hsa-miR-575	MIMAT0003240	GAGCCAGUUGGACAGGAGC	17.76429788
plate 10	F09	hsa-miR-890	MIMAT0004912	UACUUGGAAAGGCAUCAGUUG	20.93251625
plate 10	F10	hsa-miR-1471	MIMAT0007349	GCCCGCGUGUGGAGCCAGGUGU	15.22927569
plate 10	F11	hsa-miR-526b-5p	MIMAT0002835	CUCUUGAGGGAAGCACUUUCUGU	-67.07727877
plate 10	F12				
plate 10	G01				
plate 10	G02	hsa-miR-1255b-5p	MIMAT0005945	CGGAUGAGCAAAGAAAGUGGUU	17.62110157
plate 10	G03	hsa-miR-3196	MIMAT0015080	CGGGGCGGCAGGGGCCUC	26.13456973
plate 10	G04	hsa-miR-452-5p	MIMAT0001635	AACUGUUUGCAGAGGAAACUGA	34.43548086
plate 10	G05	hsa-miR-1226-5p	MIMAT0005576	GUGAGGGCAUGCAGGCCUGGAUGGGG	9.812427749
plate 10	G06	hsa-miR-548d-3p	MIMAT0003323	CAAAAACCACAGUUUCUUUUGC	44.42118609
plate 10	G07	hsa-miR-1284	MIMAT0005941	UCUAUACAGACCCUGGCUUUUC	-50.37477159
plate 10	G08	hsa-miR-520d-3p	MIMAT0002856	AAAGUGCUUCUCUUUGGUGGGU	38.44274012
plate 10	G09	hsa-miR-1184	MIMAT0005829	CCUGCAGCGACUUGAUGGCUUCC	-74.77408047
plate 10	G10	hsa-miR-613	MIMAT0003281	AGGAAUGUUCCUUCUUUGCC	24.3423784
plate 10	G11	hsa-miR-198	MIMAT0000228	GGUCCAGAGGGGAGAUAGGUUC	10.49708511
plate 10	G12				
plate 10	H01				
plate 10	H02				
plate 10	H03				
plate 10	H04				
plate 10	H05				
plate 10	H06				
plate 10	H07				
plate 10	H08				
plate 10	H09				
plate 10	H10				
plate 10	H11				
plate 10	H12				
plate 11	A01				
plate 11	A02				
plate 11	A03				
plate 11	A04				
plate 11	A05				
plate 11	A06				
plate 11	A07				
plate 11	A08				
plate 11	A09				
plate 11	A10				
plate 11	A11				
plate 11	A12				
plate 11	B01				
plate 11	B02				
plate 11	B03				
plate 11	B04	hsa-miR-933	MIMAT0004976	UGUGCGCAGGGAGACCUCUCCC	9.231561253
plate 11	B05	hsa-miR-431-3p	MIMAT0004757	CAGGUCGUCUUGCAGGGCUUCU	21.71150979

plate 11	B06	hsa-miR-138-1-3p	MIMAT0004607	GCUACUUCACAACACCAGGGCC	-47.98140195
plate 11	B07	hsa-miR-617	MIMAT0003286	AGACUUCCCAUUUGAAGGUGGC	33.32851222
plate 11	B08	hsa-miR-523-3p	MIMAT0002840	GAACGCGCUUCCCUAUAGAGGGU	-34.73681762
plate 11	B09	hsa-miR-1273c	MIMAT0015017	GGCGACAAAACGAGACCCUGUC	-83.26206173
plate 11	B10	hsa-miR-146a-3p	MIMAT0004608	CCUCUGAAAUUCAGUUCUUCAG	3.260119793
plate 11	B11	hsa-miR-548p	MIMAT0005934	UAGCAAAAACUGCAGUUACUUU	6.589926631
plate 11	B12				
plate 11	C01				
plate 11	C02	hsa-miR-125b-1-3p	MIMAT0004592	ACGGGUUAGGCUCUUGGGAGCU	-3.645269676
plate 11	C03	hsa-let-7a-3p	MIMAT0004481	CUAUACAAUCUACUGUCUUUC	-31.33965001
plate 11	C04	hsa-miR-891a-5p	MIMAT0004902	UGCAACGAACCUGAGCCACUGA	-60.46499162
plate 11	C05	hsa-miR-644a	MIMAT0003314	AGUGUGGCUUUCUUAGAGC	21.7442799
plate 11	C06	hsa-miR-186-3p	MIMAT0004612	GCCCAAAGGUGAAUUUUUUGGG	15.71640103
plate 11	C07	hsa-miR-611	MIMAT0003279	GCGAGGACCCCUCGGGGUCUGAC	17.90653642
plate 11	C08	hsa-miR-3179	MIMAT0015056	AGAAGGGGUGAAAUUUAAACGU	19.153621
plate 11	C09	hsa-miR-4286	MIMAT0016916	ACCCCACUCCUGGUACC	-48.68049754
plate 11	C10	hsa-miR-4324	MIMAT0016876	CCCUGAGACCCUAACCUUAA	-58.10190289
plate 11	C11	hsa-miR-877-5p	MIMAT0004949	GUAGAGGAGAUGGCGCAGGG	-17.49063928
plate 11	C12				
plate 11	D01				
plate 11	D02	hsa-miR-1249	MIMAT0005901	ACGCCCUUCCCCCCCUUCUUCA	-62.97008413
plate 11	D03	hsa-miR-1245a	MIMAT0005897	AAGUGAUCUAAAGGCCUACAU	15.05917836
plate 11	D04	hsa-miR-518f-5p	MIMAT0002841	CUCUAGAGGGAAGCACUUUCUC	-31.06292467
plate 11	D05	hsa-miR-1206	MIMAT0005870	UGUUCAUGUAGAUGUUUAAGC	16.4118555
plate 11	D06	hsa-miR-558	MIMAT0003222	UGAGCUGCUGUACCAAAAU	10.02350547
plate 11	D07	hsa-miR-640	MIMAT0003310	AUGAUCCAGGAACCUGCCUCU	36.17404972
plate 11	D08	hsa-miR-1254	MIMAT0005905	AGCCUGGAAGCUGGAGCCUGCAGU	-5.593270396
plate 11	D09	hsa-miR-221-5p	MIMAT0004568	ACCUGGCAUACAAUGUAGAUUU	43.39803743
plate 11	D10	hsa-miR-1225-5p	MIMAT0005572	GUGGGUACGGCCCAGUGGGGGG	18.34711229
plate 11	D11	hsa-miR-629-3p	MIMAT0003298	GUUCUCCCAACGUAAGCCCAGC	-16.15980888
plate 11	D12				
plate 11	E01				
plate 11	E02	hsa-miR-3065-5p	MIMAT0015066	UCAACAAAAUCACUGAUGCUGGA	26.61974338
plate 11	E03	hsa-miR-466	MIMAT0015002	AUACACAUACACGCAACACACAU	5.084322336
plate 11	E04	hsa-miR-3151-5p	MIMAT0015024	GGUGGGGCAAUGGGAUCAGGU	6.551694842
plate 11	E05	hsa-miR-623	MIMAT0003292	AUCCCUUGCAGGGGCUGUUGGGU	-9.334524115
plate 11	E06	hsa-miR-4281	MIMAT0016907	GGGUCCCGGGGAGGGGGG	24.93572407
plate 11	E07	hsa-miR-551a	MIMAT0003214	GCGACCCACUCUUGGUUUCCA	23.59214974
plate 11	E08	hsa-miR-708-3p	MIMAT0004927	CAACUAGACUGUGAGCUUCUAG	48.76869363
plate 11	E09	hsa-miR-562	MIMAT0003226	AAAGUAGCUGUACCAUUUGC	28.40935526
plate 11	E10	hsa-miR-147b	MIMAT0004928	GUGUGCGGAAAUGCUUCUGCUA	7.041425864
plate 11	E11	hsa-miR-1250-5p	MIMAT0005902	ACGGUGCUGGAUGUGGCCUUU	-38.09211232
plate 11	E12				
plate 11	F01				
plate 11	F02	hsa-miR-130b-5p	MIMAT0004680	ACUCUUUCCCUGUUGCACUAC	-28.16094977
plate 11	F03	hsa-miR-15a-3p	MIMAT0004488	CAGGCCAUAUUGUGCUGCCUCA	-4.892354249
plate 11	F04	hsa-miR-4291	MIMAT0016922	UUCAGCAGGAACAGCU	17.94840934
plate 11	F05	hsa-miR-1301-3p	MIMAT0005797	UUGCAGCUGCCUGGGAGUGACUUC	25.92428892
plate 11	F06	hsa-miR-1537-3p	MIMAT0007399	AAAACCGUCUAGUUACAGUUGU	33.81824324

plate	11	F07	hsa-miR-1269a	MIMAT0005923	CUGGACUGAGCCGUGCUACUGG	-6.847637215
plate	11	F08	hsa-miR-3202	MIMAT0015089	UGGAAGGGAGAAGAGCUUUAAU	68.76027859
plate	11	F09	hsa-miR-593-5p	MIMAT0003261	AGGCACCAGCCAGGCAUUGCUCAGC	-88.05742051
plate	11	F10	hsa-miR-1238-3p	MIMAT0005593	CUUCCUCGUCUGUCUGCCCC	11.64926682
plate	11	F11	hsa-miR-541-3p	MIMAT0004920	UGGUGGGCACAGAAUCUGGACU	-80.30182886
plate	11	F12				
plate	11	G01				
plate	11	G02	hsa-miR-548j-5p	MIMAT0005875	AAAAGUAAUUGCGGUCUUUGGU	8.080966435
plate	11	G03	hsa-miR-548n	MIMAT0005916	CAAAAGUAAUUGUGGAUUUUGU	8.607108685
plate	11	G04	hsa-miR-548j-5p	MIMAT0005875	AAAAGUAAUUGCGGUCUUUGGU	22.66548398
plate	11	G05	hsa-miR-548i	MIMAT0005935	AAAAGUAAUUGCGGAUUUUGCC	14.23264348
plate	11	G06	hsa-miR-892b	MIMAT0004918	CACUGGCUCCUUUCUGGGUAGA	21.83166685
plate	11	G07	hsa-miR-651-5p	MIMAT0003321	UUUAGGAUAAGCUUGACUUUUG	32.90432141
plate	11	G08	hsa-miR-889-3p	MIMAT0004921	UUAAUAUCGGACAACCAUUGU	47.41601649
plate	11	G09	hsa-miR-26a-1-3p	MIMAT0004499	CCUAUUCUUGGUUACUUGCACG	28.36566178
plate	11	G10	hsa-miR-1261	MIMAT0005913	AUGGAUAAGGCUUUGGCUU	28.42027863
plate	11	G11	hsa-let-7f-2-3p	MIMAT0004487	CUAUACAGUCUACUGUCUUUCC	18.72578906
plate	11	G12				
plate	11	H01				
plate	11	H02				
plate	11	H03				
plate	11	H04				
plate	11	H05				
plate	11	H06				
plate	11	H07				
plate	11	H08				
plate	11	H09				
plate	11	H10				
plate	11	H11				
plate	11	H12				
plate	12	A01				
plate	12	A02				
plate	12	A03				
plate	12	A04				
plate	12	A05				
plate	12	A06				
plate	12	A07				
plate	12	A08				
plate	12	A09				
plate	12	A10				
plate	12	A11				
plate	12	A12				
plate	12	B01				
plate	12	B02				
plate	12	B03				
plate	12	B04	hsa-miR-1260a	MIMAT0005911	AUCCCACCUCUGCCACCA	-156.9897352
plate	12	B05	hsa-miR-599	MIMAT0003267	GUUGUGUCAGUUUAUCAAAC	34.77241967
plate	12	B06	hsa-miR-1302	MIMAT0005890	UUGGGACAUACUUAUGCUAAA	-45.33876327
plate	12	B07	hsa-miR-589-3p	MIMAT0003256	UCAGAACAAAUGCCGGUUCCCAGA	2.479370698

plate	12	B08	hsa-miR-1262	MIMAT0005914	AUGGGUGAAUUUGUAGAAGGAU	12.0219855
plate	12	B09	hsa-miR-3148	MIMAT0015021	UGGAAAAAACUGGUGUGUGCUU	14.85463954
plate	12	B10	hsa-miR-891b	MIMAT0004913	UGCAACUUACCUGAGUCAUUGA	-14.64936518
plate	12	B11	hsa-miR-892a	MIMAT0004907	CACUGUGUCCUUUCUGCGUAG	17.68927585
plate	12	B12				
plate	12	C01				
plate	12	C02	hsa-miR-525-5p	MIMAT0002838	CUCCAGAGGGAUGCACUUUCU	-35.47105731
plate	12	C03	hsa-miR-648	MIMAT0003318	AAGUGUGCAGGGCACUGGU	24.02458887
plate	12	C04	hsa-miR-330-5p	MIMAT0004693	UCUCUGGGCCUGUGUCUUAGGC	11.76032677
plate	12	C05	hsa-miR-194-3p	MIMAT0004671	CCAGUGGGGCUGCUGUUAUCUG	38.31472392
plate	12	C06	hsa-miR-1237-3p	MIMAT0005592	UCCUUCUGCUCCGUCCCCCAG	17.66152416
plate	12	C07	hsa-miR-1827	MIMAT0006767	UGAGGCAGUAGAUUGAAU	0.808322862
plate	12	C08	hsa-let-7a-2-3p	MIMAT0010195	CUGUACAGCCUCCUAGCUUUCC	-147.9010586
plate	12	C09	hsa-miR-621	MIMAT0003290	GGCUAGCAACAGCGCUUACCU	-103.8393133
plate	12	C10	hsa-miR-548a-5p	MIMAT0004803	AAAAGUAAUUGCGAGUUUUACC	13.85954344
plate	12	C11	hsa-miR-668-3p	MIMAT0003881	UGUCACUCGGCUCGGCCCACUAC	9.863300931
plate	12	C12				
plate	12	D01				
plate	12	D02	hsa-let-7f-1-3p	MIMAT0004486	CUAUACAAUCUAUUGCCUUCCC	29.54915627
plate	12	D03	hsa-miR-24-2-5p	MIMAT0004497	UGCCUACUGAGCUGAAACACAG	31.63846163
plate	12	D04	hsa-miR-583	MIMAT0003248	CAAAGAGGAAGGUCCCAUUAC	18.86079337
plate	12	D05	hsa-miR-218-1-3p	MIMAT0004565	AUGGUUCCGUCAAGCACCAUGG	-106.9296616
plate	12	D06	hsa-miR-19a-5p	MIMAT0004490	AGUUUUGCAUAGUUGCACUACA	37.80726455
plate	12	D07	hsa-miR-4317	MIMAT0016872	ACAUUGCCAGGGAGUUU	32.88332289
plate	12	D08	hsa-miR-656-3p	MIMAT0003332	AAUAUUAUACAGUCAACCUCU	37.14915319
plate	12	D09	hsa-miR-654-3p	MIMAT0004814	UAUGUCUGCUGACCAUCACCUU	14.47602728
plate	12	D10	hsa-miR-576-3p	MIMAT0004796	AAGAUGUGGAAAAAUUGGAAUC	5.878951999
plate	12	D11	hsa-miR-1268a	MIMAT0005922	CGGGCGUGGUGGUGGGGG	10.87029061
plate	12	D12				
plate	12	E01				
plate	12	E02	hsa-miR-519b-3p	MIMAT0002837	AAAGUGCAUCCUUUUAGAGGUU	36.50491766
plate	12	E03	hsa-miR-27b-5p	MIMAT0004588	AGAGCUUAGCUGAUUGGUGAAC	9.968360878
plate	12	E04	hsa-miR-671-3p	MIMAT0004819	UCCGGUUCUCAGGGCUCCACC	-84.7025449
plate	12	E05	hsa-miR-586	MIMAT0003252	UAUGCAUUGUAUUUUUAGGUCC	35.94591946
plate	12	E06	hsa-miR-135a-3p	MIMAT0004595	UAUAGGGAUUGGAGCCGUGGCG	35.67236714
plate	12	E07	hsa-miR-1304-5p	MIMAT0005892	UUUGAGGCUACAGUGAGAUGUG	-17.64852933
plate	12	E08	hsa-miR-500a-3p	MIMAT0002871	AUGCACCUGGGCAAGGAUUCUG	-6.700489949
plate	12	E09	hsa-miR-624-3p	MIMAT0004807	CACAAGGUAUUGGUAUUACCU	-68.52529533
plate	12	E10	hsa-miR-520a-3p	MIMAT0002834	AAAGUGCUUCCCUUUGGACUGU	2.005609805
plate	12	E11	hsa-miR-1243	MIMAT0005894	AACUGGAUCAAUUAUAGGAGUG	18.58129426
plate	12	E12				
plate	12	F01				
plate	12	F02	hsa-miR-181b-3p	MIMAT0022692	CUCACUGAACAAUGAAUGCAA	23.99683719
plate	12	F03	hsa-miR-509-3-5p	MIMAT0004975	UACUGCAGACGUGGCAAUCAUG	41.92838964
plate	12	F04	hsa-miR-610	MIMAT0003278	UGAGCUAAAUGUGUGCUGGGA	17.50294311
plate	12	F05	hsa-miR-520d-5p	MIMAT0002855	CUACAAAGGGAAGCCCUUUC	-39.12436829
plate	12	F06	hsa-miR-507	MIMAT0002879	UUUUGCACCUUUUGGAGUGAA	-2.418801546
plate	12	F07	hsa-miR-616-5p	MIMAT0003284	ACUCAAAACCCUUCAGUGACUU	-9.521250412
plate	12	F08	hsa-miR-888-3p	MIMAT0004917	GACUGACACCUCUUUGGGUGAA	30.69688664

plate 12	F09	hsa-miR-567	MIMAT0003231	AGUAUGUUCUUCCAGGACAGAAC	24.67477119
plate 12	F10	hsa-miR-202-5p	MIMAT0002810	UUCCUAUGCAUAUACUUCUUUG	-25.04237087
plate 12	F11	hsa-miR-337-5p	MIMAT0004695	GAACGGCUUCAUACAGGAGUU	9.205189565
plate 12	F12				
plate 12	G01				
plate 12	G02	hsa-miR-7-2-3p	MIMAT0004554	CAACAAAUCCCAGUCUACCUAA	38.90940287
plate 12	G03	hsa-miR-196b-3p	MIMAT0009201	UCGACAGCACGACACUGCCUUC	48.74539261
plate 12	G04	hsa-miR-218-2-3p	MIMAT0004566	CAUGGUUCUGUCAAGCACCGCG	-62.62013341
plate 12	G05	hsa-miR-2115-3p	MIMAT0011159	CAUCAGAAUUCAUGGAGGCUAG	55.90136259
plate 12	G06	hsa-miR-3120-3p	MIMAT0014982	CACAGCAAGUGUAGACAGGCA	-51.56306956
plate 12	G07	hsa-miR-1266-5p	MIMAT0005920	CCUCAGGGCUGUAGAACAGGGCU	-38.93208877
plate 12	G08	hsa-miR-501-5p	MIMAT0002872	AAUCCUUUGUCCCUGGGUGAGA	34.68520009
plate 12	G09	hsa-miR-513c-5p	MIMAT0005789	UUCUCAAGGAGGUGUCGUUUAU	16.41468064
plate 12	G10	hsa-miR-559	MIMAT0003223	UAAAGUAAAUAUGCACCAAAA	15.83387754
plate 12	G11	hsa-miR-3142	MIMAT0015011	AAGGCCUUUCUGAACCUUCAGA	36.67935682
plate 12	G12				
plate 12	H01				
plate 12	H02				
plate 12	H03				
plate 12	H04				
plate 12	H05				
plate 12	H06				
plate 12	H07				
plate 12	H08				
plate 12	H09				
plate 12	H10				
plate 12	H11				
plate 12	H12				
plate 13	A01				
plate 13	A02				
plate 13	A03				
plate 13	A04				
plate 13	A05				
plate 13	A06				
plate 13	A07				
plate 13	A08				
plate 13	A09				
plate 13	A10				
plate 13	A11				
plate 13	A12				
plate 13	B01				
plate 13	B02				
plate 13	B03				
plate 13	B04	hsa-miR-377-5p	MIMAT0004689	AGAGGUUGCCCUUGGUGAAUUC	6.273073977
plate 13	B05	hsa-miR-645	MIMAT0003315	UCUAGGCUGGUACUGCUGA	2.35062489
plate 13	B06	hsa-miR-33a-3p	MIMAT0004506	CAAUGUUUCCACAGUGCAUCAC	12.5215628
plate 13	B07	hsa-miR-1972	MIMAT0009447	UCAGGCCAGGCACAGUGGCUCA	-45.80680948
plate 13	B08	hsa-miR-4287	MIMAT0016917	UCUCCCUUGAGGGCACUUU	-66.38458084
plate 13	B09	hsa-miR-941	MIMAT0004984	CACCCGGCUGUGUGCACAUGUGC	16.24651795

plate	13	B10	hsa-miR-212-5p	MIMAT0022695	ACCUUGGCUCUAGACUGCUUACU	-34.07969246
plate	13	B11	hsa-miR-630	MIMAT0003299	AGUAUUCUGUACCAGGGAAGGU	2.211647672
plate	13	B12				
plate	13	C01				
plate	13	C02	hsa-miR-376a-2-5p	MIMAT0022928	GGUAGAUUUUCCUUCUAUGGU	6.735721818
plate	13	C03	hsa-miR-1248	MIMAT0005900	ACCUUCUUGUAUAAGCACUGUGCUAAA	3.811714316
plate	13	C04	hsa-miR-591	MIMAT0003259	AGACCAUGGGUUCUCAUUGU	16.12399856
plate	13	C05	hsa-miR-1539	MIMAT0007401	UCCUGCGCGUCCCAGAUGCCC	-62.23903675
plate	13	C06	hsa-miR-620	MIMAT0003289	AUGGAGAUAGAUAUAGAAAU	-3.464474457
plate	13	C07	hsa-miR-935	MIMAT0004978	CCAGUUACCGCUUCCGCUACCGC	12.77208752
plate	13	C08	hsa-miR-380-5p	MIMAT0000734	UGGUUGACCAUAGAACAUGCGC	5.441039322
plate	13	C09	hsa-miR-106a-3p	MIMAT0004517	CUGCAAUGUAAGCACUUCUUAC	2.809615435
plate	13	C10	hsa-miR-675-3p	MIMAT0006790	CUGUAUGCCCUCACCGCUCA	-62.71631377
plate	13	C11	hsa-miR-888-5p	MIMAT0004916	UACUCAAAAAGCUGUCAGUCA	-12.64062808
plate	13	C12				
plate	13	D01				
plate	13	D02	hsa-miR-764	MIMAT0010367	GCAGGUGCUCACUUGUCCUCCU	-21.59185805
plate	13	D03	hsa-miR-1181	MIMAT0005826	CCGUCGCCGCCACCCGAGCCG	-6.915132465
plate	13	D04	hsa-miR-877-3p	MIMAT0004950	UCCUCUUCUCCCUCCUCCCAG	-24.95474097
plate	13	D05	hsa-miR-936	MIMAT0004979	ACAGUAGAGGGAGGAAUCGCAG	-18.37892423
plate	13	D06	hsa-miR-100-3p	MIMAT0004512	CAAGCUUGUAUCUAUAGGUAUG	21.90252496
plate	13	D07	hsa-miR-192-3p	MIMAT0004543	CUGCCAAUUCCAUAGGUCACAG	9.039817785
plate	13	D08	hsa-miR-335-3p	MIMAT0004703	UUUUUCAUUAUUGCUCCUGACC	-13.33002822
plate	13	D09	hsa-miR-658	MIMAT0003336	GGCGGAGGGAAGUAGGUCCGUUGGU	2.838873797
plate	13	D10	hsa-miR-105-3p	MIMAT0004516	ACGGAUGUUUGAGCAUGUGCUA	3.155229831
plate	13	D11	hsa-miR-320e	MIMAT0015072	AAAGCUGGGUUGAGAAGG	7.723191518
plate	13	D12				
plate	13	E01				
plate	13	E02	hsa-miR-450a-1-3p	MIMAT0022700	AUUGGGAACAUUUUGCAUGUAU	-13.56409511
plate	13	E03	hsa-miR-577	MIMAT0003242	UAGAUAAAAUAUUGGUACCUG	7.033791376
plate	13	E04	hsa-miR-627-5p	MIMAT0003296	GUGAGUCUCUAAGAAAAGAGGA	1.829460325
plate	13	E05	hsa-miR-549a	MIMAT0003333	UGACAACUAUGGAUGAGCUCU	11.60358171
plate	13	E06	hsa-miR-302a-5p	MIMAT0000683	ACUUAAACGUGGAUGUACUUGCU	-12.29135639
plate	13	E07	hsa-miR-450b-3p	MIMAT0004910	UUGGGAUCAUUUUGCAUCCAUA	-4.536061946
plate	13	E08	hsa-miR-876-3p	MIMAT0004925	UGGUGGUUUACAAAGUAAUUCA	8.696032038
plate	13	E09	hsa-miR-1252-5p	MIMAT0005944	AGAAGGAAAUUGAAUUCAUUUA	1.215034734
plate	13	E10	hsa-miR-365b-5p	MIMAT0022833	AGGGACUUUCAGGGGCAGCUGU	-28.93570678
plate	13	E11	hsa-miR-940	MIMAT0004983	AAGGCAGGGCCCCCGCUCCCC	-18.0552536
plate	13	E12				
plate	13	F01				
plate	13	F02	hsa-miR-33b-3p	MIMAT0004811	CAGUGCCUCGGCAGUGCAGCCC	-2.295968645
plate	13	F03	hsa-miR-767-3p	MIMAT0003883	UCUGCUCAUACCCCAUGGUUUCU	-43.14429858
plate	13	F04	hsa-miR-922	MIMAT0004972	GCAGCAGAGAAUAGGACUACGUC	-12.82715013
plate	13	F05	hsa-miR-934	MIMAT0004977	UGUCUACUACUGGAGACACUGG	-1.4182178
plate	13	F06	hsa-miR-26b-3p	MIMAT0004500	CCUGUUCUCCAUUACUUGGCUC	-51.28726631
plate	13	F07	hsa-miR-103a-2-5p	MIMAT0009196	AGCUUCUUUACAGUGCUGCCUUG	-17.66575167
plate	13	F08	hsa-miR-148a-5p	MIMAT0004549	AAAGUUCUGAGACACUCCGACU	5.172228126
plate	13	F09	hsa-miR-191-3p	MIMAT0001618	GCUGCGCUUGGAUUUCGUCCCC	-16.06568502
plate	13	F10	hsa-miR-374b-3p	MIMAT0004956	CUUAGCAGGUUGUAUUAUCAUU	0.033728389

plate	13	F11	hsa-miR-411-3p	MIMAT0004813	UAUGUAACACGGUCCACUAACC	14.65193725
plate	13	F12				
plate	13	G01				
plate	13	G02	hsa-miR-505-5p	MIMAT0004776	GGGAGCCAGGAAGUAUUGAUGU	-4.985909254
plate	13	G03	hsa-miR-548l	MIMAT0005889	AAAAGUAUUUGCGGGUUUUGUC	-4.600064612
plate	13	G04	hsa-miR-1292-5p	MIMAT0005943	UGGGAACGGGUUCCGGCAGACGCUG	9.721903338
plate	13	G05	hsa-miR-1307-3p	MIMAT0005951	ACUCGGCGUGGCGUCGGUCGUG	-10.48282392
plate	13	G06	hsa-miR-30d-3p	MIMAT0004551	CUUUCAGUCAGAUGUUUGCUGC	-2.080188229
plate	13	G07	hsa-miR-449c-5p	MIMAT0010251	UAGGCAGUGUAUUGCUAGCGGCUGU	-49.52445003
plate	13	G08	hsa-miR-1180-3p	MIMAT0005825	UUUCCGGCUCGCGUGGGUGUGU	-4.927392531
plate	13	G09	hsa-miR-1273f	MIMAT0020601	GGAGAUGGAGGUUGCAGUG	-33.5932722
plate	13	G10	hsa-miR-1273e	MIMAT0018079	UUGCUUGAACCCAGGAAGUGGA	-68.42535156
plate	13	G11	hsa-miR-1285-5p	MIMAT0022719	GAUCUCACUUUGUUGCCCAGG	5.660477033
plate	13	G12				
plate	13	H01				
plate	13	H02				
plate	13	H03				
plate	13	H04				
plate	13	H05				
plate	13	H06				
plate	13	H07				
plate	13	H08				
plate	13	H09				
plate	13	H10				
plate	13	H11				
plate	13	H12				
plate	14	A01				
plate	14	A02				
plate	14	A03				
plate	14	A04				
plate	14	A05				
plate	14	A06				
plate	14	A07				
plate	14	A08				
plate	14	A09				
plate	14	A10				
plate	14	A11				
plate	14	A12				
plate	14	B01				
plate	14	B02				
plate	14	B03				
plate	14	B04	hsa-miR-2355-5p	MIMAT0016895	AUCCCCAGAUACAAUGGACAA	-81.85919407
plate	14	B05	hsa-miR-3130-3p	MIMAT0014994	GCUGCACCGGAGACUGGGUAA	8.119499768
plate	14	B06	hsa-miR-4306	MIMAT0016858	UGGAGAGAAAGGCAGUA	14.44835572
plate	14	B07	hsa-miR-1273d	MIMAT0015090	GAACCCAUGAGGUUGAGGCUGCAGU	-30.66141732
plate	14	B08	hsa-miR-1289	MIMAT0005879	UGGAGUCCAGGAAUCUGCAUUUU	-71.50625289
plate	14	B09	hsa-miR-518a-5p	MIMAT0005457	CUGCAAAGGGAAGCCCUUUC	3.217230199
plate	14	B10	hsa-miR-544b	MIMAT0015004	ACCUGAGGUUGUGCAUUUCUAA	-2.079666512
plate	14	B11	hsa-miR-3130-5p	MIMAT0014995	UACCCAGUCUCCGGUGCAGCC	-72.28439092

plate	14	B12				
plate	14	C01				
plate	14	C02	hsa-miR-1273a	MIMAT0005926	GGGCGACAAAGCAAGACUCUUUCUU	-32.83835109
plate	14	C03	hsa-miR-499b-5p	MIMAT0019897	ACAGACUUGCUGUGAUGUUCA	-11.18573414
plate	14	C04	hsa-miR-518f-3p	MIMAT0002842	GAAAGCGCUUCUCUUUAGAGG	11.91940713
plate	14	C05	hsa-miR-122-3p	MIMAT0004590	AACGCCAUUAUCACACUAAAUA	6.298286244
plate	14	C06	hsa-miR-1272	MIMAT0005925	GAUGAUGAUGGCAGCAAAUUCUGAAA	-74.52431681
plate	14	C07	hsa-miR-323b-3p	MIMAT0015050	CCCAAUACACGGUCGACCUCUU	13.14219546
plate	14	C08	hsa-miR-1234-3p	MIMAT0005589	UCGGCCUGACCACCCACCCCAC	0.000926355
plate	14	C09	hsa-miR-1197	MIMAT0005955	UAGGACACAUGGUCUACUUCU	21.11625753
plate	14	C10	hsa-miR-556-3p	MIMAT0004793	AUAUUACCAUUAGCUCAUCUUU	-38.5224641
plate	14	C11	hsa-miR-18b-3p	MIMAT0004751	UGCCCUAAAUGCCCCUUCUGGC	-10.23529412
plate	14	C12				
plate	14	D01				
plate	14	D02	hsa-miR-130a-5p	MIMAT0004593	UUCACAUUGUGCUACUGUCUGC	7.070866142
plate	14	D03	hsa-miR-138-2-3p	MIMAT0004596	GCUAUUUCACGACACCAGGGUU	-1.938860584
plate	14	D04	hsa-miR-379-3p	MIMAT0004690	UAUGUAACAUGGUCCACUAACU	-33.81843446
plate	14	D05	hsa-miR-548k	MIMAT0005882	AAAAGUACUUGCGGAUUUUGCU	9.546086151
plate	14	D06	hsa-miR-548h-5p	MIMAT0005928	AAAAGUAAUCGCGGUUUUUGUC	23.77119037
plate	14	D07	hsa-miR-548k	MIMAT0005882	AAAAGUACUUGCGGAUUUUGCU	23.40435387
plate	14	D08	hsa-miR-550a-3p	MIMAT0003257	UGUCUUACUCCCUCAGGCACAU	-51.56924502
plate	14	D09	hsa-miR-876-5p	MIMAT0004924	UGGAUUUCUUUGUGAAUCACCA	12.41408059
plate	14	D10	hsa-miR-1255a	MIMAT0005906	AGGAUGAGCAAAGAAAGUAGAUU	7.760074108
plate	14	D11	hsa-miR-1278	MIMAT0005936	UAGUACUGUGCAUAUCAUCUAU	14.2890227
plate	14	D12				
plate	14	E01				
plate	14	E02	hsa-miR-1306-3p	MIMAT0005950	ACGUUGGCUCUGGUGGUG	-7.070866142
plate	14	E03	hsa-miR-718	MIMAT0012735	CUUCCGCCCCGCCGGGCGUCG	-4.780917091
plate	14	E04	hsa-miR-2909	MIMAT0013863	GUUAGGGCCAACAUCUCUUGG	-12.49930523
plate	14	E05	hsa-miR-4319	MIMAT0016870	UCCCUGAGCAAAGCCAC	-43.63779528
plate	14	E06	hsa-miR-302e	MIMAT0005931	UAAGUGCUUCCAUGCUU	21.21074572
plate	14	E07	hsa-miR-1469	MIMAT0007347	CUCGGCGCGGGGCGCGGGCUCC	27.43955535
plate	14	E08	hsa-miR-1305	MIMAT0005893	UUUUCAACUCUAAUGGGAGAGA	-41.15145901
plate	14	E09	hsa-miR-1207-3p	MIMAT0005872	UCAGCUGGCCCUCAUUUC	-7.34877258
plate	14	E10	hsa-miR-3163	MIMAT0015037	UAUAAAAUGAGGGCAGUAAGAC	-4.410375174
plate	14	E11	hsa-miR-2861	MIMAT0013802	GGGGCCUGGCGGUGGGCGG	9.566465956
plate	14	E12				
plate	14	F01				
plate	14	F02	hsa-miR-551b-5p	MIMAT0004794	GAAAUCAAGCGUGGGUGAGACC	-3.695229273
plate	14	F03	hsa-miR-224-3p	MIMAT0009198	AAAAUGGUGCCCUAGUGACUACA	-41.94812413
plate	14	F04	hsa-miR-600	MIMAT0003268	ACUUACAGACAAGAGCCUUGCUC	-79.22278833
plate	14	F05	hsa-miR-187-5p	MIMAT0004561	GGCUACAACACAGGACCCGGGC	-55.17091246
plate	14	F06	hsa-miR-1205	MIMAT0005869	UCUGCAGGGUUUGCUUUGAG	18.59657249
plate	14	F07	hsa-miR-1229-3p	MIMAT0005584	CUCUCACCACUGCCCUCCCACAG	-76.25289486
plate	14	F08	hsa-miR-604	MIMAT0003272	AGGCUGCGGAAUUCAGGAC	7.97869384
plate	14	F09	hsa-miR-449b-3p	MIMAT0009203	CAGCCACAACUACCCUGCCACU	-12.86428902
plate	14	F10	hsa-let-7g-3p	MIMAT0004584	CUGUACAGGCCACUGCCUUGC	-79.42658638
plate	14	F11	hsa-let-7c-3p	MIMAT0026472	CUGUACAACCUUCUAGCUUUCC	-52.3770264
plate	14	F12				

plate	14	G01				
plate	14	G02	hsa-miR-23c	MIMAT0018000	AUCACAUUGCCAGUGAUUACCC	-37.20333488
plate	14	G03	hsa-miR-98-3p	MIMAT0022842	CUAUACAACUUACUACUUUCCC	-11.02640111
plate	14	G04	hsa-miR-133a-5p	MIMAT0026478	AGCUGGUAAAAUGGAACCAAAU	14.01667439
plate	14	G05	hsa-miR-136-3p	MIMAT0004606	CAUCAUCGUCUCAAAUGAGUCU	7.93052339
plate	14	G06	hsa-miR-190a-3p	MIMAT0026482	CUAUAUAUCAAACAUAUUCCU	-4.421491431
plate	14	G07	hsa-miR-197-5p	MIMAT0022691	CGGGUAGAGAGGGCAGUGGGAGG	-3.75266327
plate	14	G08	hsa-miR-204-3p	MIMAT0022693	GCUGGGAAGGCAAAGGGACGU	-0.276980083
plate	14	G09	hsa-miR-211-3p	MIMAT0022694	GCAGGGACAGCAAAGGGGUGC	11.15238536
plate	14	G10	hsa-miR-301a-5p	MIMAT0022696	GCUCUGACUUUAUUGCACUACU	9.753589625
plate	14	G11	hsa-miR-345-3p	MIMAT0022698	GCCCUGAACGAGGGGUCUGGAG	12.23436776
plate	14	G12				
plate	14	H01				
plate	14	H02				
plate	14	H03				
plate	14	H04				
plate	14	H05				
plate	14	H06				
plate	14	H07				
plate	14	H08				
plate	14	H09				
plate	14	H10				
plate	14	H11				
plate	14	H12				
plate	15	A01				
plate	15	A02				
plate	15	A03				
plate	15	A04				
plate	15	A05				
plate	15	A06				
plate	15	A07				
plate	15	A08				
plate	15	A09				
plate	15	A10				
plate	15	A11				
plate	15	A12				
plate	15	B01				
plate	15	B02				
plate	15	B03				
plate	15	B04	hsa-miR-374c-5p	MIMAT0018443	AUAAUACAACCUGCUAAGUGCU	8.903979759
plate	15	B05	hsa-miR-378e	MIMAT0018927	ACUGGACUUGGAGUCAGGA	19.45623419
plate	15	B06	hsa-miR-378h	MIMAT0018984	ACUGGACUUGGUGUCAGAUGG	31.32875662
plate	15	B07	hsa-miR-378b	MIMAT0014999	ACUGGACUUGGAGGCAGAA	-54.99303604
plate	15	B08	hsa-miR-378i	MIMAT0019074	ACUGGACUAGGAGUCAGAAGG	16.16050899
plate	15	B09	hsa-miR-378d	MIMAT0018926	ACUGGACUUGGAGUCAGAAA	-56.02650426
plate	15	B10	hsa-miR-378f	MIMAT0018932	ACUGGACUUGGAGCCAGAAG	1.936722735
plate	15	B11	hsa-miR-378g	MIMAT0018937	ACUGGGCUUGGAGUCAGAAG	-42.84360346
plate	15	B12				
plate	15	C01				

plate	15	C02	hsa-miR-382-3p	MIMAT0022697	AAUCAUUCACGGACAACACUU	-41.07335646
plate	15	C03	hsa-miR-450a-2-3p	MIMAT0031074	AUUGGGGACAUUUUGCAUUCAU	-24.58484082
plate	15	C04	hsa-miR-497-3p	MIMAT0004768	CAAACCACACUGUGGUGUUAGA	13.39140754
plate	15	C05	hsa-miR-506-5p	MIMAT0022701	UAUUCAGGAAGGUGUUACUUAA	10.14513058
plate	15	C06	hsa-miR-514a-5p	MIMAT0022702	UACUCUGGAGAGUGACAAUCAUG	-77.57357486
plate	15	C07	hsa-miR-514b-3p	MIMAT0015088	AUUGACACCUCUGUGAGUGGA	19.70347539
plate	15	C08	hsa-miR-514b-5p	MIMAT0015087	UUCUCAAGAGGGAGGCAAUCAU	-79.98170415
plate	15	C09	hsa-miR-539-3p	MIMAT0022705	AUCAUACAAGGACAAUUUCUUU	7.034836285
plate	15	C10	hsa-miR-545-5p	MIMAT0004785	UCAGUAAAUGUUUAUUAGAUGA	3.640214605
plate	15	C11	hsa-miR-548e-5p	MIMAT0026736	CAAAAGCAAUCGCGGUUUUUGC	-0.206858471
plate	15	C12				
plate	15	D01				
plate	15	D02	hsa-miR-548q	MIMAT0011163	GCUGGUGCAAAAGUAAUGGCGG	-4.034152251
plate	15	D03	hsa-miR-548o-3p	MIMAT0005919	CCAAAACUGCAGUUACUUUUGC	3.009749545
plate	15	D04	hsa-miR-548s	MIMAT0014987	AUGGCCAAAACUGCAGUUAUUUU	-0.444210023
plate	15	D05	hsa-miR-548am-3p	MIMAT0019076	CAAAAACUGCAGUUACUUUUGU	8.429276655
plate	15	D06	hsa-miR-548ab	MIMAT0018928	AAAAGUAAUUGUGGAUUUUGCU	19.28563776
plate	15	D07	hsa-miR-548g-5p	MIMAT0022722	UGCAAAAGUAAUUGCAGUUUUUG	-89.57713513
plate	15	D08	hsa-miR-548w	MIMAT0015060	AAAAGUAACUGCGGUUUUUGCCU	12.85736655
plate	15	D09	hsa-miR-548ak	MIMAT0019013	AAAAGUAACUGCGGUUUUUGA	-2.627349822
plate	15	D10	hsa-miR-548ag	MIMAT0018969	AAAGGUAAUUGUGGUUUCUGC	-1.828760745
plate	15	D11	hsa-miR-548e-3p	MIMAT0005874	AAAAACUGAGACUACUUUUGCA	-9.337475997
plate	15	D12				
plate	15	E01				
plate	15	E02	hsa-miR-548a-3p	MIMAT0003251	CAAAACUGGCAAUUACUUUUGC	-33.50777574
plate	15	E03	hsa-miR-561-5p	MIMAT0022706	AUCAAGGAUCUUAAACUUUGCC	-12.10904985
plate	15	E04	hsa-miR-579-5p	MIMAT0026616	UCGCGGUUUGUGCCAGAUGACG	-9.043258969
plate	15	E05	hsa-miR-584-3p	MIMAT0022708	UCAGUUCCAGGCCAACCAGGCU	-104.3250727
plate	15	E06	hsa-miR-619-5p	MIMAT0026622	GCUGGGAUUACAGGCAUGAGCC	-45.30118099
plate	15	E07	hsa-miR-642a-3p	MIMAT0020924	AGACACAUUUGGAGAGGGAACC	6.542826297
plate	15	E08	hsa-miR-642b-3p	MIMAT0018444	AGACACAUUUGGAGAGGGACCC	18.61314169
plate	15	E09	hsa-miR-652-5p	MIMAT0022709	CAACCCUAGGAGAGGGUGCCAUUCA	-18.68072096
plate	15	E10	hsa-miR-659-5p	MIMAT0022710	AGGACCUUCCCUGAACCAAGGA	-58.47666455
plate	15	E11	hsa-miR-660-3p	MIMAT0022711	ACCUCCUGUGUGCAUGGAUUA	-58.24178541
plate	15	E12				
plate	15	F01				
plate	15	F02	hsa-miR-664b-3p	MIMAT0022272	UUCAUUUGCCUCCCAGCCUACA	-96.73971271
plate	15	F03	hsa-miR-664a-5p	MIMAT0005948	ACUGGCUAGGGAAAAUGAUUGGAU	-43.1205136
plate	15	F04	hsa-miR-664b-5p	MIMAT0022271	UGGGCUAAGGGAGAUGAUUGGGUA	-3.895697179
plate	15	F05	hsa-miR-744-3p	MIMAT0004946	CUGUUGCCACUAACCUCAACCU	-79.32898738
plate	15	F06	hsa-miR-766-5p	MIMAT0022714	AGGAGGAAUUGGUGCUGGUCUU	12.60270812
plate	15	F07	hsa-miR-873-3p	MIMAT0022717	GGAGACUGAUGAGUUCCCGGGA	-69.80278394
plate	15	F08	hsa-miR-874-5p	MIMAT0026718	CGGCCCCACGCACCAGGGUAAGA	-17.92416288
plate	15	F09	hsa-miR-887-3p	MIMAT0004951	GUGAACGGGCGCCAUCCCGAGG	8.607290319
plate	15	F10	hsa-miR-1185-2-3p	MIMAT0022713	AUAUACAGGGGGAGACUCUCAU	-96.03013046
plate	15	F11	hsa-miR-1185-1-3p	MIMAT0022838	AUAUACAGGGGGAGACUCUUAU	-94.40081095
plate	15	F12				
plate	15	G01				
plate	15	G02	hsa-miR-1247-3p	MIMAT0022721	CCCCGGGAACGUCGAGACUGGAGC	-63.11738188

plate	15	G03	hsa-miR-1260b	MIMAT0015041	AUCCCACCACUGCCACCAU	-110.429458
plate	15	G04	hsa-miR-1269b	MIMAT0019059	CUGGACUGAGCCAUGCUACUGG	-39.69869539
plate	15	G05	hsa-miR-1271-3p	MIMAT0022712	AGUGCCUGCUAUGUGCCAGGCA	-9.243524341
plate	15	G06	hsa-miR-1273g-3p	MIMAT0022742	ACCACUGCACUCCAGCCUGAG	-77.68977822
plate	15	G07	hsa-miR-1276	MIMAT0005930	UAAAGAGCCCUGUGGAGACA	-24.51561328
plate	15	G08	hsa-miR-1277-3p	MIMAT0005933	UACGUAGAUAUAUAUGUAUUUU	13.98973125
plate	15	G09	hsa-miR-1287-5p	MIMAT0005878	UGCUGGAUCAGUGGUUCGAGUC	0.304930814
plate	15	G10	hsa-miR-1294	MIMAT0005884	UGUGAGGUUGGCAUUGUUGUCU	-11.96317754
plate	15	G11	hsa-miR-1295a	MIMAT0005885	UUAGGCCGCAGAUCUGGGUGA	-27.1141183
plate	15	G12				
plate	15	H01				
plate	15	H02				
plate	15	H03				
plate	15	H04				
plate	15	H05				
plate	15	H06				
plate	15	H07				
plate	15	H08				
plate	15	H09				
plate	15	H10				
plate	15	H11				
plate	15	H12				
plate	16	A01				
plate	16	A02				
plate	16	A03				
plate	16	A04				
plate	16	A05				
plate	16	A06				
plate	16	A07				
plate	16	A08				
plate	16	A09				
plate	16	A10				
plate	16	A11				
plate	16	A12				
plate	16	B01				
plate	16	B02				
plate	16	B03				
plate	16	B04	hsa-miR-1304-3p	MIMAT0022720	UCUCACUGUAGCCUCGAACCCC	-75.36456138
plate	16	B05	hsa-miR-1306-5p	MIMAT0022726	CCACCUCCCCUGCAAACGUCCA	-44.99736218
plate	16	B06	hsa-miR-1307-5p	MIMAT0022727	UCGACCGGACCUCGACCGGCU	-6.472116135
plate	16	B07	hsa-miR-2116-3p	MIMAT0011161	CCUCCCAUGCCAAGAACUCCC	-15.78423924
plate	16	B08	hsa-miR-2277-5p	MIMAT0017352	AGCGCGGGCUGAGCGCUGCCAGUC	-15.20492931
plate	16	B09	hsa-miR-2355-3p	MIMAT0017950	AUUGUCCUUGCUGUUUGGAGAU	-22.29977206
plate	16	B10	hsa-miR-3117-3p	MIMAT0014979	AUAGGACUCAUAUAGUGCCAG	-44.61115557
plate	16	B11	hsa-miR-3123	MIMAT0014985	CAGAGAAUUGUUUAAUC	-5.03179263
plate	16	B12				
plate	16	C01				
plate	16	C02	hsa-miR-3124-5p	MIMAT0014986	UUCGCGGGCGAAGGCAAAGUC	-35.52848463
plate	16	C03	hsa-miR-3127-5p	MIMAT0014990	AUCAGGGCUUGUGGAAUGGGAAG	-9.245988373

plate	16	C04	hsa-miR-3131	MIMAT0014996	UCGAGGACUGGUGGAAGGGCCUU	-19.61677197
plate	16	C05	hsa-miR-3135b	MIMAT0018985	GGCUGGAGCGAGUGCAGUGGUG	-29.45368171
plate	16	C06	hsa-miR-3136-5p	MIMAT0015003	CUGACUGAAUAGGUAGGGUCAUU	-17.71981593
plate	16	C07	hsa-miR-3138	MIMAT0015006	UGUGGACAGUGAGGUAGAGGGAGU	-27.55445387
plate	16	C08	hsa-miR-3140-3p	MIMAT0015008	AGCUUUUGGGAAUUCAGGUAGU	-24.64427341
plate	16	C09	hsa-miR-3141	MIMAT0015010	GAGGGCGGGUGGAGGAGGA	11.52284046
plate	16	C10	hsa-miR-3143	MIMAT0015012	AUAACAUUGUAAAGCGCUUCUUUCG	-13.52606642
plate	16	C11	hsa-miR-3144-5p	MIMAT0015014	AGGGGACCAAAGAGAUAUAUAG	-24.20581531
plate	16	C12				
plate	16	D01				
plate	16	D02	hsa-miR-3147	MIMAT0015019	GGUUGGGCAGUGAGGAGGGUGUGA	-10.97483094
plate	16	D03	hsa-miR-3150b-3p	MIMAT0018194	UGAGGAGAUCGUCGAGGUUGG	-24.98050035
plate	16	D04	hsa-miR-3158-3p	MIMAT0015032	AAGGGCUUCCUCUCUGCAGGAC	-28.87437178
plate	16	D05	hsa-miR-3164	MIMAT0015038	UGUGACUUUAAGGGAAAUGGCG	-43.1322114
plate	16	D06	hsa-miR-3168	MIMAT0015043	GAGUUCUACAGUCAGAC	-3.043964449
plate	16	D07	hsa-miR-3175	MIMAT0015052	CGGGGAGAGAACGCAGUGACGU	8.201463546
plate	16	D08	hsa-miR-3178	MIMAT0015055	GGGGCGCGGCCGGAUCG	-23.5083716
plate	16	D09	hsa-miR-3180	MIMAT0018178	UGGGGCGGAGCUUCCGGAG	-12.26748721
plate	16	D10	hsa-miR-3180-5p	MIMAT0015057	CUUCCAGACGCUCCGCCCCACGUCG	-18.08330452
plate	16	D11	hsa-miR-3182	MIMAT0015062	GCUUCUGUAGUGUAGUC	-4.672847655
plate	16	D12				
plate	16	E01				
plate	16	E02	hsa-miR-3187-3p	MIMAT0015069	UUGGCCAUGGGGCUGCGCGG	-15.67064905
plate	16	E03	hsa-miR-3190-3p	MIMAT0022839	UGUGGAAGGUAGACGGCCAGAGA	-15.5752333
plate	16	E04	hsa-miR-3192-5p	MIMAT0015076	UCUGGGAGGUUGUAGCAGUGGAA	-36.15095883
plate	16	E05	hsa-miR-3198	MIMAT0015083	GUGGAGUCCUGGGGAAUGGAGA	-28.5086114
plate	16	E06	hsa-miR-3199	MIMAT0015084	AGGGACUGCCUUAGGAGAAAGUU	-1.271957613
plate	16	E07	hsa-miR-3200-5p	MIMAT0017392	AAUCUGAGAAGGCGCACAAGGU	-11.61320776
plate	16	E08	hsa-miR-3200-3p	MIMAT0015085	CACCUUGCGCUACUCAGGUCUG	-36.79387926
plate	16	E09	hsa-miR-3605-5p	MIMAT0017981	UGAGGAUGGAUAGCAAGGAAGCC	-10.33872592
plate	16	E10	hsa-miR-3607-5p	MIMAT0017984	GCAUGUGAUGAAGCAAAUCAGU	-58.12157179
plate	16	E11	hsa-miR-3609	MIMAT0017986	CAAAGUGAUGAGUAAUACUGGCUG	-31.24613478
plate	16	E12				
plate	16	F01				
plate	16	F02	hsa-miR-3611	MIMAT0017988	UUGUGAAGAAAGAAAUUCUUA	-11.59503333
plate	16	F03	hsa-miR-3613-5p	MIMAT0017990	UGUUGUACUUUUUUUUUUGUUC	-7.2627038
plate	16	F04	hsa-miR-3613-3p	MIMAT0017991	ACAAAAAAAAAAGCCCAACCCUUC	-79.59465975
plate	16	F05	hsa-miR-3614-5p	MIMAT0017992	CCACUUGGAUCUGAAGGCUGCCC	-66.47953736
plate	16	F06	hsa-miR-3615	MIMAT0017994	UCUCUCGGCUCCUCGCGGCUC	-0.690375883
plate	16	F07	hsa-miR-3622a-5p	MIMAT0018003	CAGGCACGGGAGCUCAGGUGAG	-28.7607816
plate	16	F08	hsa-miR-3656	MIMAT0018076	GGCGGGUGCGGGGGUGG	0.338751164
plate	16	F09	hsa-miR-3664-3p	MIMAT0019220	UCUCAGGAGUAAAGACAGAGUU	-57.74899599
plate	16	F10	hsa-miR-3679-5p	MIMAT0018104	UGAGGAUAUGGCAGGGAAGGGGA	-39.83355252
plate	16	F11	hsa-miR-3687	MIMAT0018115	CCCGGACAGGCGUUCGUGCGACGU	-9.559497275
plate	16	F12				
plate	16	G01				
plate	16	G02	hsa-miR-3690	MIMAT0018119	ACCUGGACCCAGCGUAGACAAAG	-43.9477889
plate	16	G03	hsa-miR-3909	MIMAT0018183	UGUCCUCUAGGGCCUGCAGUCU	-11.27016541
plate	16	G04	hsa-miR-3913-5p	MIMAT0018187	UUUGGGACUGAUCUUGAUGUCU	-58.91443125

plate	16	G05	hsa-miR-3916	MIMAT0018190	AAGAGGAAGAAAUGGCUGGUUCUCAG	-48.14835383
plate	16	G06	hsa-miR-3928-3p	MIMAT0018205	GGAGGAACCUUGGAGCUUCGGC	12.69736294
plate	16	G07	hsa-miR-3929	MIMAT0018206	GAGGCUGAUGUGAGUAGACCACU	6.481708194
plate	16	G08	hsa-miR-3934-5p	MIMAT0018349	UCAGGUGUGGAAACUGAGGCAG	-59.41877166
plate	16	G09	hsa-miR-3960	MIMAT0019337	GGCGGCGGCGGAGGCGGGGG	13.89233165
plate	16	G10	hsa-miR-4284	MIMAT0016915	GGGCUCACAUCACCCCAU	-45.67663147
plate	16	G11	hsa-miR-4328	MIMAT0016926	CCAGUUUUCCCAGGAUU	-10.67722466
plate	16	G12				
plate	16	H01				
plate	16	H02				
plate	16	H03				
plate	16	H04				
plate	16	H05				
plate	16	H06				
plate	16	H07				
plate	16	H08				
plate	16	H09				
plate	16	H10				
plate	16	H11				
plate	16	H12				
plate	17	A01				
plate	17	A02				
plate	17	A03				
plate	17	A04				
plate	17	A05				
plate	17	A06				
plate	17	A07				
plate	17	A08				
plate	17	A09				
plate	17	A10				
plate	17	A11				
plate	17	A12				
plate	17	B01				
plate	17	B02				
plate	17	B03				
plate	17	B04	hsa-miR-4417	MIMAT0018929	GGUGGGCUUCCCGGAGGG	29.68172583
plate	17	B05	hsa-miR-4419a	MIMAT0018931	UGAGGGAGGAGACUGCA	-77.99265971
plate	17	B06	hsa-miR-4421	MIMAT0018934	ACCUGUCUGUGGAAAGGAGCUA	-11.82307322
plate	17	B07	hsa-miR-4425	MIMAT0018940	UGUUGGGAUUCAGCAGGACCAU	27.37619587
plate	17	B08	hsa-miR-4429	MIMAT0018944	AAAAGCUGGGCUGAGAGGCG	-3.968492065
plate	17	B09	hsa-miR-4435	MIMAT0018951	AUGGCCAGAGCUCACACAGAGG	-39.05462557
plate	17	B10	hsa-miR-4443	MIMAT0018961	UUGGAGGCGUGGGUUUU	35.36227113
plate	17	B11	hsa-miR-4448	MIMAT0018967	GGCUCCUUGGUCUAGGGGUA	31.03348522
plate	17	B12				
plate	17	C01				
plate	17	C02	hsa-miR-4451	MIMAT0018973	UGGUAGAGCUGAGGACA	25.10047124
plate	17	C03	hsa-miR-4454	MIMAT0018976	GGAUCCGAGUCACGGCACCA	49.63025806
plate	17	C04	hsa-miR-4455	MIMAT0018977	AGGGUGUGUGUGUUUUU	19.85999287
plate	17	C05	hsa-miR-4458	MIMAT0018980	AGAGGUAGGUGUGGAAGAA	22.3566276

plate	17	C06	hsa-miR-4459	MIMAT0018981	CCAGGAGGCGGAGGAGGUGGAG	2.472965786
plate	17	C07	hsa-miR-4461	MIMAT0018983	GAUUGAGACUAGUAGGGCUAGGC	13.86035521
plate	17	C08	hsa-miR-4484	MIMAT0019018	AAAAGGCGGGAGAAGCCCCA	1.813742011
plate	17	C09	hsa-miR-4485	MIMAT0019019	UAACGGCCGCGGUACCCUAA	24.76209307
plate	17	C10	hsa-miR-4492	MIMAT0019027	GGGGCUGGGCGCGCGCC	48.56077268
plate	17	C11	hsa-miR-4497	MIMAT0019032	CUCCGGGACGGCUGGGC	45.53114852
plate	17	C12				
plate	17	D01				
plate	17	D02	hsa-miR-4500	MIMAT0019036	UGAGGUAGUAGUUUCUU	8.409486277
plate	17	D03	hsa-miR-4502	MIMAT0019038	GCUGAUGAUGAUGGUGCUGAAG	13.98132979
plate	17	D04	hsa-miR-4508	MIMAT0019045	GCGGGGCUGGGCGCGCG	31.79615102
plate	17	D05	hsa-miR-4510	MIMAT0019047	UGAGGGAGUAGGAUGUAUGGUU	-12.41917983
plate	17	D06	hsa-miR-4516	MIMAT0019053	GGGAGAAGGGUCGGGGC	28.91730677
plate	17	D07	hsa-miR-4521	MIMAT0019058	GCUAAGGAAGUCCUGUGCUCAG	-61.15440135
plate	17	D08	hsa-miR-4525	MIMAT0019064	GGGGGGAUGUGCAUGCUGGUU	-30.62147036
plate	17	D09	hsa-miR-4531	MIMAT0019070	AUGGAGAAGGCUUCUGA	26.70645256
plate	17	D10	hsa-miR-4647	MIMAT0019709	GAAGAUGGUGCUGUGCUGAGGAA	26.34528209
plate	17	D11	hsa-miR-4667-5p	MIMAT0019743	ACUGGGGAGCAGAAGGAGAACC	20.44382669
plate	17	D12				
plate	17	E01				
plate	17	E02	hsa-miR-4695-3p	MIMAT0019789	UGAUCUCACCGCUGCCUCCUUC	-39.15280784
plate	17	E03	hsa-miR-4741	MIMAT0019871	CGGGCUGUCCGGAGGGGUCGGCU	32.28706234
plate	17	E04	hsa-miR-4791	MIMAT0019963	UGGAUAUGAUGACUGAAA	27.42879351
plate	17	E05	hsa-miR-4804-5p	MIMAT0019984	UUGGACGGUAAGGUUAAGCAA	26.78008926
plate	17	E06	hsa-miR-4999-5p	MIMAT0021017	UGCUGUAUUGUCAGGUAGUGA	25.86664355
plate	17	E07	hsa-miR-5001-5p	MIMAT0021021	AGGGCUGGACUCAGCGGCGGAGCU	24.90936647
plate	17	E08	hsa-miR-5010-5p	MIMAT0021043	AGGGGGAUGGCAGAGCAAAAUU	33.94914782
plate	17	E09	hsa-miR-5090	MIMAT0021082	CCGGGGCAGAUUGGUGUAGGGUG	37.96585439
plate	17	E10	hsa-miR-5095	MIMAT0020600	UUACAGGCGUGAACCACCGCG	-77.26681225
plate	17	E11	hsa-miR-5096	MIMAT0020603	GUUUCACCAUGUUGGUCAGGC	-32.21693215
plate	17	E12				
plate	17	F01				
plate	17	F02	hsa-miR-5100	MIMAT0022259	UUCAGAUCCCAGCGGUGCCUCU	-23.93806335
plate	17	F03	hsa-miR-5187-5p	MIMAT0021117	UGGGAUGAGGGAUUGAAGUGGA	17.75433395
plate	17	F04	hsa-miR-5196-3p	MIMAT0021129	UCAUCCUCGUCUCCCUCCCAG	40.53612581
plate	17	F05	hsa-miR-5571-5p	MIMAT0022257	CAAUUCUCAAAGGAGCCUCCC	20.21239707
plate	17	F06	hsa-miR-5585-3p	MIMAT0022286	CUGAAUAGCUGGGACUACAGGU	-32.17310079
plate	17	F07	hsa-miR-5699-5p	MIMAT0027103	UGCCCCAACAAGGAAGGACAAG	-95.94949458
plate	17	F08	hsa-miR-6087	MIMAT0023712	UGAGGCGGGGGGGCGAGC	34.51369584
plate	17	F09	hsa-miR-6126	MIMAT0024599	GUGAAGGCCCGGCGGAGA	32.84810386
plate	17	F10	hsa-miR-6128	MIMAT0024611	ACUGGAAUUGGAGUCAAAA	30.55659993
plate	17	F11	hsa-miR-6129	MIMAT0024613	UGAGGGAGUUGGGUGUAUA	6.785972404
plate	17	F12				
plate	17	G01				
plate	17	G02	hsa-miR-6130	MIMAT0024614	UGAGGGAGUGGAUUGUAUG	4.808301077
plate	17	G03	hsa-miR-6133	MIMAT0024617	UGAGGGAGGAGGUUGGGUA	20.24570891
plate	17	G04	hsa-miR-6134	MIMAT0024618	UGAGGUGGUAGGAUGUAGA	2.692122627
plate	17	G05	hsa-miR-6503-3p	MIMAT0025463	GGGACUAGGAUGCAGACCUCC	-16.7882906
plate	17	G06	hsa-miR-6503-5p	MIMAT0025462	AGGUCUGCAUUCAAAUCCCCAGA	-43.51665884

plate	17	G07	hsa-miR-6510-3p	MIMAT0025477	CACCGACUCUGUCUCCUGCAG	29.56951752
plate	17	G08	hsa-miR-6511a-5p	MIMAT0025478	CAGGCAGAAGUGGGGCUGACAGG	26.59950402
plate	17	G09	hsa-miR-6515-5p	MIMAT0025486	UUGGAGGGUGUGGAAGACAUC	-34.20336976
plate	17	G10	hsa-miR-6720-3p	MIMAT0025851	CGCGCCUGCAGGAACUGGUAGA	34.98356811
plate	17	G11	hsa-miR-6721-5p	MIMAT0025852	UGGGCAGGGGCUUAUUGUAGGAG	8.819747882
plate	17	G12				
plate	17	H01				
plate	17	H02				
plate	17	H03				
plate	17	H04				
plate	17	H05				
plate	17	H06				
plate	17	H07				
plate	17	H08				
plate	17	H09				
plate	17	H10				
plate	17	H11				
plate	17	H12				
plate	18	A01				
plate	18	A02				
plate	18	A03				
plate	18	A04				
plate	18	A05				
plate	18	A06				
plate	18	A07				
plate	18	A08				
plate	18	A09				
plate	18	A10				
plate	18	A11				
plate	18	A12				
plate	18	B01				
plate	18	B02				
plate	18	B03				
plate	18	B04	hsa-miR-6723-5p	MIMAT0025855	AUAGUCCGAGUAACGUCGGGGC	23.0373556
plate	18	B05	hsa-miR-6724-5p	MIMAT0025856	CUGGGCCCGCGGCGGGCGUGGGG	22.01252785
plate	18	B06	hsa-miR-6735-5p	MIMAT0027371	CAGGGCAGAGGGCACAGGAAUCUGA	-2.852306353
plate	18	B07	hsa-miR-6767-5p	MIMAT0027434	UCGCAGACAGGGACACAUGGAGA	-15.95125415
plate	18	B08	hsa-miR-6803-5p	MIMAT0027506	CUGGGGGUGGGGGGCUGGGCGU	18.70048875
plate	18	B09	hsa-miR-6805-5p	MIMAT0027510	UAGGGGGCGGCUUGUGGAGUGU	25.93907096
plate	18	B10	hsa-miR-6839-5p	MIMAT0027580	UCUGGAUUGAAGAGACGACCCA	-23.3940166
plate	18	B11	hsa-miR-6852-5p	MIMAT0027604	CCCUGGGGUUCUGAGGACAUG	-10.03591696
plate	18	B12				
plate	18	C01				
plate	18	C02	hsa-miR-6882-5p	MIMAT0027664	UACAAGUCAGGAGCUGAAGCAG	-68.20765305
plate	18	C03	hsa-miR-7641	MIMAT0029782	UUGAUCUCGGAAGCUAAGC	11.7819198
plate	18	C04	hsa-miR-7704	MIMAT0030019	CGGGGUCGGCGGCGACGUG	22.39536581
plate	18	C05	hsa-miR-7977	MIMAT0031180	UUCCCAGCCAACGCACCA	-5.492906591
plate	18	C06	hsa-miR-1910-3p	MIMAT0026917	GAGGCAGAAGCAGGAUGACA	25.03792932
plate	18	C07	hsa-miR-759	MIMAT0010497	GCAGAGUGCAAACAAUUUUGAC	14.89174193

plate	18	C08	hsa-miR-3653	MIMAT0018073	CUAAGAAGUUGACUGAAG	36.07347871
plate	18	C09	hsa-miR-3691-5p	MIMAT0018120	AGUGGAUGAUGGAGACUCGGUAC	26.41810922
plate	18	C10	hsa-miR-4326	MIMAT0016888	UGUUCCUCUGUCUCCCAGAC	13.35450031
plate	18	C11	hsa-miR-4524a-3p	MIMAT0019063	UGAGACAGGCUUAUGCUGCUAU	-27.15171835
plate	18	C12				
plate	18	D01				
plate	18	D02	hsa-miR-4423-3p	MIMAT0018936	AUAGGCACCAAAAAGCAACAA	-56.75000027
plate	18	D03	hsa-miR-4753-5p	MIMAT0019890	CAAGGCCAAAGGAAGAGAACAG	-58.38540548
plate	18	D04	hsa-miR-4750-5p	MIMAT0019887	CUCGGGCGGAGGUGGUUGAGUG	18.93411806
plate	18	D05	hsa-miR-6731-5p	MIMAT0027363	UGGGAGAGCAGGGUAUUGUGGA	20.06496247
plate	18	D06	hsa-miR-887-5p	MIMAT0026720	CUUGGGAGCCCUGUUAGACUC	-33.45382004
plate	18	D07	hsa-miR-3129-5p	MIMAT0014992	GCAGUAGUGUAGAGAUUGGUUU	36.64871727
plate	18	D08	hsa-miR-6511b-3p	MIMAT0025848	CCUCACCACCCCUUCUGCCUGCA	-9.236865441
plate	18	D09	hsa-miR-6511a-3p	MIMAT0025479	CCUCACCAUCCCUUCUGCCUGC	-43.24858181
plate	18	D10	hsa-miR-6825-5p	MIMAT0027550	UGGGGAGGUGUGGAGUCAGCAU	17.1436144
plate	18	D11	hsa-miR-548u	MIMAT0015013	CAAAGACUGCAAUUACUUUUGCG	-32.44666173
plate	18	D12				
plate	18	E01				
plate	18	E02	hsa-miR-3940-3p	MIMAT0018356	CAGCCCGGAUCCCAGCCCACUU	-2.194610383
plate	18	E03	hsa-miR-518e-5p	MIMAT0005450	CUCUAGAGGGAAGCGCUUUCUG	14.41663022
plate	18	E04	hsa-miR-548t-5p	MIMAT0015009	CAAAAGUGAUCGUGGUUUUUG	9.477038994
plate	18	E05	hsa-miR-3662	MIMAT0018083	GAAAAUGAUGAGUAGUGACUGAUG	10.25056799
plate	18	E06	hsa-miR-4787-5p	MIMAT0019956	GCGGGGGUGGCGGCGGCAUCCC	39.25790517
plate	18	E07	hsa-miR-3910	MIMAT0018184	AAAGGCAUAAAACCAAGACA	39.16759468
plate	18	E08	hsa-miR-4520b-3p	MIMAT0020300	UUUGGACAGAAAACACGCAGGU	-19.69324973
plate	18	E09	hsa-miR-711	MIMAT0012734	GGGACCCAGGGAGAGACGUAAG	-7.116532164
plate	18	E10	hsa-miR-1908-3p	MIMAT0026916	CCGGCCGCCGGCUCCGCCCCG	30.70589429
plate	18	E11	hsa-miR-3606-5p	MIMAT0017983	UUAGUGAAGGCUAUUUUAAUU	19.97857853
plate	18	E12				
plate	18	F01				
plate	18	F02	hsa-miR-4710	MIMAT0019815	GGGUGAGGGCAGGUGGUU	3.809074023
plate	18	F03	hsa-miR-6514-5p	MIMAT0025484	UAUGGAGUGGACUUUCAGCUGGC	-21.67222745
plate	18	F04	hsa-miR-3135a	MIMAT0015001	UGCCUAGGCUGAGACUGCAGUG	10.91022723
plate	18	F05	hsa-miR-4746-5p	MIMAT0019880	CCGGUCCCAGGAGAACCUGCAGA	-5.064913392
plate	18	F06	hsa-miR-548ai	MIMAT0018989	AAAGGUAAUUGCAGUUUUUCCC	25.18517468
plate	18	F07	hsa-miR-3173-3p	MIMAT0015048	AAAGGAGGAAAUAGGCAGGCCA	-1.739131383
plate	18	F08	hsa-miR-3677-3p	MIMAT0018101	CUCGUGGGCUCUGGCCACGGCC	29.39442889
plate	18	F09	hsa-miR-2278	MIMAT0011778	GAGAGCAGUGUGUGUUGCCUGG	36.9785469
plate	18	F10	hsa-miR-4444	MIMAT0018962	CUCGAGUUGGAAGAGGCG	1.924333333
plate	18	F11	hsa-miR-4739	MIMAT0019868	AAGGGAGGAGGAGCGGAGGGGCCCU	16.48199189
plate	18	F12				
plate	18	G01				
plate	18	G02	hsa-miR-5091	MIMAT0021083	ACGGAGACGACAAGACUGUGCUG	64.36029523
plate	18	G03	hsa-miR-6793-5p	MIMAT0027486	UGUGGGUUCUGGGUUGGGGUGA	41.16227857
plate	18	G04	hsa-miR-6875-5p	MIMAT0027650	UGAGGGACCCAGGACAGGAGA	20.08459519
plate	18	G05	hsa-miR-3121-3p	MIMAT0014983	UAAAUAGAGUAGGCAAAGGACA	36.37189599
plate	18	G06	hsa-miR-3648	MIMAT0018068	AGCCGCGGGGAUCGCCGAGGG	50.81372152
plate	18	G07	hsa-miR-6780a-5p	MIMAT0027460	UUGGGAGGGAAGACAGCUGGAGA	-11.2413657
plate	18	G08	hsa-miR-210-5p	MIMAT0026475	AGCCCCUGCCCACCGCACACUG	35.19393305

plate	18	G09	hsa-miR-6734-5p	MIMAT0027369	UUGAGGGGAGAAUGAGGUGGAGA	15.58281352
plate	18	G10	hsa-miR-4423-5p	MIMAT0019232	AGUUGCCUUUUUGUUCCCAUGC	46.96963582
plate	18	G11	hsa-miR-4745-5p	MIMAT0019878	UGAGUGGGGCUCCCGGGACGGCG	23.26705838
plate	18	G12				
plate	18	H01				
plate	18	H02				
plate	18	H03				
plate	18	H04				
plate	18	H05				
plate	18	H06				
plate	18	H07				
plate	18	H08				
plate	18	H09				
plate	18	H10				
plate	18	H11				
plate	18	H12				
plate	19	A01				
plate	19	A02				
plate	19	A03				
plate	19	A04				
plate	19	A05				
plate	19	A06				
plate	19	A07				
plate	19	A08				
plate	19	A09				
plate	19	A10				
plate	19	A11				
plate	19	A12				
plate	19	B01				
plate	19	B02				
plate	19	B03				
plate	19	B04	hsa-miR-3176	MIMAT0015053	ACUGGCCUGGGACUACCGG	11.99869243
plate	19	B05	hsa-miR-3614-3p	MIMAT0017993	UAGCCUUCAGAUCUUGGUGUUUU	54.05855163
plate	19	B06	hsa-miR-4298	MIMAT0016852	CUGGGACAGGAGGAGGAGGCAG	29.07545758
plate	19	B07	hsa-miR-4271	MIMAT0016901	GGGGGAAGAAAAGGUGGGG	45.44096028
plate	19	B08	hsa-miR-6089	MIMAT0023714	GGAGGCCGGGGUGGGGCGGGGCGG	27.44601994
plate	19	B09	hsa-miR-6877-5p	MIMAT0027654	AGGGCCGAAGGGUGGAAGCUGC	24.46680012
plate	19	B10	hsa-miR-128-1-5p	MIMAT0026477	CGGGGCCGUAGCACUGUCUGAGA	39.67973432
plate	19	B11	hsa-miR-412-5p	MIMAT0026557	UGGUCGACCAGUUGGAAAGUAAU	-25.16638782
plate	19	B12				
plate	19	C01				
plate	19	C02	hsa-miR-500b-3p	MIMAT0027032	GCACCCAGGCAAGGAUUCUG	-51.32468144
plate	19	C03	hsa-miR-4478	MIMAT0019006	GAGGCUGAGCUGAGGAG	35.12765455
plate	19	C04	hsa-miR-5585-5p	MIMAT0022285	UGAAGUACCAGCUACUCGAGAG	-76.57611541
plate	19	C05	hsa-miR-4651	MIMAT0019715	CGGGGUGGGUGAGGUCGGGC	27.87439392
plate	19	C06	hsa-miR-605-3p	MIMAT0026621	AGAAGGCACUAUGAGAUUUAGA	29.35511305
plate	19	C07	hsa-miR-3917	MIMAT0018191	GCUCGGACUGAGCAGGUGGG	40.21318117
plate	19	C08	hsa-miR-1273h-5p	MIMAT0030415	CUGGGAGGUCAAGGCUGCAGU	26.99824789
plate	19	C09	hsa-miR-3918	MIMAT0018192	ACAGGGCCGCAGAUGGAGACU	29.79803559

plate 19	C10	hsa-miR-4486	MIMAT0019020	GCUGGGCGAGGCUGGCA	30.66286608
plate 19	C11	hsa-miR-6826-5p	MIMAT0027552	UCAAUAGGAAAGAGGUGGGACCU	-56.59933921
plate 19	C12				
plate 19	D01				
plate 19	D02	hsa-miR-7847-3p	MIMAT0030422	CGUGGAGGACGAGGAGGAGGC	-8.663482151
plate 19	D03	hsa-miR-3153	MIMAT0015026	GGGGAAAGCGAGUAGGGACAUUU	16.00924282
plate 19	D04	hsa-miR-3159	MIMAT0015033	UAGGAUUACAAGUGUCGGCCAC	33.04882835
plate 19	D05	hsa-miR-4517	MIMAT0019054	AAAUAUGAUGAAACUCACAGCUGAG	35.73061114
plate 19	D06	hsa-miR-4724-5p	MIMAT0019841	AACUGAACCAGGAGUGAGCUUCG	-14.7399267
plate 19	D07	hsa-miR-4800-5p	MIMAT0019978	AGUGGACCGAGGAAGGAAGGA	29.6590161
plate 19	D08	hsa-miR-3145-3p	MIMAT0015016	AGAUAUUUUGAGUGUUUGGAAUUG	41.20086609
plate 19	D09	hsa-miR-3610	MIMAT0017987	GAAUCGGAAAGGAGGCGCCG	39.70559841
plate 19	D10	hsa-miR-3667-5p	MIMAT0018089	AAAGACCCAUUGAGGAGAAGGU	-3.788101262
plate 19	D11	hsa-miR-4800-3p	MIMAT0019979	CAUCCGUCCGUCUGUCCAC	23.23502285
plate 19	D12				
plate 19	E01				
plate 19	E02	hsa-miR-1236-3p	MIMAT0005591	CCUCUUCCCCUUGUCUCUCCAG	9.55453596
plate 19	E03	hsa-miR-3616-3p	MIMAT0017996	CGAGGGCAUUUCAUGAUGCAGGC	-35.23883421
plate 19	E04	hsa-miR-627-3p	MIMAT0026623	UCUUUUCUUUGAGACUCACU	21.53445896
plate 19	E05	hsa-miR-3162-3p	MIMAT0019213	UCCCUACCCCUCCACUCCCCA	-13.28507166
plate 19	E06	hsa-miR-4687-3p	MIMAT0019775	UGGCUGUUGGAGGGGGCAGGC	34.40669305
plate 19	E07	hsa-miR-4487	MIMAT0019021	AGAGCUGGCUGAAGGGCAG	12.73743549
plate 19	E08	hsa-miR-653-3p	MIMAT0026625	UUCACUGGAGUUUGUUUCAAUA	25.85052891
plate 19	E09	hsa-miR-3177-3p	MIMAT0015054	UGCACGGCACUGGGGACACGU	17.93450099
plate 19	E10	hsa-miR-4727-3p	MIMAT0019848	AUAGUGGGAAGCUGGCAGAUUC	14.14379536
plate 19	E11	hsa-miR-3620-5p	MIMAT0022967	GUGGGCUGGGCUGGGCUGGGCC	18.93835097
plate 19	E12				
plate 19	F01				
plate 19	F02	hsa-miR-5571-3p	MIMAT0022258	GUCCUAGGAGGCUCCUCUG	-2.01802763
plate 19	F03	hsa-miR-942-3p	MIMAT0026734	CACAUGGCCGAAACAGAGAAGU	-50.25293822
plate 19	F04	hsa-miR-3157-5p	MIMAT0015031	UUCAGCCAGGCUAGUGCAGUCU	17.39297161
plate 19	F05	hsa-miR-4688	MIMAT0019777	UAGGGGCAGCAGAGGACCUGGG	-2.171595662
plate 19	F06	hsa-miR-4716-3p	MIMAT0019827	AAGGGGGAAGGAAACAUGGAGA	-21.41286182
plate 19	F07	hsa-miR-3154	MIMAT0015028	CAGAAGGGGAGUUGGGAGCAGA	38.88118055
plate 19	F08	hsa-miR-4472	MIMAT0018999	GGUGGGGGGUGUUGUUUU	24.88547507
plate 19	F09	hsa-miR-4707-3p	MIMAT0019808	AGCCCGCCCCAGCCGAGGUUCU	-4.368426772
plate 19	F10	hsa-miR-219b-3p	MIMAT0019748	AGAAUUGCGUUUGGACAAUCAGU	-19.02689955
plate 19	F11	hsa-miR-651-3p	MIMAT0026624	AAAGGAAAGUGUAUCCUAAAAG	23.93658628
plate 19	F12				
plate 19	G01				
plate 19	G02	hsa-miR-3162-5p	MIMAT0015036	UUAGGGAGUAGAAGGGUGGGGAG	17.42045221
plate 19	G03	hsa-miR-6127	MIMAT0024610	UGAGGGAGUGGGUGGGAGG	2.192969458
plate 19	G04	hsa-miR-433-5p	MIMAT0026554	UACGGUGAGCCUGUCAUUAUUC	11.25186684
plate 19	G05	hsa-miR-3133	MIMAT0014998	UAAAGAACUCUUAAAACCCAAU	23.2495714
plate 19	G06	hsa-miR-4430	MIMAT0018945	AGGCUGGAGUGAGCGGAG	26.83336432
plate 19	G07	hsa-miR-4706	MIMAT0019806	AGCGGGGAGGAAGUGGGCGCUGCUU	17.89732136
plate 19	G08	hsa-miR-6752-3p	MIMAT0027405	UCCCUGCCCCCAUACUCCCAG	-42.15747818
plate 19	G09	hsa-miR-381-5p	MIMAT0022862	AGCGAGGUUGCCCUUUGUAUAU	20.59041969
plate 19	G10	hsa-miR-548ae	MIMAT0018954	CAAAAACUGCAAUUACUUUCA	8.595948139

plate	19	G11	hsa-miR-3605-3p	MIMAT0017982	CCUCCGUGUUACCUGUCCUCUAG	-19.80443874
plate	19	G12				
plate	19	H01				
plate	19	H02				
plate	19	H03				
plate	19	H04				
plate	19	H05				
plate	19	H06				
plate	19	H07				
plate	19	H08				
plate	19	H09				
plate	19	H10				
plate	19	H11				
plate	19	H12				
plate	20	A01				
plate	20	A02				
plate	20	A03				
plate	20	A04				
plate	20	A05				
plate	20	A06				
plate	20	A07				
plate	20	A08				
plate	20	A09				
plate	20	A10				
plate	20	A11				
plate	20	A12				
plate	20	B01				
plate	20	B02				
plate	20	B03				
plate	20	B04	hsa-miR-3912-3p	MIMAT0018186	UAACGCAUAAUAUGGACAUGU	37.40250875
plate	20	B05	hsa-miR-573	MIMAT0003238	CUGAAGUGAUGUGUAACUGAUCAG	28.66367087
plate	20	B06	hsa-miR-6777-5p	MIMAT0027454	ACGGGGAGUCAGGCAGUGGUGGA	22.11894579
plate	20	B07	hsa-miR-3149	MIMAT0015022	UUUGUAUGGAUAUGUGUGUGUAU	28.44336403
plate	20	B08	hsa-miR-4491	MIMAT0019026	AAUGUGGACUGGUGUGACCAAA	-2.655722306
plate	20	B09	hsa-miR-3183	MIMAT0015063	GCCUCUCUCGGAGUCGCUCGGA	28.5830708
plate	20	B10	hsa-miR-4419b	MIMAT0019034	GAGGCUGAAGGAAGAUGG	-1.348210181
plate	20	B11	hsa-miR-6499-5p	MIMAT0025450	UCGGGCGCAAGAGCACUGCAGU	-22.16810188
plate	20	B12				
plate	20	C01				
plate	20	C02	hsa-miR-3160-3p	MIMAT0015034	AGAGCUGAGACUAGAAAGCCCA	-64.95957061
plate	20	C03	hsa-miR-4662a-5p	MIMAT0019731	UUAGCCAAUUGUCCAUCUUUAG	39.39243474
plate	20	C04	hsa-miR-4742-3p	MIMAT0019873	UCUGUAUUCUCCUUUGCCUGCAG	-87.14608115
plate	20	C05	hsa-miR-5010-3p	MIMAT0021044	UUUUGUGUCUCCCAUUCCCCAG	-27.04888344
plate	20	C06	hsa-miR-6884-5p	MIMAT0027668	AGAGGCUGAGAAGGUGAUGUUG	-3.603220818
plate	20	C07	hsa-miR-3174	MIMAT0015051	UAGUGAGUUAGAGAUGCAGAGCC	-1.779868294
plate	20	C08	hsa-miR-3188	MIMAT0015070	AGAGGCUUUGUGCGGAUACGGGG	14.26312637
plate	20	C09	hsa-miR-6516-5p	MIMAT0030417	UUUGCAGUAACAGGUGUGAGCA	-60.79702516
plate	20	C10	hsa-miR-4723-5p	MIMAT0019838	UGGGGGAGCCAUGAGAUAAGAGCA	-2.019877368
plate	20	C11	hsa-miR-7706	MIMAT0030021	UGAAGCGCCUGUGCUCUGCCGAGA	24.81994344

plate	20	C12				
plate	20	D01				
plate	20	D02	hsa-miR-3661	MIMAT0018082	UGACCUGGGACUCGGACAGCUG	9.176366868
plate	20	D03	hsa-miR-3936	MIMAT0018351	UAAGGGGUGUAUGGCAGAUGCA	-0.832369781
plate	20	D04	hsa-miR-4422	MIMAT0018935	AAAAGCAUCAGGAAGUACCCA	-36.26773948
plate	20	D05	hsa-miR-4767	MIMAT0019919	CGCGGGCGCUCCUGGCCGCCGCC	52.87413852
plate	20	D06	hsa-miR-5196-5p	MIMAT0021128	AGGGAAGGGGACGAGGGUUGGG	18.87524105
plate	20	D07	hsa-miR-6125	MIMAT0024598	GCGGAAGGCGGAGCGGCGGA	11.68213325
plate	20	D08	hsa-miR-8072	MIMAT0030999	GGCGGCGGGGAGGUAGGCAG	23.49093797
plate	20	D09	hsa-miR-376b-5p	MIMAT0022923	CGUGGAUAUUCCUUCUAUGUUU	31.87513558
plate	20	D10	hsa-miR-4301	MIMAT0016850	UCCCACUACUUCACUUGUGA	8.71963318
plate	20	D11	hsa-miR-4449	MIMAT0018968	CGUCCCGGGGCUGCGCGAGGCA	22.5219461
plate	20	D12				
plate	20	E01				
plate	20	E02	hsa-miR-4725-3p	MIMAT0019844	UGGGGAAGGCGUCAGUGUCGGG	18.40238735
plate	20	E03	hsa-miR-6510-5p	MIMAT0025476	CAGCAGGGGAGAGAGAGGAGUC	28.46127515
plate	20	E04	hsa-miR-676-3p	MIMAT0018204	CUGUCCUAAGGUUGUUGAGUU	30.35268995
plate	20	E05	hsa-miR-3665	MIMAT0018087	AGCAGGUGCGGGGCGGCG	16.92830177
plate	20	E06	hsa-miR-4689	MIMAT0019778	UUGAGGAGACAUGGUGGGGGCC	30.09476975
plate	20	E07	hsa-miR-4785	MIMAT0019949	AGAGUCGGCGACGCCGCCAGC	32.57008723
plate	20	E08	hsa-miR-1273h-3p	MIMAT0030416	CUGCAGACUCGACCUCCCAGGC	-65.75661567
plate	20	E09	hsa-miR-3621	MIMAT0018002	CGCGGGUCGGGGUCUGCAGG	35.75647636
plate	20	E10	hsa-miR-3926	MIMAT0018201	UGGCCAAAAAGCAGGCAGAGA	16.32648797
plate	20	E11	hsa-miR-4512	MIMAT0019049	CAGGGCCUCACUGUAUCGCCCA	14.30969529
plate	20	E12				
plate	20	F01				
plate	20	F02	hsa-miR-4708-3p	MIMAT0019810	AGCAAGGCGGCAUCUCUCUGAU	-16.6837154
plate	20	F03	hsa-miR-4781-3p	MIMAT0019943	AAUGUUGGAAUCCUCGCUAGAG	17.9940137
plate	20	F04	hsa-miR-5684	MIMAT0022473	AACUCUAGCCUGAGCAACAG	-77.84841616
plate	20	F05	hsa-miR-6124	MIMAT0024597	GGGAAAAGGAAGGGGGAGGA	-30.97321093
plate	20	F06	hsa-miR-134-3p	MIMAT0026481	CCUGUGGGCCACCUAGUCACCAA	22.78523964
plate	20	F07	hsa-miR-4442	MIMAT0018960	GCCGGACAAGAGGGAGG	23.12017768
plate	20	F08	hsa-miR-6740-5p	MIMAT0027381	AGUUUGGGAUGGAGAGAGGAGA	33.15578101
plate	20	F09	hsa-miR-4477b	MIMAT0019005	AUUAAGGACAUUUGUGAUUGAU	-6.923943393
plate	20	F10	hsa-miR-3125	MIMAT0014988	UAGAGGAAGCUGUGGAGAGA	-25.75032688
plate	20	F11	hsa-miR-4772-3p	MIMAT0019927	CCUGCAACUUUGCCUGAUCAGA	-65.31779311
plate	20	F12				
plate	20	G01				
plate	20	G02	hsa-miR-548aq-3p	MIMAT0022264	CAAAAACUGCAAUUACUUUUGC	-23.73532532
plate	20	G03	hsa-miR-2467-3p	MIMAT0019953	AGCAGAGGCAGAGAGGCUCAGG	-35.44561884
plate	20	G04	hsa-miR-3116	MIMAT0014978	UGCCUGGAACAUAGUAGGGACU	-13.29313944
plate	20	G05	hsa-miR-3911	MIMAT0018185	UGUGUGGAUCCUGGAGGAGGCA	-24.84044173
plate	20	G06	hsa-miR-4539	MIMAT0019082	GCUGAACUGGGCUGAGCUGGGC	-29.25195182
plate	20	G07	hsa-miR-4709-3p	MIMAT0019812	UUGAAGAGGAGGUGCUCUGUAGC	-77.06749111
plate	20	G08	hsa-miR-4742-5p	MIMAT0019872	UCAGGCAAAGGGAUAUUUACAGA	-45.53495555
plate	20	G09	hsa-miR-556-5p	MIMAT0003220	GAUGAGCUCAUUGUAAUAUGAG	32.63635839
plate	20	G10	hsa-miR-585-5p	MIMAT0026618	CUAGCACACAGAUACGCCCAGA	-39.90190674
plate	20	G11	hsa-miR-653-5p	MIMAT0003328	GUGUUGAAACAAUCUCUACUG	9.015166743
plate	20	G12				

plate	20	H01				
plate	20	H02				
plate	20	H03				
plate	20	H04				
plate	20	H05				
plate	20	H06				
plate	20	H07				
plate	20	H08				
plate	20	H09				
plate	20	H10				
plate	20	H11				
plate	20	H12				
plate	21	A01				
plate	21	A02				
plate	21	A03				
plate	21	A04				
plate	21	A05				
plate	21	A06				
plate	21	A07				
plate	21	A08				
plate	21	A09				
plate	21	A10				
plate	21	A11				
plate	21	A12				
plate	21	B01				
plate	21	B02				
plate	21	B03				
plate	21	B04	hsa-miR-5701	MIMAT0022494	UUAUUGUCACGUUCUGAUU	6.519877104
plate	21	B05	hsa-miR-1281	MIMAT0005939	UCGCCUCCUCCUCUCCC	-11.11562601
plate	21	B06	hsa-miR-3607-3p	MIMAT0017985	ACUGUAAACGCUUUCUGAUG	25.02623678
plate	21	B07	hsa-miR-4677-3p	MIMAT0019761	UCUGUGAGACCAAAGAACUACU	-33.63292475
plate	21	B08	hsa-miR-6789-5p	MIMAT0027478	GUAGGGGCGUCCCGGGCGCGCGGG	9.121922842
plate	21	B09	hsa-miR-3128	MIMAT0014991	UCUGGCAAGUAAAAAACUCUCAU	-0.041803454
plate	21	B10	hsa-miR-4536-5p	MIMAT0019078	UGUGGUAGAUAUAUGCACGAU	10.23746116
plate	21	B11	hsa-miR-4646-5p	MIMAT0019707	ACUGGGAAGAGGAGCUGAGGGA	-32.88046259
plate	21	B12				
plate	21	C01				
plate	21	C02	hsa-miR-4732-5p	MIMAT0019855	UGUAGAGCAGGGAGCAGGAAGCU	-36.95586081
plate	21	C03	hsa-miR-6512-5p	MIMAT0025480	UACCAUUAGAAGAGCUGGAAGA	2.13928443
plate	21	C04	hsa-miR-626	MIMAT0003295	AGCUGUCUGAAAAUGUCUU	5.224116208
plate	21	C05	hsa-miR-1909-3p	MIMAT0007883	CGCAGGGGCCGGGUGCUCACCG	-43.40046247
plate	21	C06	hsa-miR-4518	MIMAT0019055	GCUCAGGGAUGAUAACUGUGCUGAGA	-36.06658735
plate	21	C07	hsa-miR-6516-3p	MIMAT0030418	AUCAUGUAUGAUACUGCAAACA	2.106397098
plate	21	C08	hsa-miR-219b-5p	MIMAT0019747	AGAUGUCCAGCCACAAUUCUCG	-6.132537411
plate	21	C09	hsa-miR-511-3p	MIMAT0026606	AAUGUGUAGCAAAAGACAGA	11.13331209
plate	21	C10	hsa-miR-548aw	MIMAT0022471	GUGCAAAAGUCAUCACGGUU	19.16045218
plate	21	C11	hsa-miR-1287-3p	MIMAT0026738	CUCUAGCCACAGAUGCAGUGAU	-47.60872552
plate	21	C12				
plate	21	D01				

plate	21	D02	hsa-miR-3134	MIMAT0015000	UGAUGGAUAAAAGACUACAUAUU	-1.4257024
plate	21	D03	hsa-miR-3915	MIMAT0018189	UUGAGGAAAAGAUGGUCUUAUU	6.679051792
plate	21	D04	hsa-miR-4691-3p	MIMAT0019782	CCAGCCACGGACUGAGAGUGCAU	7.69198163
plate	21	D05	hsa-miR-6513-5p	MIMAT0025482	UUUGGGAUUGACGCCACAUGUCU	-49.93977964
plate	21	D06	hsa-miR-548ax	MIMAT0022474	AGAAGUAAUUGCGGUUUUGCCA	-2.109758914
plate	21	D07	hsa-miR-550b-2-5p	MIMAT0022737	AUGUGCCUGAGGGAGUAAGACA	-31.16505933
plate	21	D08	hsa-miR-2392	MIMAT0019043	UAGGAUGGGGGUGAGAGGUG	4.321687807
plate	21	D09	hsa-miR-3064-5p	MIMAT0019864	UCUGGCUGUUGUGGUGUGCAA	0.808005215
plate	21	D10	hsa-miR-4636	MIMAT0019693	AACUCGUGUUCAAAGCCUUUAG	2.620754976
plate	21	D11	hsa-miR-4738-3p	MIMAT0019867	UGAAACUGGAGCGCCUGGAGGA	6.243623512
plate	21	D12				
plate	21	E01				
plate	21	E02	hsa-miR-5188	MIMAT0021119	AAUCGGACCCAUUUAAACCGGAG	7.322328014
plate	21	E03	hsa-miR-6880-5p	MIMAT0027660	UGGUGGAGGAAGAGGGCAGCUC	-15.57383279
plate	21	E04	hsa-miR-5193	MIMAT0021124	UCCUCCUCUACCUCAUCCCAGU	-6.022035974
plate	21	E05	hsa-miR-3688-3p	MIMAT0018116	UAUGGAAAGACUUUGCCACUCU	-57.40520409
plate	21	E06	hsa-miR-5001-3p	MIMAT0021022	UUCUGCCUCUGUCCAGGUCCUU	-14.70297623
plate	21	E07	hsa-miR-548al	MIMAT0019024	AACGGCAAUGACUUUUGUACCA	-61.23986705
plate	21	E08	hsa-miR-1296-3p	MIMAT0026637	GAGUGGGGCUUCGACCCUAACC	9.815187809
plate	21	E09	hsa-miR-4657	MIMAT0019724	AAUGUGGAAGUGGUCUGAGGCAU	3.700774972
plate	21	E10	hsa-miR-4668-5p	MIMAT0019745	AGGGAAAAAAAAAAGGAUUUGUC	-1.494108052
plate	21	E11	hsa-miR-597-3p	MIMAT0026619	UGGUUCUCUUGUGGCUCAAGCGU	-18.02591229
plate	21	E12				
plate	21	F01				
plate	21	F02	hsa-miR-548v	MIMAT0015020	AGCUACAGUUACUUUUGCACCA	-6.423261429
plate	21	F03	hsa-miR-4644	MIMAT0019704	UGGAGAGAGAAAAGAGACAGAAG	-32.13457789
plate	21	F04	hsa-miR-4707-5p	MIMAT0019807	GCCCCGGCGCGGGCGGGUUCUGG	7.035550475
plate	21	F05	hsa-miR-5582-3p	MIMAT0022280	UAAAACUUUAAGUGUGCCUAGG	12.49879413
plate	21	F06	hsa-miR-95-5p	MIMAT0026473	UCAAUAAAUGUCUGUUGAAUU	18.71581545
plate	21	F07	hsa-miR-3139	MIMAT0015007	UAGGAGCUCAACAGAUGCCUGUU	-47.44691985
plate	21	F08	hsa-miR-3156-5p	MIMAT0015030	AAAGAUCUGGAAGUGGGAGACA	-18.26796306
plate	21	F09	hsa-miR-4496	MIMAT0019031	GAGGAAACUGAAGCUGAGAGGG	-4.831514542
plate	21	F10	hsa-miR-4748	MIMAT0019884	GAGGUUUGGGGAGGAUUUGCU	-6.29565858
plate	21	F11	hsa-miR-6501-3p	MIMAT0025459	CCAGAGCAGCCUGCGGUAACAGU	6.490936251
plate	21	F12				
plate	21	G01				
plate	21	G02	hsa-miR-6507-5p	MIMAT0025470	GAAGAAUAGGAGGGACUUUGU	-2.382066026
plate	21	G03	hsa-miR-4431	MIMAT0018947	GCGACUCUGAAAACUAGAAGGU	-31.41631855
plate	21	G04	hsa-miR-4464	MIMAT0018988	AAGGUUUGGAUAGAUGCAAUA	6.127860102
plate	21	G05	hsa-miR-181d-3p	MIMAT0026608	CCACCGGGGGAUGAAUGUCAC	-14.945027
plate	21	G06	hsa-miR-548az-5p	MIMAT0025456	CAAAAGUGAUUGUGGUUUUUGC	10.6400021
plate	21	G07	hsa-miR-3173-5p	MIMAT0019214	UGCCCUGCCUGUUUUCUCCUUU	-26.70685255
plate	21	G08	hsa-miR-4640-5p	MIMAT0019699	UGGGCCAGGGAGCAGCUGGUGGG	8.740429786
plate	21	G09	hsa-miR-6859-5p	MIMAT0027618	GAGAGGAACAUGGGCUCAGGACA	-52.19190416
plate	21	G10	hsa-miR-7110-5p	MIMAT0028117	UGGGGGUGUGGGGAGAGAGAG	5.962107946
plate	21	G11	hsa-miR-6837-5p	MIMAT0027576	ACCAGGGCCAGCAGGGAAUGU	-34.69978981
plate	21	G12				
plate	21	H01				
plate	21	H02				

plate	21	H03				
plate	21	H04				
plate	21	H05				
plate	21	H06				
plate	21	H07				
plate	21	H08				
plate	21	H09				
plate	21	H10				
plate	21	H11				
plate	21	H12				
plate	22	A01				
plate	22	A02				
plate	22	A03				
plate	22	A04				
plate	22	A05				
plate	22	A06				
plate	22	A07				
plate	22	A08				
plate	22	A09				
plate	22	A10				
plate	22	A11				
plate	22	A12				
plate	22	B01				
plate	22	B02				
plate	22	B03				
plate	22	B04	hsa-miR-5009-5p	MIMAT0021041	UUGGACUUUUUCAGAUUUGGGGAU	9.059137047
plate	22	B05	hsa-miR-1208	MIMAT0005873	UCACUGUUCAGACAGGCGGA	9.4077492
plate	22	B06	hsa-miR-3074-5p	MIMAT0019208	GUUCCUGCUGAACUGAGCCAG	-66.53084375
plate	22	B07	hsa-miR-4436b-3p	MIMAT0019941	CAGGGCAGGAAGAAGUGGACAA	-60.86476439
plate	22	B08	hsa-miR-5588-5p	MIMAT0022295	ACUGGCAUUAGUGGGACUUUU	6.059714294
plate	22	B09	hsa-miR-5787	MIMAT0023252	GGGCUGGGGCGCGGGGAGGU	7.506228361
plate	22	B10	hsa-miR-2116-5p	MIMAT0011160	GGUUCUUAGCAUAGGAGGUCU	8.893885831
plate	22	B11	hsa-miR-2681-3p	MIMAT0013516	UAUCAUGGAGUUGGUAAAGCAC	-3.681052577
plate	22	B12				
plate	22	C01				
plate	22	C02	hsa-miR-3150a-3p	MIMAT0015023	CUGGGGAGAUCCUCGAGGUUGG	-27.06070404
plate	22	C03	hsa-miR-4632-3p	MIMAT0019688	UGCCGCCCUCUCGCUGCUCUAG	0.479656116
plate	22	C04	hsa-miR-4766-5p	MIMAT0019917	UCUGAAAGAGCAGUUGGUGUU	-13.99680313
plate	22	C05	hsa-miR-548ac	MIMAT0018938	CAAAAACCGGCAAUUACUUUUG	4.038216544
plate	22	C06	hsa-miR-6785-5p	MIMAT0027470	UGGGAGGGCGUGGAUGAUGGUG	4.586035643
plate	22	C07	hsa-miR-6850-5p	MIMAT0027600	GUGCGGAACGCUGGCCGGGGCG	-0.584290067
plate	22	C08	hsa-miR-376c-5p	MIMAT0022861	GGUGGAUAUUCCUUCUAUGUU	12.52488515
plate	22	C09	hsa-miR-548j-3p	MIMAT0026737	CAAAAACUGCAUUACUUUUGC	14.64145894
plate	22	C10	hsa-miR-3115	MIMAT0014977	AUAUGGGUUUACUAGUUGGU	3.816372447
plate	22	C11	hsa-miR-4747-5p	MIMAT0019882	AGGGAAGGAGGCUUGGUCUUAG	-21.36066895
plate	22	C12				
plate	22	D01				
plate	22	D02	hsa-miR-6747-5p	MIMAT0027394	AGGGGUGUGGAAAGAGGCAGAACA	8.420769336
plate	22	D03	hsa-miR-3660	MIMAT0018081	ACUGACAGGAGAGCAUUUUGA	-29.54173599

plate	22	D04	hsa-miR-4526	MIMAT0019065	GCUGACAGCAGGGCUGGCCGCU	-40.74939037
plate	22	D05	hsa-miR-4489	MIMAT0019023	UGGGGCUAGUGAUGCAGGACG	20.01325531
plate	22	D06	hsa-miR-548an	MIMAT0019079	AAAAGGCAUUGUGGUUUUUG	23.27753275
plate	22	D07	hsa-miR-4507	MIMAT0019044	CUGGGUUGGGCUGGGCUGGG	11.29116032
plate	22	D08	hsa-miR-943	MIMAT0004986	CUGACUGUUGCCGUCCUCCAG	2.702624526
plate	22	D09	hsa-miR-6779-5p	MIMAT0027458	CUGGGAGGGGCUGGGUUUGGC	9.04329104
plate	22	D10	hsa-miR-6806-3p	MIMAT0027513	UGAAGCUCUGACAUUCCUGCAG	-42.04876294
plate	22	D11	hsa-miR-3132	MIMAT0014997	UGGGUAGAGAAGGAGCUCAGAGGA	8.373231315
plate	22	D12				
plate	22	E01				
plate	22	E02	hsa-miR-4669	MIMAT0019749	UGUGUCCGGGAAGUGGAGGAGG	4.778451443
plate	22	E03	hsa-miR-4787-3p	MIMAT0019957	GAUGCGCCGCCCACUGCCCCGCGC	1.849203864
plate	22	E04	hsa-miR-526b-3p	MIMAT0002836	GAAAGUGCUUCCUUUUAGAGGC	32.39577792
plate	22	E05	hsa-miR-758-5p	MIMAT0022929	GAUGGUUGACCAGAGAGCACAC	18.36074315
plate	22	E06	hsa-miR-4511	MIMAT0019048	GAAGAACUGUUGCAUUUGCCCU	-19.84397971
plate	22	E07	hsa-miR-6813-5p	MIMAT0027526	CAGGGGCUGGGGUUUCAGGUUCU	6.38342558
plate	22	E08	hsa-miR-4437	MIMAT0018953	UGGGCUCAGGGUACAAAGGUU	11.49715841
plate	22	E09	hsa-miR-939-3p	MIMAT0022939	CCCUGGGCCUCUGCUCCCCAG	-52.22416315
plate	22	E10	hsa-miR-1237-5p	MIMAT0022946	CGGGGGCGGGGCCGAAGCGCG	20.1739791
plate	22	E11	hsa-miR-4536-3p	MIMAT0020959	UCGUGCAUAUAUCUACCACAU	0.411744658
plate	22	E12				
plate	22	F01				
plate	22	F02	hsa-miR-4763-3p	MIMAT0019913	AGGCAGGGGCUGGUGCUGGGCGGG	-0.921583645
plate	22	F03	hsa-miR-4789-3p	MIMAT0019960	CACACAUAGCAGGUGUAUAUA	6.768257178
plate	22	F04	hsa-miR-518d-5p	MIMAT0005456	CUCUAGAGGGAAGCACUUUCUG	-61.89022742
plate	22	F05	hsa-miR-662	MIMAT0003325	UCCCACGUUGUGGCCCAGCAG	-70.30672084
plate	22	F06	hsa-miR-323b-5p	MIMAT0001630	AGGUUGUCCGUGGUGAGUUCGCA	-2.164363336
plate	22	F07	hsa-miR-3186-3p	MIMAT0015068	UCACGCGGAGAGAUGGCUUUG	-1.007604826
plate	22	F08	hsa-miR-4470	MIMAT0018997	UGGCAAACGUGGAAGCCGAGA	-7.47730311
plate	22	F09	hsa-miR-4654	MIMAT0019720	UGUGGGAUCUGGAGGCAUCUGG	-39.63337873
plate	22	F10	hsa-miR-4685-3p	MIMAT0019772	UCUCCCUUCCUGCCCUGGCUAG	-23.09241115
plate	22	F11	hsa-miR-5006-3p	MIMAT0021034	UUUCCCUUUCCAUCCUGGCAG	-62.38598106
plate	22	F12				
plate	22	G01				
plate	22	G02	hsa-miR-5581-3p	MIMAT0022276	UUCCAUGCCUCCUAGAAGUUCC	-0.620509512
plate	22	G03	hsa-miR-1233-5p	MIMAT0022943	AGUGGGAGGCCAGGGCACGGCA	7.900114821
plate	22	G04	hsa-miR-4796-3p	MIMAT0019971	UAAAGUGGCAGAGUAUAGACAC	-24.85810907
plate	22	G05	hsa-miR-3181	MIMAT0015061	AUCGGGCCCUCGGCGCCGG	26.72743485
plate	22	G06	hsa-miR-6820-5p	MIMAT0027540	UGCGGCAGAGCUGGGGUCA	26.07774856
plate	22	G07	hsa-miR-548ar-3p	MIMAT0022266	UAAAACUGCAGUUAUUUUUGC	6.777312039
plate	22	G08	hsa-miR-4687-5p	MIMAT0019774	CAGCCCUCCUCCCGCACCCAAA	-33.48060059
plate	22	G09	hsa-miR-6501-5p	MIMAT0025458	AGUUGCCAGGGCUGCCUUUGGU	-52.28301975
plate	22	G10	hsa-miR-5189-5p	MIMAT0021120	UCUGGGCACAGGCGGAUGGACAGG	-59.06963817
plate	22	G11	hsa-miR-5706	MIMAT0022500	UUCUGGAUAACAUGCUGAAGCU	-36.33967299
plate	22	G12				
plate	22	H01				
plate	22	H02				
plate	22	H03				
plate	22	H04				

plate	22	H05				
plate	22	H06				
plate	22	H07				
plate	22	H08				
plate	22	H09				
plate	22	H10				
plate	22	H11				
plate	22	H12				
plate	23	A01				
plate	23	A02				
plate	23	A03				
plate	23	A04				
plate	23	A05				
plate	23	A06				
plate	23	A07				
plate	23	A08				
plate	23	A09				
plate	23	A10				
plate	23	A11				
plate	23	A12				
plate	23	B01				
plate	23	B02				
plate	23	B03				
plate	23	B04	hsa-miR-328-5p	MIMAT0026486	GGGGGGGCAGGAGGGGCUCAGGG	3.681565555
plate	23	B05	hsa-miR-374c-3p	MIMAT0022735	CACUUAGCAGGUUGUAUUAUAU	20.17274193
plate	23	B06	hsa-miR-3651	MIMAT0018071	CAUAGCCCGGUCGCUGGUACAUGA	17.92799906
plate	23	B07	hsa-miR-4665-5p	MIMAT0019739	CUGGGGGACGCGUGAGCGCGAGC	23.69353056
plate	23	B08	hsa-miR-4728-5p	MIMAT0019849	UGGGAGGGGAGAGGCAGCAAGCA	12.99258335
plate	23	B09	hsa-miR-4772-5p	MIMAT0019926	UGAUCAGGCAAAAUUGCAGACU	-14.91851655
plate	23	B10	hsa-miR-4446-3p	MIMAT0018965	CAGGGCUGGCAGUGACAUGGGU	26.11664542
plate	23	B11	hsa-miR-4476	MIMAT0019003	CAGGAAGGAUUUAGGGACAGGC	16.19367449
plate	23	B12				
plate	23	C01				
plate	23	C02	hsa-miR-6131	MIMAT0024615	GGCUGGUCAGAUGGGAGUG	3.423612218
plate	23	C03	hsa-miR-6842-3p	MIMAT0027587	UUGGCUGGUCUCUGCUCCGCAG	-39.97017628
plate	23	C04	hsa-miR-3146	MIMAT0015018	CAUGCUAGGAUAGAAAGAAUGG	-3.542499974
plate	23	C05	hsa-miR-4498	MIMAT0019033	UGGGCUGGCAGGGCAAGUGCUG	17.25704596
plate	23	C06	hsa-miR-6862-5p	MIMAT0027625	CGGGCAUGCUGGGAGAGACUUU	13.93932698
plate	23	C07	hsa-miR-503-3p	MIMAT0022925	GGGGUAUUGUUUCCGCUGCCAGG	26.21543606
plate	23	C08	hsa-miR-3622a-3p	MIMAT0018004	UCACCUGACCUCCCAUGCCUGU	-3.892383489
plate	23	C09	hsa-miR-3940-5p	MIMAT0019229	GUGGGUUGGGGCGGGCUCUG	18.85278921
plate	23	C10	hsa-miR-4633-3p	MIMAT0019690	AGGAGCUAGCCAGGCAUAUGCA	-12.41307616
plate	23	C11	hsa-miR-4634	MIMAT0019691	CGGCGCGACCGGCCCGGGG	50.09807321
plate	23	C12				
plate	23	D01				
plate	23	D02	hsa-miR-5703	MIMAT0022496	AGGAGAAGUCGGGAAGGU	9.934189794
plate	23	D03	hsa-miR-6513-3p	MIMAT0025483	UCAAGUGUCAUCUGUCCCUAG	15.69697711
plate	23	D04	hsa-miR-3682-3p	MIMAT0018110	UGAUGAUACAGGUGGAGGUAG	-11.16584434
plate	23	D05	hsa-miR-4515	MIMAT0019052	AGGACUGGACUCCCGGCAGCCC	-9.987217123

plate	23	D06	hsa-miR-6775-5p	MIMAT0027450	UCGGGGCAUGGGGGAGGGAGGCUGG	11.20749137
plate	23	D07	hsa-miR-1538	MIMAT0007400	CGGCCCGGGCUGCUGCUGUUCCU	22.2926244
plate	23	D08	hsa-miR-6090	MIMAT0023715	GGGGAGCGAGGGGCGGGGC	25.65973871
plate	23	D09	hsa-miR-7110-3p	MIMAT0028118	UCUCUCUCCCACUUCCCUGCAG	-22.41425634
plate	23	D10	hsa-miR-937-3p	MIMAT0004980	AUCCGCGCUCUGACUCUCUGCC	8.636190555
plate	23	D11	hsa-miR-3194-5p	MIMAT0015078	GGCCAGCCACCAGGAGGGCUG	9.021748469
plate	23	D12				
plate	23	E01				
plate	23	E02	hsa-miR-6511b-5p	MIMAT0025847	CUGCAGGCAGAAGUGGGGCUGACA	15.03288448
plate	23	E03	hsa-miR-7845-5p	MIMAT0030420	AAGGGACAGGGAGGGUCGUGG	8.201237322
plate	23	E04	hsa-miR-541-5p	MIMAT0004919	AAAGGAUUCUGCUGUCGGUCCCACU	13.05158331
plate	23	E05	hsa-miR-2114-5p	MIMAT0011156	UAGUCCCUUCCUUGAAGCGGUC	15.19753554
plate	23	E06	hsa-miR-3622b-3p	MIMAT0018006	UCACCUGAGCUCCCGUGCCUG	-35.67552763
plate	23	E07	hsa-miR-3652	MIMAT0018072	CGGCUGGAGGUGUGAGGA	13.62923414
plate	23	E08	hsa-miR-6729-5p	MIMAT0027359	UGGGCGAGGGCGGCUGAGCGGC	6.118401335
plate	23	E09	hsa-miR-6894-5p	MIMAT0027688	AGGAGGAUGGAGAGCUGGGCCAGA	-4.527662186
plate	23	E10	hsa-miR-7846-3p	MIMAT0030421	CAGCGGAGCCUGGAGAGAAGG	13.41793194
plate	23	E11	hsa-miR-875-3p	MIMAT0004923	CCUGGAAACACUGAGGUUGUG	11.21846811
plate	23	E12				
plate	23	F01				
plate	23	F02	hsa-miR-3158-5p	MIMAT0019211	CCUGCAGAGAGGAAGCCCUUC	-55.53930668
plate	23	F03	hsa-miR-4463	MIMAT0018987	GAGACUGGGGUGGGGCC	9.304399466
plate	23	F04	hsa-miR-4775	MIMAT0019931	UUAAUUUUUUGUUUCGGUCACU	12.14051408
plate	23	F05	hsa-miR-5689	MIMAT0022481	AGCAUACACCUGUAGUCCUAGA	-16.08342285
plate	23	F06	hsa-miR-1180-5p	MIMAT0026735	GGACCCACCCGGCCGGGAAUA	16.13193034
plate	23	F07	hsa-miR-4426	MIMAT0018941	GAAGAUGGACGUACUUU	17.6563248
plate	23	F08	hsa-miR-4645-3p	MIMAT0019706	AGACAGUAGUUCUUGCCUGGUU	7.561842349
plate	23	F09	hsa-miR-1322	MIMAT0005953	GAUGAUGCUGCUGAUGCUG	15.34023313
plate	23	F10	hsa-miR-4436a	MIMAT0018952	GCAGGACAGGCAGAAGUGGAU	-6.39782388
plate	23	F11	hsa-miR-3619-5p	MIMAT0017999	UCAGCAGGCAGGCUGGUGCAGC	14.9574194
plate	23	F12				
plate	23	G01				
plate	23	G02	hsa-miR-4786-5p	MIMAT0019954	UGAGACCAGGACUGGAUGCACC	-24.60685971
plate	23	G03	hsa-miR-6761-5p	MIMAT0027422	UCUGAGAGAGCUCGAUGGCAG	-9.44112442
plate	23	G04	hsa-miR-3118	MIMAT0014980	UGUGACUGCAUUAUGAAAAUUCU	-10.33298436
plate	23	G05	hsa-miR-548as-5p	MIMAT0022267	AAAAGUAAUUGCGGGUUUUGCC	16.74662766
plate	23	G06	hsa-miR-3913-3p	MIMAT0019225	AGACAUCAAGAUCAGUCCCAAA	-4.438476192
plate	23	G07	hsa-miR-4420	MIMAT0018933	GUCACUGAUGUCUGUAGCUGAG	1.800427124
plate	23	G08	hsa-miR-3165	MIMAT0015039	AGGUGGAUGCAAUGUGACCUCA	-4.176406578
plate	23	G09	hsa-miR-631	MIMAT0003300	AGACCUGGCCCAGACCUCAGC	22.93064728
plate	23	G10	hsa-miR-1273g-5p	MIMAT0020602	GGUGGUUGAGGCUGCAGUAAGU	-2.066128747
plate	23	G11	hsa-miR-3197	MIMAT0015082	GGAGGCGCAGGCUCGGAAAGGCG	12.74286256
plate	23	G12				
plate	23	H01				
plate	23	H02				
plate	23	H03				
plate	23	H04				
plate	23	H05				
plate	23	H06				

plate	23	H07				
plate	23	H08				
plate	23	H09				
plate	23	H10				
plate	23	H11				
plate	23	H12				
plate	24	A01				
plate	24	A02				
plate	24	A03				
plate	24	A04				
plate	24	A05				
plate	24	A06				
plate	24	A07				
plate	24	A08				
plate	24	A09				
plate	24	A10				
plate	24	A11				
plate	24	A12				
plate	24	B01				
plate	24	B02				
plate	24	B03				
plate	24	B04	hsa-miR-7850-5p	MIMAT0030425	GUUUGGACAUAGUGUGGCUGG	4.542553201
plate	24	B05	hsa-miR-4749-5p	MIMAT0019885	UGCGGGGACAGGCCAGGGCAUC	-4.581872918
plate	24	B06	hsa-miR-3678-3p	MIMAT0018103	CUGCAGAGUUUGUACGGACCGG	13.58747381
plate	24	B07	hsa-miR-378j	MIMAT0024612	ACUGGAUUUGGAGCCAGAA	11.92409389
plate	24	B08	hsa-miR-3064-3p	MIMAT0019865	UUGCCACACUGCAACACCUUACA	-43.46630153
plate	24	B09	hsa-miR-3074-3p	MIMAT0015027	GAUAUCAGCUCAGUAGGCACCG	-56.25733456
plate	24	B10	hsa-miR-6075	MIMAT0023700	ACGGCCCAGGCGGCAUUGGUG	-1.986251956
plate	24	B11	hsa-miR-4505	MIMAT0019041	AGGCUGGGCUGGGACGGA	22.86467522
plate	24	B12				
plate	24	C01				
plate	24	C02	hsa-miR-1253	MIMAT0005904	AGAGAAGAAGAUCAGCCUGCA	-58.92310744
plate	24	C03	hsa-miR-3612	MIMAT0017989	AGGAGGCAUCUUGAGAAAUGGA	9.91619011
plate	24	C04	hsa-miR-6753-3p	MIMAT0027407	UGGUCUGUCUCUGCCCUGGCAC	18.87069269
plate	24	C05	hsa-miR-6866-5p	MIMAT0027632	UUAGAGGCUGGAAUAGAGAUUCU	-12.72417219
plate	24	C06	hsa-miR-2277-3p	MIMAT0011777	UGACAGCGCCCUGCCUGGCUC	11.99892261
plate	24	C07	hsa-miR-4270	MIMAT0016900	UCAGGGAGUCAGGGGAGGGC	7.842811204
plate	24	C08	hsa-miR-4473	MIMAT0019000	CUAGUGCUCUCCGUUACAAGUA	20.43741886
plate	24	C09	hsa-miR-6088	MIMAT0023713	AGAGAUGAAGCGGGGGGGCG	14.01773891
plate	24	C10	hsa-miR-6817-3p	MIMAT0027535	UCUCUCUGACUCCAUGGCA	-54.17771992
plate	24	C11	hsa-miR-504-3p	MIMAT0026612	GGGAGUGCAGGGCAGGGUUUC	12.25146951
plate	24	C12				
plate	24	D01				
plate	24	D02	hsa-miR-4440	MIMAT0018958	UGUCGUGGGGCUUGCUGGCUUG	6.199697394
plate	24	D03	hsa-miR-4433-3p	MIMAT0018949	ACAGGAGUGGGGGUGGGACAU	12.45568954
plate	24	D04	hsa-miR-4778-5p	MIMAT0019936	AAUUCUGUAAAGGAAGAAGAGG	8.226308355
plate	24	D05	hsa-miR-4524a-5p	MIMAT0019062	AUAGCAGCAUGAACCUGUCUCA	-21.64437828
plate	24	D06	hsa-miR-1288-3p	MIMAT0005942	UGGACUGCCCUGAUCUGGAGA	-17.61765819
plate	24	D07	hsa-miR-4685-5p	MIMAT0019771	CCCAGGGCUUGGAGUGGGGCAAGGUU	9.805505973

plate	24	D08	hsa-miR-5698	MIMAT0022491	UGGGGGAGUGCAGUGAUUGUGG	17.71240658
plate	24	D09	hsa-miR-4520a-3p	MIMAT0019057	UUGGACAGAAAACACGCAGGAA	-57.82717859
plate	24	D10	hsa-miR-4731-5p	MIMAT0019853	UGCUGGGGGCCACAUGAGUGUG	12.23588019
plate	24	D11	hsa-miR-3155a	MIMAT0015029	CCAGGCUCUGCAGUGGGAACU	2.975827035
plate	24	D12				
plate	24	E01				
plate	24	E02	hsa-miR-3155a	MIMAT0015029	CCAGGCUCUGCAGUGGGAACU	-13.77956882
plate	24	E03	hsa-miR-4638-3p	MIMAT0019696	CCUGGACACCGCUCAGCCGGCCG	-46.11804403
plate	24	E04	hsa-miR-4734	MIMAT0019859	GCUGCGGGCUGCGGUCAGGGCG	15.88689779
plate	24	E05	hsa-miR-5004-5p	MIMAT0021027	UGAGGACAGGGCAAAUUCACGA	-47.97317252
plate	24	E06	hsa-miR-6891-5p	MIMAT0027682	UAAGGAGGGGGAUGAGGGG	15.07313554
plate	24	E07	hsa-miR-4769-5p	MIMAT0019922	GGUGGGAUGGAGAGAAGGUAUGAG	27.17979763
plate	24	E08	hsa-miR-1587	MIMAT0019077	UUGGGCUGGGCUGGGUUGGG	11.06512263
plate	24	E09	hsa-miR-4660	MIMAT0019728	UGCAGCUCUGGUGGAAAAUGGAG	-15.55830967
plate	24	E10	hsa-miR-4705	MIMAT0019805	UCAAUCACUUGGUAAUUGCUGU	12.6318488
plate	24	E11	hsa-miR-564	MIMAT0003228	AGGCACGGUGUCAGCAGGC	20.56369231
plate	24	E12				
plate	24	F01				
plate	24	F02	hsa-miR-1910-5p	MIMAT0007884	CCAGUCCUGUGCCUGCCGCCU	13.01222809
plate	24	F03	hsa-miR-3191-3p	MIMAT0015075	UGGGGACGUAGCUGGCCAGACAG	14.70834557
plate	24	F04	hsa-miR-5695	MIMAT0022488	ACUCCAAGAAGAAUCUAGACAG	-48.45644129
plate	24	F05	hsa-miR-6085	MIMAT0023710	AAGGGGCUGGGGGAGCACA	15.61720264
plate	24	F06	hsa-miR-6086	MIMAT0023711	GGAGGUUGGGAAGGGCAGAG	11.44238406
plate	24	F07	hsa-miR-770-5p	MIMAT0003948	UCCAGUACCACGUGUCAGGGCCA	30.20724262
plate	24	F08	hsa-miR-1182	MIMAT0005827	GAGGGUCUUGGGAGGGAUGUGAC	13.92887981
plate	24	F09	hsa-miR-2115-5p	MIMAT0011158	AGCUUCCAUGACUCCUGAUGGA	12.98728518
plate	24	F10	hsa-miR-3185	MIMAT0015065	AGAAGAAGGCGGUCGGUCUGCGG	1.971875143
plate	24	F11	hsa-miR-6799-5p	MIMAT0027498	GGGGAGGUGUGCAGGGCUGG	5.903500407
plate	24	F12				
plate	24	G01				
plate	24	G02	hsa-miR-3657	MIMAT0018077	UGUGUCCCAUUAUUGGUGAUU	-47.84066334
plate	24	G03	hsa-miR-6810-5p	MIMAT0027520	AUGGGGACAGGGAUCAGCAUGGC	-12.83329739
plate	24	G04	hsa-miR-4755-5p	MIMAT0019895	UUUCCCUUCAGAGCCUGGCUUU	-69.57684536
plate	24	G05	hsa-miR-6763-5p	MIMAT0027426	CUGGGGAGUGGCUGGGGAG	7.63235545
plate	24	G06	hsa-miR-1245b-5p	MIMAT0019950	UAGGCCUUUAGAUCACUUAAA	24.01828453
plate	24	G07	hsa-miR-5194	MIMAT0021125	UGAGGGGUUUGGAAUGGGAUGG	-16.51237575
plate	24	G08	hsa-miR-7974	MIMAT0031177	AGGCUGUGAUGCUCUCCUGAGCCC	-1.85530171
plate	24	G09	hsa-miR-1343-3p	MIMAT0019776	CUCCUGGGGCCCGCACUCUCGC	8.631630547
plate	24	G10	hsa-miR-3914	MIMAT0018188	AAGGAACCAGAAAAUGAGAAGU	10.40881247
plate	24	G11	hsa-miR-4311	MIMAT0016863	GAAAGAGAGCUGAGUGUG	9.697939699
plate	24	G12				
plate	24	H01				
plate	24	H02				
plate	24	H03				
plate	24	H04				
plate	24	H05				
plate	24	H06				
plate	24	H07				
plate	24	H08				

plate	24	H09				
plate	24	H10				
plate	24	H11				
plate	24	H12				
plate	25	A01				
plate	25	A02				
plate	25	A03				
plate	25	A04				
plate	25	A05				
plate	25	A06				
plate	25	A07				
plate	25	A08				
plate	25	A09				
plate	25	A10				
plate	25	A11				
plate	25	A12				
plate	25	B01				
plate	25	B02				
plate	25	B03				
plate	25	B04	hsa-miR-6514-3p	MIMAT0025485	CUGCCUGUUCUUCCACUCCAG	15.34065422
plate	25	B05	hsa-miR-3129-3p	MIMAT0019202	AAACUAAUCUCUACACUGCUGC	13.08606348
plate	25	B06	hsa-miR-6832-5p	MIMAT0027564	AGUAGAGAGGAAAAGUUAGGGUC	1.875079107
plate	25	B07	hsa-miR-7851-3p	MIMAT0030426	UACCUGGGAGACUGAGGUUGGA	-4.697317193
plate	25	B08	hsa-miR-5708	MIMAT0022502	AUGAGCGACUGUGCCUGACC	15.5156265
plate	25	B09	hsa-miR-6507-3p	MIMAT0025471	CAAAGUCCUUCCUAUUUUUCCC	8.108466613
plate	25	B10	hsa-miR-6840-5p	MIMAT0027582	ACCCCCGGGCAAAGACCUGCAGAU	-32.88608069
plate	25	B11	hsa-miR-6842-5p	MIMAT0027586	UGGGGGUGGUCUCUAGCCAAGG	11.53136186
plate	25	B12				
plate	25	C01				
plate	25	C02	hsa-miR-4639-5p	MIMAT0019697	UUGCUAAGUAGGCUGAGAUUGA	-22.72310738
plate	25	C03	hsa-miR-4650-5p	MIMAT0019713	UCAGGCCUCUUUCUACCUU	-16.77951771
plate	25	C04	hsa-miR-1245b-3p	MIMAT0019951	UCAGAUGAUCUAAAGGCCUAUA	-12.57289246
plate	25	C05	hsa-miR-3944-3p	MIMAT0018360	UUCGGGCUGGCCUGCUGCUCCGG	-9.82254692
plate	25	C06	hsa-miR-4469	MIMAT0018996	GCUCCCUCUAGGGUCGCUCGGA	21.77999868
plate	25	C07	hsa-miR-3170	MIMAT0015045	CUGGGGUUCUGAGACAGACAGU	-22.0851876
plate	25	C08	hsa-miR-7852-3p	MIMAT0030427	UAUGUAGUAGUCAAAGGCAUUU	3.777902662
plate	25	C09	hsa-miR-4530	MIMAT0019069	CCCAGCAGGACGGGAGCG	6.834449693
plate	25	C10	hsa-miR-548ar-5p	MIMAT0022265	AAAAGUAAUUGCAGUUUUUGC	9.459033906
plate	25	C11	hsa-miR-3187-5p	MIMAT0019216	CCUGGGCAGCGUGUGGCUGAAGG	-15.89007528
plate	25	C12				
plate	25	D01				
plate	25	D02	hsa-miR-6797-5p	MIMAT0027494	AGGAGGGAAGGGGCUGAGAACAGGA	-39.75738797
plate	25	D03	hsa-miR-3122	MIMAT0014984	GUUGGGACAAGAGGACGGUCUU	-2.987692199
plate	25	D04	hsa-miR-4506	MIMAT0019042	AAAUGGGUGGUCUGAGGCAA	-24.15569293
plate	25	D05	hsa-miR-4513	MIMAT0019050	AGACUGACGGCUGGAGGCCCAU	2.591371882
plate	25	D06	hsa-miR-4635	MIMAT0019692	UCUUGAAGUCAGAACCCGCAA	18.94581226
plate	25	D07	hsa-miR-4762-3p	MIMAT0019911	CUUCUGAUCAAGAUUUGUGGUG	1.002040331
plate	25	D08	hsa-miR-6505-5p	MIMAT0025466	UUGGAAUAGGGGAUAUCUCAGC	-40.33151577
plate	25	D09	hsa-miR-6727-5p	MIMAT0027355	CUCGGGGCAGGCGGCUGGGAGCG	-0.018631308

plate	25	D10	hsa-miR-6720-5p	MIMAT0027345	UUCCAGCCCUGGUAGGCGCCGCG	0.981991424
plate	25	D11	hsa-miR-1915-5p	MIMAT0007891	ACCUUGCCUUGCUGCCCGGGCC	-29.66185189
plate	25	D12				
plate	25	E01				
plate	25	E02	hsa-miR-2114-3p	MIMAT0011157	CGAGCCUCAAGCAAGGGACUU	-44.94276442
plate	25	E03	hsa-miR-3194-3p	MIMAT0019218	AGCUCUGCUGCUCACUGGCAGU	-31.09261481
plate	25	E04	hsa-miR-3922-3p	MIMAT0018197	UCUGGCCUUGACUUGACUCUUU	-23.99165641
plate	25	E05	hsa-miR-4523	MIMAT0019061	GACCGAGAGGGCCUCGGCUGU	14.68086291
plate	25	E06	hsa-miR-4784	MIMAT0019948	UGAGGAGAUGCUGGGACUGA	-43.51929201
plate	25	E07	hsa-miR-5699-3p	MIMAT0022492	UCCUGUCUUUCCUUGUUGGAGC	3.706820172
plate	25	E08	hsa-miR-6746-5p	MIMAT0027392	CCGGGAGAAGGAGGUGGCCUGG	5.476591888
plate	25	E09	hsa-miR-4700-5p	MIMAT0019796	UCUGGGGAUGAGGACAGUGUGU	-18.61490403
plate	25	E10	hsa-miR-3680-5p	MIMAT0018106	GACUCACUCACAGGAUUGUGCA	-24.96858498
plate	25	E11	hsa-miR-6165	MIMAT0024782	CAGCAGGAGGUGAGGGGAG	6.581104411
plate	25	E12				
plate	25	F01				
plate	25	F02	hsa-miR-6736-5p	MIMAT0027373	CUGGGUGAGGGCAUCUGUGGU	-12.92830491
plate	25	F03	hsa-miR-3150b-5p	MIMAT0019226	CAACCUCGAGGAUCUCCCCAGC	-4.547861703
plate	25	F04	hsa-miR-3171	MIMAT0015046	AGAUGUAUGGAAUCUGUAUAUAUC	3.201952237
plate	25	F05	hsa-miR-635	MIMAT0003305	ACUUGGGCACUGAAACAAUGUCC	-30.55493957
plate	25	F06	hsa-miR-593-3p	MIMAT0004802	UGUCUCUGCUGGGGUUUCU	11.16136839
plate	25	F07	hsa-miR-519e-5p	MIMAT0002828	UUCUCCAAAAGGGAGCACUUUC	-41.69848671
plate	25	F08	hsa-miR-3659	MIMAT0018080	UGAGUGUUGUCUACGAGGGCA	4.494195436
plate	25	F09	hsa-miR-4754	MIMAT0019894	AUGCGGACCUGGGUUAGCGGAGU	5.192261932
plate	25	F10	hsa-miR-6509-5p	MIMAT0025474	AUUAGGUAGUGGCAGUGGAAC	-35.86607735
plate	25	F11	hsa-miR-891a-3p	MIMAT0026717	AGUGGCACAUGUUUGUUGUGAG	2.263298855
plate	25	F12				
plate	25	G01				
plate	25	G02	hsa-miR-937-5p	MIMAT0022938	GUGAGUCAGGGUGGGGCUGG	1.018443982
plate	25	G03	hsa-miR-5006-5p	MIMAT0021033	UUGCCAGGGCAGGAGGUGGAA	-11.49936461
plate	25	G04	hsa-miR-5739	MIMAT0023116	GCGGAGAGAGAAUGGGGAGC	23.66459596
plate	25	G05	hsa-miR-6849-5p	MIMAT0027598	GAGUGGAUAGGGGAGUGUGUGGA	4.825913719
plate	25	G06	hsa-miR-548aj-3p	MIMAT0018990	UAAAAACUGCAAUUACUUUUA	0.529979698
plate	25	G07	hsa-miR-548x-3p	MIMAT0015081	UAAAAACUGCAAUUACUUUC	-26.64540267
plate	25	G08	hsa-miR-3617-5p	MIMAT0017997	AAAGACAUAGUUGCAAGAUGGG	-5.717988831
plate	25	G09	hsa-miR-4649-5p	MIMAT0019711	UGGGCGAGGGGUGGGCUCUCAGAG	-4.666332518
plate	25	G10	hsa-miR-4659a-3p	MIMAT0019727	UUUCUUCUUAGACAUGGCAACG	6.686816831
plate	25	G11	hsa-miR-761	MIMAT0010364	GCAGCAGGGUGAAACUGACACA	-21.3014576
plate	25	G12				
plate	25	H01				
plate	25	H02				
plate	25	H03				
plate	25	H04				
plate	25	H05				
plate	25	H06				
plate	25	H07				
plate	25	H08				
plate	25	H09				
plate	25	H10				

plate	25	H11				
plate	25	H12				
plate	26	A01				
plate	26	A02				
plate	26	A03				
plate	26	A04				
plate	26	A05				
plate	26	A06				
plate	26	A07				
plate	26	A08				
plate	26	A09				
plate	26	A10				
plate	26	A11				
plate	26	A12				
plate	26	B01				
plate	26	B02				
plate	26	B03				
plate	26	B04	hsa-miR-1244	MIMAT0005896	AAGUAGUUGGUUUGUAUGAGAUGGUU	0.976675848
plate	26	B05	hsa-miR-548y	MIMAT0018354	AAAAGUAAUCACUGUUUUUGCC	-8.290704028
plate	26	B06	hsa-miR-4662a-3p	MIMAT0019732	AAAGAUAGACAAUUGGCUAAAU	5.240078247
plate	26	B07	hsa-miR-4728-3p	MIMAT0019850	CAUGCUGACCUCCCUCCUGCCCCAG	-28.96226068
plate	26	B08	hsa-miR-4755-3p	MIMAT0019896	AGCCAGGCUCUGAAGGGAAAGU	5.663021354
plate	26	B09	hsa-miR-6879-5p	MIMAT0027658	CAGGGCAGGGAAGGUGGGAGAG	-8.256732694
plate	26	B10	hsa-miR-6500-3p	MIMAT0025455	ACACUUGUUGGGAUGACCUGC	-4.367014967
plate	26	B11	hsa-miR-6778-5p	MIMAT0027456	AGUGGGAGGACAGGAGGCAGGU	-1.656102525
plate	26	B12				
plate	26	C01				
plate	26	C02	hsa-miR-6838-5p	MIMAT0027578	AAGCAGCAGUGGCAAGACUCCU	-28.44419784
plate	26	C03	hsa-miR-3529-5p	MIMAT0019828	AGGUAGACUGGGAUUUGUUGUU	0.24119647
plate	26	C04	hsa-miR-4479	MIMAT0019011	CGCGCGGCCGUGCUCGGAGCAG	14.93889406
plate	26	C05	hsa-miR-4674	MIMAT0019756	CUGGGCUCGGGACGCGCGGCU	-2.80433361
plate	26	C06	hsa-miR-6887-5p	MIMAT0027674	UGGGGGGACAGAUGGAGAGGACA	-1.156723918
plate	26	C07	hsa-miR-920	MIMAT0004970	GGGGAGCUGUGGAAGCAGUA	2.838304944
plate	26	C08	hsa-miR-3620-3p	MIMAT0018001	UCACCCUGCAUCCCGCACCCAG	-49.23805129
plate	26	C09	hsa-miR-3942-5p	MIMAT0018358	AAGCAAUACUGUUACCUGAAAU	-0.866269013
plate	26	C10	hsa-miR-6771-5p	MIMAT0027442	CUCGGGAGGGCAUGGGCCAGGC	-10.25254856
plate	26	C11	hsa-miR-3689f	MIMAT0019010	UGUGAUAUCGUGCUUCCUGGGA	-4.708426872
plate	26	C12				
plate	26	D01				
plate	26	D02	hsa-miR-1912	MIMAT0007887	UACCCAGAGCAUGCAGUGUGAA	-49.50982196
plate	26	D03	hsa-miR-3126-5p	MIMAT0014989	UGAGGGACAGAUGCCAGAAGCA	-6.530988934
plate	26	D04	hsa-miR-3186-5p	MIMAT0015067	CAGGCGUCUGUCUACGUGGCUU	12.23137875
plate	26	D05	hsa-miR-676-5p	MIMAT0018203	UCUUCAACCUCAGGACUUGCA	-41.0730412
plate	26	D06	hsa-miR-1913	MIMAT0007888	UCUGCCCCCUCCGCUGCUGCCA	3.490554554
plate	26	D07	hsa-miR-4447	MIMAT0018966	GGUGGGGGCUGUUGUUU	6.553070301
plate	26	D08	hsa-miR-4766-3p	MIMAT0019918	AUAGCAAUUGCUCUUUUGGAA	-12.41822109
plate	26	D09	hsa-miR-5579-3p	MIMAT0022270	UUAGCUUAAGGAGUACCAGAUC	-39.77193911
plate	26	D10	hsa-miR-6833-5p	MIMAT0027566	GUGUGGAAGAUGGGAGGAGAAA	-4.801848041
plate	26	D11	hsa-miR-1914-5p	MIMAT0007889	CCCUGUGCCCGGCCCACUUCUG	-58.09437803

plate	26	D12				
plate	26	E01				
plate	26	E02	hsa-miR-4529-3p	MIMAT0019068	AUUGGACUGCUGAUGGCCCGU	-17.33047597
plate	26	E03	hsa-miR-6747-3p	MIMAT0027395	UCCUGCCUUCCUCUGCACCAG	-59.67574362
plate	26	E04	hsa-miR-6751-5p	MIMAT0027402	UUGGGGGUGAGGUUGGUGUCUGG	-5.010771744
plate	26	E05	hsa-miR-1252-3p	MIMAT0026744	CAAAUGAGCUUAAUUUCCUUUU	3.155936915
plate	26	E06	hsa-miR-6758-3p	MIMAT0027417	ACUCAUUCUCCUCUGUCCAG	-54.005928
plate	26	E07	hsa-miR-2682-5p	MIMAT0013517	CAGGCAGUGACUGUUCAGACGUC	-13.37621271
plate	26	E08	hsa-miR-3137	MIMAT0015005	UCUGUAGCCUGGGAGCAAUGGGGU	-12.33329276
plate	26	E09	hsa-miR-6774-3p	MIMAT0027449	UCGUGUCCCUCUUGUCCACAG	-30.69819584
plate	26	E10	hsa-miR-6802-5p	MIMAT0027504	CUAGGUGGGGGGCUUGAAGC	-11.96810092
plate	26	E11	hsa-miR-6858-5p	MIMAT0027616	GUGAGGAGGGGCUGGCAGGGAC	-2.101126999
plate	26	E12				
plate	26	F01				
plate	26	F02	hsa-miR-875-5p	MIMAT0004922	UAUACCUCAGUUUUAUCAGGUG	-6.765391137
plate	26	F03	hsa-miR-4424	MIMAT0018939	AGAGUUAACUCAAAAUGGACUA	2.911343311
plate	26	F04	hsa-miR-4533	MIMAT0019072	UGGAAGGAGGUUGCCGGACGCU	-10.00625639
plate	26	F05	hsa-miR-4649-3p	MIMAT0019712	UCUGAGGCCUGCCUCUCCCCA	-58.6413165
plate	26	F06	hsa-miR-4722-5p	MIMAT0019836	GGCAGGAGGGCUGUGCCAGGUUG	-26.89510501
plate	26	F07	hsa-miR-4783-3p	MIMAT0019947	CCCCGGUGUUGGGGCGCGUCUGC	-2.656558308
plate	26	F08	hsa-miR-6739-5p	MIMAT0027379	UGGGAAAGAGAAAGAACAAGUA	-56.62002214
plate	26	F09	hsa-miR-6847-5p	MIMAT0027594	ACAGAGGACAGUGGAGUGUGAGC	-20.55265698
plate	26	F10	hsa-miR-495-5p	MIMAT0022924	GAAGUUGCCCAUGUUAUUUUCG	-20.76327925
plate	26	F11	hsa-miR-6751-3p	MIMAT0027403	ACUGAGCCUCUCUCUCUCCAG	-30.86295681
plate	26	F12				
plate	26	G01				
plate	26	G02	hsa-miR-6865-5p	MIMAT0027630	UAGGUGGCAGAGGAGGGACUUCA	-4.485914636
plate	26	G03	hsa-miR-6763-3p	MIMAT0027427	CUCCCCGGCCUCUGCCCCCAG	-11.22582727
plate	26	G04	hsa-miR-6811-5p	MIMAT0027522	AUGCAGGCCUGUGUACAGCACU	-5.294432381
plate	26	G05	hsa-miR-6738-5p	MIMAT0027377	CGAGGGGUAGAAGAGCACAGGGG	-1.141436818
plate	26	G06	hsa-miR-8085	MIMAT0031012	UGGGAGAGAGGACUGUGAGGC	-9.270777009
plate	26	G07	hsa-miR-548f-5p	MIMAT0026739	UGCAAAAGUAAUCACAGUUUUU	-9.340418244
plate	26	G08	hsa-miR-4468	MIMAT0018995	AGAGCAGAAGGAUGAGAU	-2.592012773
plate	26	G09	hsa-miR-4723-3p	MIMAT0019839	CCCUCUCUGGCUCCUCCCCAAA	-60.25665343
plate	26	G10	hsa-miR-4758-5p	MIMAT0019903	GUGAGUGGGAGCCGGUGGGGCUG	-20.34203471
plate	26	G11	hsa-miR-6741-5p	MIMAT0027383	GUGGGUGCUGGUGGGAGCCGUG	-7.774339753
plate	26	G12				
plate	26	H01				
plate	26	H02				
plate	26	H03				
plate	26	H04				
plate	26	H05				
plate	26	H06				
plate	26	H07				
plate	26	H08				
plate	26	H09				
plate	26	H10				
plate	26	H11				
plate	26	H12				

plate	27	A01				
plate	27	A02				
plate	27	A03				
plate	27	A04				
plate	27	A05				
plate	27	A06				
plate	27	A07				
plate	27	A08				
plate	27	A09				
plate	27	A10				
plate	27	A11				
plate	27	A12				
plate	27	B01				
plate	27	B02				
plate	27	B03				
plate	27	B04	hsa-miR-6758-5p	MIMAT0027416	UAGAGAGGGGAAGGAUGUGAUGU	-3.537264602
plate	27	B05	hsa-miR-518d-3p	MIMAT0002864	CAAAGCGCUUCCCUUUGGAGC	29.64544696
plate	27	B06	hsa-miR-4761-3p	MIMAT0019909	GAGGGCAUGCGCACUUUGUCC	6.591131276
plate	27	B07	hsa-miR-7854-3p	MIMAT0030429	UGAGGUGACCGCAGAUGGGAA	-12.56171309
plate	27	B08	hsa-miR-4776-5p	MIMAT0019932	GUGGACCAGGAUGGCAAGGGCU	8.363692397
plate	27	B09	hsa-miR-499a-3p	MIMAT0004772	AACAUCACAGCAAGUCUGUGCU	15.78457004
plate	27	B10	hsa-miR-1914-3p	MIMAT0007890	GGAGGGGUCCCGCACUGGGAGG	25.43354784
plate	27	B11	hsa-miR-4279	MIMAT0016909	CUCUCCUCCCGGCUUC	22.0078209
plate	27	B12				
plate	27	C01				
plate	27	C02	hsa-miR-4300	MIMAT0016853	UGGGAGCUGGACUACUUC	16.53951577
plate	27	C03	hsa-miR-4638-5p	MIMAT0019695	ACUCGGCUGCGGUGGACAAGU	14.54836939
plate	27	C04	hsa-miR-4658	MIMAT0019725	GUGAGUGUGGAUCCUGGAGGAAU	5.321867302
plate	27	C05	hsa-miR-4695-5p	MIMAT0019788	CAGGAGGCAGUGGGCGAGCAGG	-16.3584839
plate	27	C06	hsa-miR-5584-5p	MIMAT0022283	CAGGGAAAUGGGAAGAACUAGA	-62.37159902
plate	27	C07	hsa-miR-5680	MIMAT0022468	GAGAAAUGCUGGACUAAUCUGC	-31.36371893
plate	27	C08	hsa-miR-6502-5p	MIMAT0025460	AGCUCUAGAAAGAUUGUUGACC	-25.47844864
plate	27	C09	hsa-miR-4483	MIMAT0019017	GGGGUGGUCUGUUGUUG	25.72744398
plate	27	C10	hsa-miR-370-5p	MIMAT0026483	CAGGUCACGUCUCUGCAGUUAC	47.48310607
plate	27	C11	hsa-miR-3939	MIMAT0018355	UACGCGCAGACCACAGGAUGUC	4.937965463
plate	27	C12				
plate	27	D01				
plate	27	D02	hsa-miR-451b	MIMAT0019840	UAGCAAGAGAACCAUUACCAUU	32.16193269
plate	27	D03	hsa-miR-3678-5p	MIMAT0018102	UCCGUACAAACUCUGCUGUG	9.989305446
plate	27	D04	hsa-miR-4453	MIMAT0018975	GAGCUUGGUCUGUAGCGGUU	22.03353682
plate	27	D05	hsa-miR-4670-5p	MIMAT0019750	AAGCGACCAUGAUGUAACUUCA	-23.95937294
plate	27	D06	hsa-miR-4743-5p	MIMAT0019874	UGGCCGGAUGGGACAGGAGGCAU	19.07804371
plate	27	D07	hsa-miR-4762-5p	MIMAT0019910	CCAAAUCUUGAUCAGAAGCCU	-37.9120924
plate	27	D08	hsa-miR-4774-5p	MIMAT0019929	UCUGGUAUGUAGUAGGUAAUAA	28.13004496
plate	27	D09	hsa-miR-6132	MIMAT0024616	AGCAGGGCUGGGGAUUGCA	13.51789603
plate	27	D10	hsa-miR-6748-5p	MIMAT0027396	UGUGGGUGGGAAGGACUGGAUU	14.61816972
plate	27	D11	hsa-miR-6793-3p	MIMAT0027487	UCCCCAACCCCUGCCCGCAG	-21.71290429
plate	27	D12				
plate	27	E01				

plate	27	E02	hsa-miR-6829-5p	MIMAT0027558	UGGGCUGCUGAGAAGGGGCA	-44.28412818
plate	27	E03	hsa-miR-6848-5p	MIMAT0027596	UGGGGGCUGGGAUGGGCCAUGGU	1.034657295
plate	27	E04	hsa-miR-4457	MIMAT0018979	UCACAAGGUAUUGACUGGCGUA	31.90844727
plate	27	E05	hsa-miR-6802-3p	MIMAT0027505	UUCACCCCUCUCACCUAAGCAG	-96.13108176
plate	27	E06	hsa-miR-670-3p	MIMAT0026640	UUUCCUCAUAUUCAUUCAGGA	17.70775294
plate	27	E07	hsa-miR-3618	MIMAT0017998	UGUCUACAUUAAUGAAAAGAGC	-52.92651168
plate	27	E08	hsa-miR-4655-5p	MIMAT0019721	CACCGGGGAUGGCAGAGGGUCG	19.58501456
plate	27	E09	hsa-miR-4745-3p	MIMAT0019879	UGGCCCGGCGACGUCUCACGGUC	37.88739696
plate	27	E10	hsa-miR-5008-3p	MIMAT0021040	CCUGUGCUCCCAGGGCCUCGC	26.63301148
plate	27	E11	hsa-miR-6781-5p	MIMAT0027462	CGGGCCGGAGGUCAAGGGCGU	13.04949905
plate	27	E12				
plate	27	F01				
plate	27	F02	hsa-miR-6788-5p	MIMAT0027476	CUGGGAGAAGAGUGGUGAAGA	21.78188824
plate	27	F03	hsa-miR-6881-5p	MIMAT0027662	UGGGGUAAGGAUAGGAGGGUCA	9.868073286
plate	27	F04	hsa-miR-3144-3p	MIMAT0015015	AUAUACCUGUUCGGUCUCUUUA	8.025711831
plate	27	F05	hsa-miR-3161	MIMAT0015035	CUGAUAAGAACAGAGGCCCAGAU	-103.2690844
plate	27	F06	hsa-miR-4713-5p	MIMAT0019820	UUCUCCCACUACCAGGCUCCCA	-111.1638696
plate	27	F07	hsa-miR-4782-5p	MIMAT0019944	UUCUGGAUAUGAAGACAAUCAA	-16.57890601
plate	27	F08	hsa-miR-619-3p	MIMAT0003288	GACCUGGACAUGUUUGUGCCCAGU	-11.92799953
plate	27	F09	hsa-miR-302d-5p	MIMAT0004685	ACUUUAACAUGGAGGCACUUGC	-68.67750817
plate	27	F10	hsa-miR-1236-5p	MIMAT0022945	UGAGUGACAGGGGAAAUGGGGA	1.927366833
plate	27	F11	hsa-miR-3150a-5p	MIMAT0019206	CAACCUCGACGAUCUCCUCAGC	4.160977531
plate	27	F12				
plate	27	G01				
plate	27	G02	hsa-miR-3912-5p	MIMAT0027036	AUGUCCAUAUUAUGGGUUAGU	9.730309469
plate	27	G03	hsa-miR-3941	MIMAT0018357	UUACACACAACUGAGGAUCAUA	-47.14961559
plate	27	G04	hsa-miR-4534	MIMAT0019073	GGAUGGAGGAGGGGUCU	14.99839786
plate	27	G05	hsa-miR-4640-3p	MIMAT0019700	CACCCCCUGUUUCCUGGCCCAC	-61.83707541
plate	27	G06	hsa-miR-4726-5p	MIMAT0019845	AGGGCCAGAGGAGCCUGGAGUGG	-57.47088081
plate	27	G07	hsa-miR-5581-5p	MIMAT0022275	AGCCUUCCAGGAGAAAUGGAGA	-32.00661675
plate	27	G08	hsa-miR-5586-3p	MIMAT0022288	CAGAGUGACAAGCUGGUUAAAG	5.191450888
plate	27	G09	hsa-miR-6076	MIMAT0023701	AGCAUGACAGAGGAGAGGUGG	18.12288124
plate	27	G10	hsa-miR-6733-5p	MIMAT0027367	UGGGAAAGACAAACUCAGAGUU	-37.8073919
plate	27	G11	hsa-miR-6800-3p	MIMAT0027501	CACCUCUCCUGGCAUCGCCCC	13.20930508
plate	27	G12				
plate	27	H01				
plate	27	H02				
plate	27	H03				
plate	27	H04				
plate	27	H05				
plate	27	H06				
plate	27	H07				
plate	27	H08				
plate	27	H09				
plate	27	H10				
plate	27	H11				
plate	27	H12				
plate	28	A01				
plate	28	A02				

plate	28	A03				
plate	28	A04				
plate	28	A05				
plate	28	A06				
plate	28	A07				
plate	28	A08				
plate	28	A09				
plate	28	A10				
plate	28	A11				
plate	28	A12				
plate	28	B01				
plate	28	B02				
plate	28	B03				
plate	28	B04	hsa-miR-4450	MIMAT0018971	UGGGGAUUUGGAGAAGUGGUGA	-22.32424502
plate	28	B05	hsa-miR-1229-5p	MIMAT0022942	GUGGGUAGGGUUUGGGGGAGAGCG	4.268525768
plate	28	B06	hsa-miR-1257	MIMAT0005908	AGUGAAUGAUGGGUUCUGACC	3.361759251
plate	28	B07	hsa-miR-3617-3p	MIMAT0022966	CAUCAGCACCCUAUGUCCUUUCU	-61.14207969
plate	28	B08	hsa-miR-4499	MIMAT0019035	AAGACUGAGAGGAGGGA	0.24035693
plate	28	B09	hsa-miR-4676-5p	MIMAT0019758	GAGCCAGUGGUGAGACAGUGA	-6.790940334
plate	28	B10	hsa-miR-4690-5p	MIMAT0019779	GAGCAGGCGAGGCUGGGCUGAA	1.712781199
plate	28	B11	hsa-miR-6073	MIMAT0023698	GGUAGUGAGUUAUCAGCUAC	7.888735378
plate	28	B12				
plate	28	C01				
plate	28	C02	hsa-miR-6716-3p	MIMAT0025845	UCCGAACUCUCCAUUCCUCUGC	10.43248115
plate	28	C03	hsa-miR-6764-5p	MIMAT0027428	UCCCAGGGUCUGGUCAGAGUUG	-35.43036371
plate	28	C04	hsa-miR-6803-3p	MIMAT0027507	UCCCUCGCCUUCUCACCCUCAG	-16.97963632
plate	28	C05	hsa-miR-152-5p	MIMAT0026479	AGGUUCUGUGAUACACUCCGACU	18.98934023
plate	28	C06	hsa-miR-4764-5p	MIMAT0019914	UGGAUGUGGAAGGAGUUAUCU	-14.30904609
plate	28	C07	hsa-miR-6769b-3p	MIMAT0027621	CCCUCUCUGUCCCACCCAUAG	-22.29681919
plate	28	C08	hsa-miR-7111-5p	MIMAT0028119	UGGGGGAGGAAGGACAGGCCAU	-53.53998233
plate	28	C09	hsa-miR-510-3p	MIMAT0026613	AUUGAAACCUCUAAGAGUGGA	2.693254635
plate	28	C10	hsa-miR-1301-5p	MIMAT0026639	CGCUCUAGGCACCGCAGCA	10.73587939
plate	28	C11	hsa-miR-4637	MIMAT0019694	UACUAACUGCAGAUUCAAGUGA	3.40461211
plate	28	C12				
plate	28	D01				
plate	28	D02	hsa-miR-4683	MIMAT0019768	UGGAGAUCCAGUGCUCGCCCGAU	3.402897996
plate	28	D03	hsa-miR-548ay-3p	MIMAT0025453	CAAAACCGCGAUUACUCUUGCA	18.74079364
plate	28	D04	hsa-miR-3654	MIMAT0018074	GACUGGACAAGCUGAGGAA	-1.547464388
plate	28	D05	hsa-miR-4645-5p	MIMAT0019705	ACCAGGCAAGAAAUAUUGU	9.969670258
plate	28	D06	hsa-miR-4668-3p	MIMAT0019746	GAAAAUCCUUUUUGUUUUUCCAG	11.17983502
plate	28	D07	hsa-miR-4673	MIMAT0019755	UCCAGGCAGGAGCCGGACUGGA	4.983311472
plate	28	D08	hsa-miR-4714-5p	MIMAT0019822	AACUCUGACCCCUUAGGUUGAU	-13.2274399
plate	28	D09	hsa-miR-4781-5p	MIMAT0019942	UAGCGGGGAUUCCAAUAUUGG	10.71359591
plate	28	D10	hsa-miR-4788	MIMAT0019958	UUACGGACCAGCUAAGGGAGGC	-44.59230517
plate	28	D11	hsa-miR-4795-3p	MIMAT0019969	AUAUUAUUAGCCACUUCUGGAU	-3.818665966
plate	28	D12				
plate	28	E01				
plate	28	E02	hsa-miR-4803	MIMAT0019983	UAACAUAAUAGUGUGGAUUGA	1.729922343
plate	28	E03	hsa-miR-5190	MIMAT0021121	CCAGUGACUGAGCUGGAGCCA	-23.03388838

plate	28	E04	hsa-miR-5690	MIMAT0022482	UCAGCUACUACCUCUAUUAGG	-12.76977136
plate	28	E05	hsa-miR-5692c	MIMAT0022476	AAUAAUAUCACAGUAGGUGUAC	14.32523495
plate	28	E06	hsa-miR-6077	MIMAT0023702	GGGAAGAGCUGUACGGCCUUC	-46.61324605
plate	28	E07	hsa-miR-6718-5p	MIMAT0025849	UAGUGGUCAGAGGGCUUAUGA	-26.1192943
plate	28	E08	hsa-miR-6722-3p	MIMAT0025854	UGCAGGGGUCGGGUGGGCCAGG	2.797815613
plate	28	E09	hsa-miR-1911-5p	MIMAT0007885	UGAGUACCGCCAUGUCUGUUGGG	-18.70232129
plate	28	E10	hsa-miR-329-5p	MIMAT0026555	GAGGUUUUCUGGGUUUCUGUUUC	3.180063124
plate	28	E11	hsa-miR-4758-3p	MIMAT0019904	UGCCCCACCUGCUGACCACCCUC	-13.41942071
plate	28	E12				
plate	28	F01				
plate	28	F02	hsa-miR-4776-3p	MIMAT0019933	CUUGCCAUCCUGGUCCACUGCAU	-13.54969341
plate	28	F03	hsa-miR-6791-5p	MIMAT0027482	CCCCUGGGGCUGGGCAGGCGGA	-3.87865997
plate	28	F04	hsa-miR-6855-3p	MIMAT0027611	AGACUGACCUUCAACCCCACAG	-63.2384416
plate	28	F05	hsa-miR-7107-5p	MIMAT0028111	UCGGCCUGGGGAGGAGGAAGGG	-3.273577588
plate	28	F06	hsa-miR-7113-5p	MIMAT0028123	UCCAGGGAGACAGUGUGUGAG	-29.12070861
plate	28	F07	hsa-miR-1193	MIMAT0015049	GGGAUGGUAGACCGGUGACGUGC	-2.62392823
plate	28	F08	hsa-miR-494-5p	MIMAT0026607	AGGUUGUCCGUGUUGUCUUCUCU	0.302065049
plate	28	F09	hsa-miR-3152-3p	MIMAT0015025	UGUGUUAGAAUAGGGGCAAUAA	-39.69336622
plate	28	F10	hsa-miR-609	MIMAT0003277	AGGGUGUUUCUCUCAUCUCU	8.99605328
plate	28	F11	hsa-miR-649	MIMAT0003319	AAACCUGUGUUGUUCAAGAGUC	4.81875649
plate	28	F12				
plate	28	G01				
plate	28	G02	hsa-miR-3140-5p	MIMAT0019204	ACCUGAAUUACCAAAAGCUUU	5.4032695
plate	28	G03	hsa-miR-3152-5p	MIMAT0019207	AUUGCCUCUGUUCUAACACAAG	-2.687350463
plate	28	G04	hsa-miR-3663-5p	MIMAT0018084	GCUGGUCUGCGUGGUGCUCGG	3.121783235
plate	28	G05	hsa-miR-3944-5p	MIMAT0019231	UGUGCAGCAGGCCAACCGAGA	-63.11331125
plate	28	G06	hsa-miR-4475	MIMAT0019002	CAAGGGACCAAGCAUUCAUUAU	-1.691449997
plate	28	G07	hsa-miR-4519	MIMAT0019056	CAGCAGUGCGCAGGGCUG	-37.61414545
plate	28	G08	hsa-miR-4538	MIMAT0019081	GAGCUUGGAUGAGCUGGGCUGA	-0.131605894
plate	28	G09	hsa-miR-5697	MIMAT0022490	UCAAGUAGUUUCAUGAUAAAGG	10.08794415
plate	28	G10	hsa-miR-6759-5p	MIMAT0027418	UUGUGGGUGGGCAGAAGUCUGU	-23.357856
plate	28	G11	hsa-miR-6773-5p	MIMAT0027446	UUGGGCCCAGGAGUAAACAGGAU	-0.37158191
plate	28	G12				
plate	28	H01				
plate	28	H02				
plate	28	H03				
plate	28	H04				
plate	28	H05				
plate	28	H06				
plate	28	H07				
plate	28	H08				
plate	28	H09				
plate	28	H10				
plate	28	H11				
plate	28	H12				
plate	29	A01				
plate	29	A02				
plate	29	A03				
plate	29	A04				

plate	29	A05				
plate	29	A06				
plate	29	A07				
plate	29	A08				
plate	29	A09				
plate	29	A10				
plate	29	A11				
plate	29	A12				
plate	29	B01				
plate	29	B02				
plate	29	B03				
plate	29	B04	hsa-miR-3119	MIMAT0014981	UGGCUUUUAACUUUGAUGGC	26.45138846
plate	29	B05	hsa-miR-3189-3p	MIMAT0015071	CCCUUGGGUCUGAUGGGGUAG	7.321214758
plate	29	B06	hsa-miR-371b-5p	MIMAT0019892	ACUCAAAAGAUGGCGGCACUUU	-35.16709036
plate	29	B07	hsa-miR-432-3p	MIMAT0002815	CUGGAUGGCUCCUCCAUGUCU	-51.90093595
plate	29	B08	hsa-miR-1267	MIMAT0005921	CCUGUUGAAGUGUAAUCCCCA	-4.59246781
plate	29	B09	hsa-miR-4664-5p	MIMAT0019737	UGGGGUGCCCACUCCGCAAGUU	-25.74034655
plate	29	B10	hsa-miR-4680-3p	MIMAT0019765	UCUGAAUUGUAAGAGUUGUUA	19.01322484
plate	29	B11	hsa-miR-4732-3p	MIMAT0019856	GCCCUGACCUGUCCUGUUCUG	-51.22782826
plate	29	B12				
plate	29	C01				
plate	29	C02	hsa-miR-5579-5p	MIMAT0022269	UAUGGUACUCCUUAAGCUAAC	13.91313962
plate	29	C03	hsa-miR-5696	MIMAT0022489	CUCAUUUAAGUAGUCUGAUGCC	15.03606448
plate	29	C04	hsa-miR-6757-5p	MIMAT0027414	UAGGGAUGGGAGGCCAGGAUGA	-5.548733788
plate	29	C05	hsa-miR-6801-5p	MIMAT0027502	UGGUCAGAGGCAGCAGGAAAUGA	-19.227706
plate	29	C06	hsa-miR-6881-3p	MIMAT0027663	AUCCUCUUUCGUCCUUCCCACU	-28.54280454
plate	29	C07	hsa-miR-642b-5p	MIMAT0022736	GGUUCCCUCUCCAAAUGUGUCU	-8.555065747
plate	29	C08	hsa-miR-6812-5p	MIMAT0027524	AUGGGGUGAGAUGGGGAGGAGCAGC	10.76765559
plate	29	C09	hsa-miR-548aq-5p	MIMAT0022263	GAAAGUAAUUGCUGUUUUUGCC	15.04415471
plate	29	C10	hsa-miR-6851-5p	MIMAT0027602	AGGAGGUGGUACUAGGGGCCAGC	12.26920353
plate	29	C11	hsa-miR-153-5p	MIMAT0026480	UCAUUUUUGUGAUGUUGCAGCU	16.76899318
plate	29	C12				
plate	29	D01				
plate	29	D02	hsa-miR-548at-5p	MIMAT0022277	AAAAGUUAUUGCGGUUUUGGCU	14.79982957
plate	29	D03	hsa-miR-597-5p	MIMAT0003265	UGUGUCACUCGAUGACCACUGU	-13.38331901
plate	29	D04	hsa-miR-3145-5p	MIMAT0019205	AACUCCAAACACUCAAAACUCA	-58.55758271
plate	29	D05	hsa-miR-3616-5p	MIMAT0017995	AUGAAGUGCACUCAUGAUAUGU	3.331110016
plate	29	D06	hsa-miR-3667-3p	MIMAT0018090	ACCUUCCUCUCCAUGGGUCUUU	-20.51728973
plate	29	D07	hsa-miR-3943	MIMAT0018359	UAGCCCCCAGGCUUCACUUGGCG	-61.55906053
plate	29	D08	hsa-miR-4436b-5p	MIMAT0019940	GUCCACUUCUGCCUGCCCUGCC	-25.00575306
plate	29	D09	hsa-miR-4537	MIMAT0019080	UGAGCCGAGCUGAGCUUAGCUG	-0.647668394
plate	29	D10	hsa-miR-4681	MIMAT0019766	AACGGGAAUGCAGGCUGUAUCU	-10.50319474
plate	29	D11	hsa-miR-4698	MIMAT0019793	UCAAAAUGUAGAGGAAGACCCCA	-67.85811882
plate	29	D12				
plate	29	E01				
plate	29	E02	hsa-miR-4770	MIMAT0019924	UGAGAUGACACUGUAGCU	5.54945292
plate	29	E03	hsa-miR-6515-3p	MIMAT0025487	UCUCUUCAUCUACCCCCCAG	-42.469338
plate	29	E04	hsa-miR-6728-5p	MIMAT0027357	UUGGGAUGGUAGGACCAGAGGGG	-35.27711757
plate	29	E05	hsa-miR-6756-5p	MIMAT0027412	AGGGUGGGGCUGGAGGUGGGGCU	6.240359135

plate	29	E06	hsa-miR-6770-5p	MIMAT0027440	UGAGAAGGCACAGCUUGCACGUGA	-18.58695926
plate	29	E07	hsa-miR-6783-5p	MIMAT0027466	UAGGGGAAAAGUCCUGAUCCGG	-34.94056373
plate	29	E08	hsa-miR-6807-5p	MIMAT0027514	GUGAGCCAGUGGAAUGGAGAGG	-23.73558589
plate	29	E09	hsa-miR-6818-5p	MIMAT0027536	UUGUGUGAGUACAGAGAGCAUC	-25.23713383
plate	29	E10	hsa-miR-6819-5p	MIMAT0027538	UUGGGGUGGAGGGCCAAGGAGC	-37.01651847
plate	29	E11	hsa-miR-6824-5p	MIMAT0027548	GUAGGGGAGGUUGGGCCAGGGA	-40.15067616
plate	29	E12				
plate	29	F01				
plate	29	F02	hsa-miR-6867-5p	MIMAT0027634	UGUGUGUGUAGAGGAAGAAGGGA	-6.047092369
plate	29	F03	hsa-miR-6893-5p	MIMAT0027686	CAGGCAGGUGUAGGGUGGAGC	5.889242862
plate	29	F04	hsa-miR-128-2-5p	MIMAT0031095	GGGGGCCGAUACACUGUACGAGA	17.64597484
plate	29	F05	hsa-miR-3681-5p	MIMAT0018108	UAGUGGAUGAUGCACUCUGUGC	5.30027363
plate	29	F06	hsa-miR-6726-5p	MIMAT0027353	CGGGAGCUGGGGUCUGCAGGU	7.154555882
plate	29	F07	hsa-miR-6823-5p	MIMAT0027546	UCAGGGUUGGUAGGGGUUGCU	10.85826624
plate	29	F08	hsa-miR-216a-3p	MIMAT0022844	UCACAGUGGUCUCUGGGAUUAU	-4.241351537
plate	29	F09	hsa-miR-1298-5p	MIMAT0005800	UUCAUUCGGCUGUCCAGAUGUA	-59.86011082
plate	29	F10	hsa-miR-4312	MIMAT0016864	GGCCUUGUUCCUGUCCCCA	-54.03675844
plate	29	F11	hsa-miR-6509-3p	MIMAT0025475	UUCCACUGCCACUACCUAAUUU	-43.72817883
plate	29	F12				
plate	29	G01				
plate	29	G02	hsa-miR-6889-5p	MIMAT0027678	UCGGGGAGUCUGGGGUCCGGAAU	-3.026197984
plate	29	G03	hsa-miR-3157-3p	MIMAT0019210	CUGCCCUAGUCUAGCUGAAGCU	4.850456469
plate	29	G04	hsa-miR-3677-5p	MIMAT0019221	CAGUGGCCAGAGCCCUGCAGUG	-22.35377347
plate	29	G05	hsa-miR-3925-5p	MIMAT0018200	AAGAGAACUGAAAGUGGAGCCU	-19.42348973
plate	29	G06	hsa-miR-3934-3p	MIMAT0022975	UGCUCAGGUUGCACAGCUGGGA	-30.20777526
plate	29	G07	hsa-miR-3945	MIMAT0018361	AGGGCAUAGGAGAGGGUUGAUAU	5.711257654
plate	29	G08	hsa-miR-4418	MIMAT0018930	CACUGCAGGACUCAGCAG	-20.91209329
plate	29	G09	hsa-miR-4434	MIMAT0018950	AGGAGAAGUAAAGUAGAA	-3.367605973
plate	29	G10	hsa-miR-4471	MIMAT0018998	UGGGAACUUAGUAGAGGUUUAA	-13.84608055
plate	29	G11	hsa-miR-4481	MIMAT0019015	GGAGUGGGCUGGUGGUU	6.330969786
plate	29	G12				
plate	29	H01				
plate	29	H02				
plate	29	H03				
plate	29	H04				
plate	29	H05				
plate	29	H06				
plate	29	H07				
plate	29	H08				
plate	29	H09				
plate	29	H10				
plate	29	H11				
plate	29	H12				
plate	30	A01				
plate	30	A02				
plate	30	A03				
plate	30	A04				
plate	30	A05				
plate	30	A06				

plate	30	A07				
plate	30	A08				
plate	30	A09				
plate	30	A10				
plate	30	A11				
plate	30	A12				
plate	30	B01				
plate	30	B02				
plate	30	B03				
plate	30	B04	hsa-miR-4522	MIMAT0019060	UGACUCUGCCUGUAGGCCGGU	2.605666363
plate	30	B05	hsa-miR-4632-5p	MIMAT0022977	GAGGGCAGCGUGGGUGUGGCGGA	8.069696908
plate	30	B06	hsa-miR-4646-3p	MIMAT0019708	AUUGUCCCUCUCCCUUCCCAG	-21.07563778
plate	30	B07	hsa-miR-4656	MIMAT0019723	UGGGCUGAGGGCAGGAGGCCUGU	10.41115011
plate	30	B08	hsa-miR-4677-5p	MIMAT0019760	UUGUUCUUUGGUCUUUCAGCCA	7.405645426
plate	30	B09	hsa-miR-4717-3p	MIMAT0019830	ACACAUGGGUGGCUGUGGCCU	14.78889991
plate	30	B10	hsa-miR-4749-3p	MIMAT0019886	CGCCCCUCCUGCCCCCACAG	-0.716510269
plate	30	B11	hsa-miR-4777-5p	MIMAT0019934	UUCUAGAUGAGAGAUAUAUAUA	21.25284612
plate	30	B12				
plate	30	C01				
plate	30	C02	hsa-miR-4799-5p	MIMAT0019976	AUCUAAAUGCAGCAUGCCAGUC	-16.54818851
plate	30	C03	hsa-miR-4804-3p	MIMAT0019985	UGCUUAACCUUGCCCUCGAAA	12.35148551
plate	30	C04	hsa-miR-6716-5p	MIMAT0025844	UGGGAAUGGGGGUAAGGGCC	1.139379276
plate	30	C05	hsa-miR-6732-3p	MIMAT0027366	UAACCCUGUCCUCUCCCUCCCAG	-16.70556488
plate	30	C06	hsa-miR-6822-5p	MIMAT0027544	CAGGGAACCAGUUGGGGCUU	4.54984021
plate	30	C07	hsa-miR-203b-3p	MIMAT0019814	UUGAACUGUUAAGAACCACUGGA	-15.27958149
plate	30	C08	hsa-miR-3166	MIMAT0015040	CGCAGACAAUGCCUACUGGCCUA	1.091398678
plate	30	C09	hsa-miR-4456	MIMAT0018978	CCUGGUGGCUUCCUUUU	3.388709729
plate	30	C10	hsa-miR-4670-3p	MIMAT0019751	UGAAGUUACAUCAUGGUCGCUU	6.701290242
plate	30	C11	hsa-miR-4679	MIMAT0019763	UCUGUGAUAGAGAUUCUUUGCU	7.035235207
plate	30	C12				
plate	30	D01				
plate	30	D02	hsa-miR-4778-3p	MIMAT0019937	UCUUCUUCCUUUGCAGAGUUGA	-1.637737758
plate	30	D03	hsa-miR-6738-3p	MIMAT0027378	CUUCUGCCUGCAUUCUACUCCCAG	-55.41631166
plate	30	D04	hsa-miR-7844-5p	MIMAT0030419	AAAACUAGGACUGUGUGGUGUA	-1.177124014
plate	30	D05	hsa-miR-3167	MIMAT0015042	AGGAUUUCAGAAAUACUGGUGU	6.494014057
plate	30	D06	hsa-miR-3622b-5p	MIMAT0018005	AGGCAUGGGAGGUCAGGUGA	4.592063137
plate	30	D07	hsa-miR-4289	MIMAT0016920	GCAUUGUGCAGGGCUAUCA	4.91065431
plate	30	D08	hsa-miR-4504	MIMAT0019040	UGUGACAAUAGAGAUGAACAUG	-0.394080648
plate	30	D09	hsa-miR-6752-5p	MIMAT0027404	GGGGGGUGUGGAGCCAGGGGGC	-1.474603724
plate	30	D10	hsa-miR-6769b-5p	MIMAT0027620	UGGUGGGUGGGGAGGAGAAGUGC	-5.203655831
plate	30	D11	hsa-miR-6796-5p	MIMAT0027492	UUGUGGGGUUGGAGAGCUGGCUG	5.179345661
plate	30	D12				
plate	30	E01				
plate	30	E02	hsa-miR-6816-3p	MIMAT0027533	GAAGGACCUGCACCUUCG	-5.585581394
plate	30	E03	hsa-miR-6835-5p	MIMAT0027570	AGGGGGUAGAAAGUGGCUGAAG	0.133705934
plate	30	E04	hsa-miR-7976	MIMAT0031179	UGCCCUGAGACUUUUGCUC	-18.01639483
plate	30	E05	hsa-miR-555	MIMAT0003219	AGGGUAAGCUGAACCUCUGAU	2.46748224
plate	30	E06	hsa-miR-2682-3p	MIMAT0013518	CGCCUCUUCAGCGCUGUCUUCC	3.822454338
plate	30	E07	hsa-miR-548ad	MIMAT0018946	GAAAACGACAAUGACUUUUGCA	8.751021405

plate	30	E08	hsa-miR-1537-5p	MIMAT0026765	AGCUGUAAUUAGUCAGUUUUCU	3.07203778
plate	30	E09	hsa-miR-1909-5p	MIMAT0007882	UGAGUGCCGGUGCCUGCCCUG	-25.98501261
plate	30	E10	hsa-miR-3691-3p	MIMAT0019224	ACCAAGUCUGCGUCAUCCUCUC	-11.45072974
plate	30	E11	hsa-miR-3919	MIMAT0018193	GCAGAGAACAAAGGACUCAGU	-6.076902722
plate	30	E12				
plate	30	F01				
plate	30	F02	hsa-miR-4428	MIMAT0018943	CAAGGAGACGGGAACAUGGAGC	-4.305459029
plate	30	F03	hsa-miR-4438	MIMAT0018956	CACAGGCUUAGAAAAGACAGU	-3.052205799
plate	30	F04	hsa-miR-4661-5p	MIMAT0019729	AACUAGCUCUGUGGAUCCUGAC	2.294752086
plate	30	F05	hsa-miR-4703-3p	MIMAT0019802	UGUAGUUGUAUUGUAUUGCCAC	3.672754871
plate	30	F06	hsa-miR-4713-3p	MIMAT0019821	UGGGAUCCAGACAGUGGGAGAA	10.2096316
plate	30	F07	hsa-miR-4729	MIMAT0019851	UCAUUUAUCUGUUGGGAAGCUA	9.100320161
plate	30	F08	hsa-miR-4797-3p	MIMAT0019973	UCUCAGUAAGUGGCACUCUGU	13.39746255
plate	30	F09	hsa-miR-5000-3p	MIMAT0021020	UCAGGACACUUCUGAACUUGGA	0.665330964
plate	30	F10	hsa-miR-5197-3p	MIMAT0021131	AAGAAGAGACUGAGUCAUCGAAU	-3.51090032
plate	30	F11	hsa-miR-6753-5p	MIMAT0027406	CACCAGGGCAGAGCAGGGCUGA	2.653646962
plate	30	F12				
plate	30	G01				
plate	30	G02	hsa-miR-6765-3p	MIMAT0027431	UCACCUGGCUGGCCCGCCCAG	-3.343927837
plate	30	G03	hsa-miR-6766-5p	MIMAT0027432	CGGGUGGGAGCAGAUCUUAUUGAG	-2.441892587
plate	30	G04	hsa-miR-6821-5p	MIMAT0027542	GUGCGUGGUGGCUCGAGGCGGGG	-0.910351887
plate	30	G05	hsa-miR-6864-5p	MIMAT0027628	UUGAAGGGACAAGUCAGAUAUGCC	-37.03014636
plate	30	G06	hsa-miR-6885-5p	MIMAT0027670	AGGGGGGCACUGCGCAAGCAAAGCC	10.46104993
plate	30	G07	hsa-miR-6890-5p	MIMAT0027680	CAUGGGGUAGGGCAGAGUAGG	12.16340156
plate	30	G08	hsa-miR-7111-3p	MIMAT0028120	AUCCUCUCUUCCCUCCUCCCAG	6.188857451
plate	30	G09	hsa-miR-7114-5p	MIMAT0028125	UCUGUGGAGUGGGGUGCCUGU	12.49350808
plate	30	G10	hsa-miR-323a-5p	MIMAT0004696	AGGUGGUCCGUGGCGCGUUCGC	5.542078985
plate	30	G11	hsa-miR-520f-5p	MIMAT0026609	CCUCUAAAGGGAAGCGCUUUCU	4.267714292
plate	30	G12				
plate	30	H01				
plate	30	H02				
plate	30	H03				
plate	30	H04				
plate	30	H05				
plate	30	H06				
plate	30	H07				
plate	30	H08				
plate	30	H09				
plate	30	H10				
plate	30	H11				
plate	30	H12				
plate	31	A01				
plate	31	A02				
plate	31	A03				
plate	31	A04				
plate	31	A05				
plate	31	A06				
plate	31	A07				
plate	31	A08				

plate	31	A09				
plate	31	A10				
plate	31	A11				
plate	31	A12				
plate	31	B01				
plate	31	B02				
plate	31	B03				
plate	31	B04	hsa-miR-3169	MIMAT0015044	UAGGACUGUGCUUGGCACAUAG	-10.57687695
plate	31	B05	hsa-miR-4680-5p	MIMAT0019764	AGAACUCUUGCAGUCUUAGAUGU	1.687874359
plate	31	B06	hsa-miR-5587-3p	MIMAT0022290	GCCCCGGGCAGUGUGAUCAUC	-38.61580232
plate	31	B07	hsa-miR-563	MIMAT0003227	AGGUUGACAUACGUUUCCC	3.859917301
plate	31	B08	hsa-miR-578	MIMAT0003243	CUUCUUGUGCUCUAGGAUUGU	0.442595323
plate	31	B09	hsa-miR-4257	MIMAT0016878	CCAGAGGUGGGGACUGAG	-9.325841619
plate	31	B10	hsa-miR-487b-5p	MIMAT0026614	GUGGUUAUCCCUGUCCUGUUCG	-25.05972159
plate	31	B11	hsa-miR-487a-5p	MIMAT0026559	GUGGUUAUCCCUGCUGUGUUCG	-3.204978558
plate	31	B12				
plate	31	C01				
plate	31	C02	hsa-miR-520g-5p	MIMAT0026611	UCUAGAGGAAGCACUUUCUGUUU	6.14900496
plate	31	C03	hsa-miR-1266-3p	MIMAT0026742	CCCUGUUCUAUGCCCUGAGGGA	-7.662271627
plate	31	C04	hsa-miR-3136-3p	MIMAT0019203	UGGCCCAACCUAUUCAGUUAGU	4.140057115
plate	31	C05	hsa-miR-3591-5p	MIMAT0019876	UUUAGUGUGAUAAUGGCGUUUGA	-10.55385176
plate	31	C06	hsa-miR-3680-3p	MIMAT0018107	UUUUGCAUGACCCUGGGAGUAGG	-48.8255554
plate	31	C07	hsa-miR-3685	MIMAT0018113	UUUCCUACCCUACCUGAAGACU	-43.63529378
plate	31	C08	hsa-miR-3907	MIMAT0018179	AGGUGCUCCAGGCUGGCUCACA	10.36837106
plate	31	C09	hsa-miR-3921	MIMAT0018196	UCUCUGAGUACCAUAUGCCUUGU	-56.00365845
plate	31	C10	hsa-miR-3922-5p	MIMAT0019227	UCAAGGCCAGAGGUCCCACAGCA	-16.2487488
plate	31	C11	hsa-miR-3927-3p	MIMAT0018202	CAGGUAGAUAUUUGAUAGGCAU	-15.52729285
plate	31	C12				
plate	31	D01				
plate	31	D02	hsa-miR-4288	MIMAT0016918	UUGUCUGCUGAGUUUCC	-1.762066639
plate	31	D03	hsa-miR-4433-5p	MIMAT0020956	CGUCCCACCCCCCACUCCUGU	-32.57936495
plate	31	D04	hsa-miR-4452	MIMAT0018974	UUGAAUUCUUGGCCUUAAGUGAU	-9.224147029
plate	31	D05	hsa-miR-4662b	MIMAT0019736	AAAGAUGGACAAUUGGCUAAAU	-4.358156834
plate	31	D06	hsa-miR-4676-3p	MIMAT0019759	CACUGUUUCACCACUGGCUCUU	-48.78142379
plate	31	D07	hsa-miR-4684-3p	MIMAT0019770	UGUUGCAAGUCGGUGGAGACGU	-11.81064339
plate	31	D08	hsa-miR-4712-3p	MIMAT0019819	AAUGAGAGACCUGUACUGUAU	0.772623049
plate	31	D09	hsa-miR-4714-3p	MIMAT0019823	CCAACCUAGGUGGUCAGAGUUG	-32.64844052
plate	31	D10	hsa-miR-4725-5p	MIMAT0019843	AGACCCUGCAGCCUUCCCACC	-11.36548972
plate	31	D11	hsa-miR-4730	MIMAT0019852	CUGGCGGAGCCCAUUCCAUGCCA	-8.166907045
plate	31	D12				
plate	31	E01				
plate	31	E02	hsa-miR-4751	MIMAT0019888	AGAGGACCCGUAGCUGCUAGAAGG	-48.91189987
plate	31	E03	hsa-miR-4757-3p	MIMAT0019902	CAUGACGUCACAGAGGCUUCGC	-23.40958296
plate	31	E04	hsa-miR-4779	MIMAT0019938	UAGGAGGGAAUAGUAAAAGCAG	-23.82403638
plate	31	E05	hsa-miR-4793-3p	MIMAT0019966	UCUGCACUGUGAGUUGGCUGGCU	-7.982705524
plate	31	E06	hsa-miR-4795-5p	MIMAT0019968	AGAAGUGGCUAAUAAUAUUGA	-0.111928008
plate	31	E07	hsa-miR-4999-3p	MIMAT0021018	UCACUACCUGACAAUACAGU	-30.09456318
plate	31	E08	hsa-miR-5094	MIMAT0021086	AAUCAGUGAAUGCCUUGAACCU	-29.37694475
plate	31	E09	hsa-miR-5186	MIMAT0021116	AGAGAUUGGUAGAAAUCAGGU	-1.950105692

plate	31	E10	hsa-miR-5192	MIMAT0021123	AGGAGAGUGGAUUCCAGGUGGU	-13.12307924
plate	31	E11	hsa-miR-5195-3p	MIMAT0021127	AUCCAGUUCUCUGAGGGGGCU	2.854483996
plate	31	E12				
plate	31	F01				
plate	31	F02	hsa-miR-5682	MIMAT0022470	GUAGCACCUUGCAGGAUAAGGU	-41.95253613
plate	31	F03	hsa-miR-5694	MIMAT0022487	CAGAUCAUGGGACUGUCUCAG	-49.09993892
plate	31	F04	hsa-miR-6081	MIMAT0023706	AGGAGCAGUGCCGGCCAAGGCGCC	-21.36225979
plate	31	F05	hsa-miR-6717-5p	MIMAT0025846	AGGCGAUGUGGGGAUGUAGAGA	3.556752297
plate	31	F06	hsa-miR-6719-3p	MIMAT0025850	UCUGACAUCAGUGAUUCUCCUG	-62.40466132
plate	31	F07	hsa-miR-6743-5p	MIMAT0027387	AAGGGGCAGGGACGGGUGGCCC	-1.992318541
plate	31	F08	hsa-miR-6768-5p	MIMAT0027436	CACACAGGAAAAGCGGGGCCCUG	-16.45597552
plate	31	F09	hsa-miR-6774-5p	MIMAT0027448	ACUUGGGCAGGAGGGACCCUGUAUG	-11.14675041
plate	31	F10	hsa-miR-6780b-3p	MIMAT0027573	UCCCUUGUCUCCUUUCCCUAG	-11.83174982
plate	31	F11	hsa-miR-6795-5p	MIMAT0027490	UGGGGGGACAGGAUGAGAGGCUGU	-9.544580926
plate	31	F12				
plate	31	G01				
plate	31	G02	hsa-miR-6797-3p	MIMAT0027495	UGCAUGACCCUUCCCUCCCCAC	-18.1425707
plate	31	G03	hsa-miR-6810-3p	MIMAT0027521	UCCCCUGCUCCCUUGUUCCCCAG	-33.64811753
plate	31	G04	hsa-miR-6815-5p	MIMAT0027530	UAGGUGGCGCCGGAGGAGUCAUU	-10.40994432
plate	31	G05	hsa-miR-6830-5p	MIMAT0027560	CCAAGGAAGGAGGCUGGACAUC	-37.11340866
plate	31	G06	hsa-miR-6836-3p	MIMAT0027575	AUGCCUCCCCCGGCCCCGCAG	-16.12978532
plate	31	G07	hsa-miR-6850-3p	MIMAT0027601	CCCGGCCGGAACGCCGCACU	0.404220006
plate	31	G08	hsa-miR-6857-3p	MIMAT0027615	UGACUGAGCUUCUCCCCACAG	-42.20773199
plate	31	G09	hsa-miR-6870-5p	MIMAT0027640	UGGGGGAGAUGGGGGUUGA	-4.189305439
plate	31	G10	hsa-miR-1251-5p	MIMAT0005903	ACUCUAGCUGCCAAAGGCGCU	-34.82623976
plate	31	G11	hsa-miR-3689a-3p	MIMAT0018118	CUGGGAGGUGUGAUAUCGUGGU	-19.74665895
plate	31	G12				
plate	31	H01				
plate	31	H02				
plate	31	H03				
plate	31	H04				
plate	31	H05				
plate	31	H06				
plate	31	H07				
plate	31	H08				
plate	31	H09				
plate	31	H10				
plate	31	H11				
plate	31	H12				
plate	32	A01				
plate	32	A02				
plate	32	A03				
plate	32	A04				
plate	32	A05				
plate	32	A06				
plate	32	A07				
plate	32	A08				
plate	32	A09				
plate	32	A10				

plate	32	A11				
plate	32	A12				
plate	32	B01				
plate	32	B02				
plate	32	B03				
plate	32	B04	hsa-miR-4467	MIMAT0018994	UGGCGGCGGUAGUUAUGGGCUU	6.143216834
plate	32	B05	hsa-miR-6745	MIMAT0027391	UGGGUGGAAGAAGGUCUGGUU	8.679036029
plate	32	B06	hsa-miR-6861-3p	MIMAT0027624	UGGACCUCUCCUCCCCAG	4.438283077
plate	32	B07	hsa-miR-548at-3p	MIMAT0022278	CAAAACCGCAGUAACUUUUGU	15.8206942
plate	32	B08	hsa-miR-548ao-3p	MIMAT0021030	AAAGACCGUGACUACUUUUGCA	10.86235837
plate	32	B09	hsa-miR-3193	MIMAT0015077	UCCUGCGUAGGAUCUGAGGAGU	-11.03740783
plate	32	B10	hsa-miR-3681-3p	MIMAT0018109	ACACAGUGCUUCAUCCACUACU	12.43060968
plate	32	B11	hsa-miR-4446-5p	MIMAT0019233	AUUUCCCUGCCAUUCCCUUGGC	-2.286133925
plate	32	B12				
plate	32	C01				
plate	32	C02	hsa-miR-4477a	MIMAT0019004	CUAUUAAGGACAUUUGUGAUUC	19.22516636
plate	32	C03	hsa-miR-4641	MIMAT0019701	UGCCCAUGCCAUACUUUUGCCUCA	-53.89814759
plate	32	C04	hsa-miR-5191	MIMAT0021122	AGGAUAGGAAGAAUGAAGUGCU	11.40908818
plate	32	C05	hsa-miR-5195-5p	MIMAT0021126	AACCCCUAAGGCAACUGGAUGG	-59.69456268
plate	32	C06	hsa-miR-6508-3p	MIMAT0025473	UGGGCCAUGCAUUUCUAGAACU	-50.42713267
plate	32	C07	hsa-miR-6780b-5p	MIMAT0027572	UGGGGAAGGCUUGGCAGGGAAGA	10.76704035
plate	32	C08	hsa-miR-6814-5p	MIMAT0027528	UCCCAAGGGUGAGAUGCUGCCA	-27.31341047
plate	32	C09	hsa-miR-6820-3p	MIMAT0027541	UGUGACUUCUCCCCUGCCACAG	-52.00077933
plate	32	C10	hsa-miR-410-5p	MIMAT0026558	AGGUUGUCUGUGAUGAGUUCG	13.0115101
plate	32	C11	hsa-miR-513b-3p	MIMAT0026749	AAAUGUCACCUUUUUGAGAGGA	11.86409688
plate	32	C12				
plate	32	D01				
plate	32	D02	hsa-miR-552-5p	MIMAT0026615	GUUUAACCUUUUGCCUGUUGG	15.90522151
plate	32	D03	hsa-miR-1321	MIMAT0005952	CAGGGAGGUGAAUGUGAU	11.74539896
plate	32	D04	hsa-miR-3189-5p	MIMAT0019217	UGCCCCAUCUGUGCCCUGGGUAGGA	9.520712188
plate	32	D05	hsa-miR-3191-5p	MIMAT0022732	CUCUCUGGCCGUCUACCUUCCA	-38.11312271
plate	32	D06	hsa-miR-3201	MIMAT0015086	GGGAUAUGAAGAAAAAU	8.218631977
plate	32	D07	hsa-miR-3689d	MIMAT0019008	GGGAGGUGUGAUCUCACACUCG	14.4143037
plate	32	D08	hsa-miR-3908	MIMAT0018182	GAGCAAUGUAGGUAGACUGUUU	15.0617469
plate	32	D09	hsa-miR-3935	MIMAT0018350	UGUAGAUACGAGCACCAGCCAC	-54.42509442
plate	32	D10	hsa-miR-4309	MIMAT0016859	CUGGAGUCUAGGAUUCCA	-38.02859541
plate	32	D11	hsa-miR-4474-3p	MIMAT0019001	UUGUGGCUGGUCAUGAGGCUAA	-53.57082909
plate	32	D12				
plate	32	E01				
plate	32	E02	hsa-miR-4642	MIMAT0019702	AUGGCAUCGUCCCCUGGUGGCU	-18.56933038
plate	32	E03	hsa-miR-4693-3p	MIMAT0019785	UGAGAGUGGAAUUCACAGUAUUU	9.536898267
plate	32	E04	hsa-miR-4699-3p	MIMAT0019795	AAUUUACUCUGCAAUCUUCUCC	-5.820094719
plate	32	E05	hsa-miR-4699-5p	MIMAT0019794	AGAAGAUUGCAGAGUAAGUUCC	6.483124513
plate	32	E06	hsa-miR-4733-5p	MIMAT0019857	AAUCCCAAUGCUAGACCCGGUG	-44.31598825
plate	32	E07	hsa-miR-4765	MIMAT0019916	UGAGUGAUUGAUAGCUAUGUUC	16.30447815
plate	32	E08	hsa-miR-4773	MIMAT0019928	CAGAACAGGAGCAUAGAAAGGC	-13.47970745
plate	32	E09	hsa-miR-5004-3p	MIMAT0021028	CUUGGAUUUUCCUGGGCCUCAG	-52.45398957
plate	32	E10	hsa-miR-5093	MIMAT0021085	AGGAAAUGAGGCUGGCUAGGAGC	-12.58587615
plate	32	E11	hsa-miR-6499-3p	MIMAT0025451	AGCAGUGUUUGUUUUGCCCACA	0.623763563

plate	32	E12				
plate	32	F01				
plate	32	F02	hsa-miR-6726-3p	MIMAT0027354	CUCGCCCUGUCUCCCGCUAG	-8.258797434
plate	32	F03	hsa-miR-6731-3p	MIMAT0027364	UCUAUUCCCCACUCUCCCCAG	-26.791859
plate	32	F04	hsa-miR-6732-5p	MIMAT0027365	UAGGGGGUGGCAGGCUGGCC	-8.149091781
plate	32	F05	hsa-miR-6750-5p	MIMAT0027400	CAGGGAACAGCUGGGUGAGCUGCU	-4.982015467
plate	32	F06	hsa-miR-6754-5p	MIMAT0027408	CCAGGGAGGCUGGUUUGGAGGA	-31.57574486
plate	32	F07	hsa-miR-6762-3p	MIMAT0027425	UGGCUGCUUCCCUUGGUCUCCAG	29.83963791
plate	32	F08	hsa-miR-6766-3p	MIMAT0027433	UGAUUGUCUUCCCCCACCCUCA	-35.34170613
plate	32	F09	hsa-miR-6782-5p	MIMAT0027464	UAGGGGUGGGGGAAUUCAGGGGUGU	-20.43972184
plate	32	F10	hsa-miR-6785-3p	MIMAT0027471	ACAUCGCCCCACCUUCCCCAG	-31.79335771
plate	32	F11	hsa-miR-6790-5p	MIMAT0027480	GUGAGUGUGGAUUUGGCGGGGUU	9.029734428
plate	32	F12				
plate	32	G01				
plate	32	G02	hsa-miR-6794-5p	MIMAT0027488	CAGGGGGACUGGGGGUGAGC	5.914813261
plate	32	G03	hsa-miR-6838-3p	MIMAT0027579	AAGUCCUGCUUCUGUUGCAG	-36.89736826
plate	32	G04	hsa-miR-6873-5p	MIMAT0027646	CAGAGGGAAUACAGAGGGCAAU	-42.81248127
plate	32	G05	hsa-miR-6895-5p	MIMAT0027690	CAGGGCCAGGCACAGAGUAAG	-23.13920029
plate	32	G06	hsa-miR-7106-5p	MIMAT0028109	UGGGAGGAGGGGAUCUUGGG	-1.858102032
plate	32	G07	hsa-miR-7151-3p	MIMAT0028213	CUACAGGCUGGAAUGGGCUCA	-30.38157185
plate	32	G08	hsa-miR-6886-5p	MIMAT0027672	CCCGCAGGUGAGAUGAGGGCU	3.551645585
plate	32	G09	hsa-miR-489-5p	MIMAT0026605	GGUCGUAUGUGUGACGCCAUUU	6.538876566
plate	32	G10	hsa-miR-548ah-5p	MIMAT0018972	AAAAGUGAUUGCAGUGUUUG	-1.09555782
plate	32	G11	hsa-miR-548ao-5p	MIMAT0021029	AGAAGUAACUACGGUUUUUGCA	7.675499071
plate	32	G12				
plate	32	H01				
plate	32	H02				
plate	32	H03				
plate	32	H04				
plate	32	H05				
plate	32	H06				
plate	32	H07				
plate	32	H08				
plate	32	H09				
plate	32	H10				
plate	32	H11				
plate	32	H12				
plate	33	A01				
plate	33	A02				
plate	33	A03				
plate	33	A04				
plate	33	A05				
plate	33	A06				
plate	33	A07				
plate	33	A08				
plate	33	A09				
plate	33	A10				
plate	33	A11				
plate	33	A12				

plate 33	B01				
plate 33	B02				
plate 33	B03				
plate 33	B04	hsa-miR-548m	MIMAT0005917	CAAAGGUAUUUGUGGUUUUUG	29.1789309
plate 33	B05	hsa-miR-581	MIMAT0003246	UCUUGUGUUCUCUAGAUCAGU	25.93273197
plate 33	B06	hsa-miR-598-5p	MIMAT0026620	GCGGUGAUCCCGAUGGUGUGAGC	31.88089151
plate 33	B07	hsa-miR-670-5p	MIMAT0010357	GUCCCUGAGUGUAUGUGGUG	11.20175814
plate 33	B08	hsa-miR-1227-5p	MIMAT0022941	GUGGGGCCAGGCGGUGG	31.78858253
plate 33	B09	hsa-miR-1292-3p	MIMAT0022948	UCGCGCCCCGGCUCCCGUUC	42.76950428
plate 33	B10	hsa-miR-1976	MIMAT0009451	CCUCCUGCCCUCCUUGCUGU	-77.02254412
plate 33	B11	hsa-miR-2117	MIMAT0011162	UGUUCUCUUUGCCAAGGACAG	18.67301576
plate 33	B12				
plate 33	C01				
plate 33	C02	hsa-miR-2276-3p	MIMAT0011775	UCUGCAAGUGUCAGAGGCGAGG	13.19986282
plate 33	C03	hsa-miR-3120-5p	MIMAT0019198	CCUGUCUGUGCCUGCUGUACA	38.38098179
plate 33	C04	hsa-miR-3124-3p	MIMAT0019200	ACUUUCCUCACUCCCGUGAAGU	40.82716961
plate 33	C05	hsa-miR-3127-3p	MIMAT0019201	UCCCCUUCUGCAGGCCUGCUGG	-120.6885352
plate 33	C06	hsa-miR-3190-5p	MIMAT0015073	UCUGGCCAGCUACGUCCCCA	-84.46495518
plate 33	C07	hsa-miR-3682-5p	MIMAT0019222	CUACUUCUACCUGUGUUAUCAU	35.59055842
plate 33	C08	hsa-miR-3692-3p	MIMAT0018122	GUUCCACACUGACACUGCAGAAGU	5.328599649
plate 33	C09	hsa-miR-3976	MIMAT0019361	UAUAGAGAGCAGGAAGAUUAAUGU	25.81349955
plate 33	C10	hsa-miR-4253	MIMAT0016882	AGGGCAUGUCCAGGGGGU	40.21562265
plate 33	C11	hsa-miR-4256	MIMAT0016877	AUCUGACCUGAUGAAGGU	14.57295881
plate 33	C12				
plate 33	D01				
plate 33	D02	hsa-miR-4266	MIMAT0016892	CUAGGAGGCCUUGGCC	-12.33126101
plate 33	D03	hsa-miR-4285	MIMAT0016913	GCGGCGAGUCCGACUCAU	45.19838417
plate 33	D04	hsa-miR-4299	MIMAT0016851	GCUGGUGACAUGAGAGGC	-27.12762036
plate 33	D05	hsa-miR-4313	MIMAT0016865	AGCCCCCUGGCCCCAAACCC	-13.34281352
plate 33	D06	hsa-miR-4432	MIMAT0018948	AAAGACUCUGCAAGAUGCCU	60.92937188
plate 33	D07	hsa-miR-4460	MIMAT0018982	AUAGUGGUUGUGAAUUUACCUU	35.22324562
plate 33	D08	hsa-miR-4482-5p	MIMAT0019016	AACCCAGUGGGCUAUGGAAAUG	34.43092693
plate 33	D09	hsa-miR-4482-3p	MIMAT0020958	UUUCUAUUUCUCAGUGGGGCUC	-19.80444173
plate 33	D10	hsa-miR-4494	MIMAT0019029	CCAGACUGUGGCUGACCAGAGG	-27.74301352
plate 33	D11	hsa-miR-4501	MIMAT0019037	UAUGUGACCUCGGAUGAAUCA	32.91552126
plate 33	D12				
plate 33	E01				
plate 33	E02	hsa-miR-4520a-5p	MIMAT0019235	CCUGCGUGUUUUCUGUCCAA	34.34823347
plate 33	E03	hsa-miR-4655-3p	MIMAT0019722	ACCCUCGUCAGGUCCCCGGGG	29.21931608
plate 33	E04	hsa-miR-4659b-5p	MIMAT0019733	UUGCCAUGUCUAAGAAGAA	27.81352732
plate 33	E05	hsa-miR-4659b-3p	MIMAT0019734	UUUCUUCUUAGACAUGGCAGCU	-115.5673102
plate 33	E06	hsa-miR-4663	MIMAT0019735	AGCUGAGCUCCAUGGACGUGCAGU	-95.12279551
plate 33	E07	hsa-miR-4664-3p	MIMAT0019738	CUUCCGGUCUGUGAGCCCCGUC	43.09450879
plate 33	E08	hsa-miR-4686	MIMAT0019773	UAUCUGCUGGGCUUUCUGGUGUU	29.51739714
plate 33	E09	hsa-miR-4697-3p	MIMAT0019792	UGUCAGUGACUCCUGCCCCUUGGU	-96.72089463
plate 33	E10	hsa-miR-4701-5p	MIMAT0019798	UUGGCCACCACACCUACCCCUU	-111.6191785
plate 33	E11	hsa-miR-4721	MIMAT0019835	UGAGGGCUCCAGGUGACGGUGG	13.97102738
plate 33	E12				
plate 33	F01				

plate	33	F02	hsa-miR-4736	MIMAT0019862	AGGCAGGUUAUCUGGGCUG	13.10755384
plate	33	F03	hsa-miR-4744	MIMAT0019875	UCUAAAGACUAGACUUCGCUAUG	23.31154172
plate	33	F04	hsa-miR-4753-3p	MIMAT0019891	UUCUCUUUCUUUAGCCUUGUGU	30.9212628
plate	33	F05	hsa-miR-4756-3p	MIMAT0019900	CCAGAGAUGGUUGCCUUCCUAU	-44.9855607
plate	33	F06	hsa-miR-4761-5p	MIMAT0019908	ACAAGGUGUGCAUGCCUGACC	-92.52083629
plate	33	F07	hsa-miR-4768-3p	MIMAT0019921	CCAGGAGAUCCAGAGAGAAU	22.57499303
plate	33	F08	hsa-miR-4796-5p	MIMAT0019970	UGUCUAUACUCUGUCACUUUAC	12.07292409
plate	33	F09	hsa-miR-4797-5p	MIMAT0019972	GACAGAGUGCCACUUACUGAA	49.04843764
plate	33	F10	hsa-miR-4802-3p	MIMAT0019982	UACAUGGAUGGAAACCUUCAAGC	17.28453493
plate	33	F11	hsa-miR-5000-5p	MIMAT0021019	CAGUUCAGAAGUGUUCCUGAGU	30.87895452
plate	33	F12				
plate	33	G01				
plate	33	G02	hsa-miR-5002-5p	MIMAT0021023	AAUUUGGUUUCUGAGGCACUUAGU	7.492091236
plate	33	G03	hsa-miR-5003-5p	MIMAT0021025	UCACAACAACCUUGCAGGGUAGA	-46.53173602
plate	33	G04	hsa-miR-5009-3p	MIMAT0021042	UCCUAAAUCUGAAAGUCCAAAA	26.5288941
plate	33	G05	hsa-miR-6074	MIMAT0023699	GAUAUUCAGAGGCUAGGUGG	9.926740435
plate	33	G06	hsa-miR-6505-3p	MIMAT0025467	UGACUUCUACCUCUUCCAAAG	24.5192508
plate	33	G07	hsa-miR-6506-5p	MIMAT0025468	ACUGGGAUGUCACUGAAUAUGGU	15.08258128
plate	33	G08	hsa-miR-6512-3p	MIMAT0025481	UUCCAGCCCUUCUAAUGGUAGG	-72.43786506
plate	33	G09	hsa-miR-6715a-3p	MIMAT0025841	CCAAACCAGUCGUGCCUGUGG	31.41742353
plate	33	G10	hsa-miR-6733-3p	MIMAT0027368	UCAGUGUCUGGAUUUCCUAG	35.38286322
plate	33	G11	hsa-miR-6735-3p	MIMAT0027372	AGGCCUGUGGCUCCUCCCUCAG	23.8807804
plate	33	G12				
plate	33	H01				
plate	33	H02				
plate	33	H03				
plate	33	H04				
plate	33	H05				
plate	33	H06				
plate	33	H07				
plate	33	H08				
plate	33	H09				
plate	33	H10				
plate	33	H11				
plate	33	H12				
plate	34	A01				
plate	34	A02				
plate	34	A03				
plate	34	A04				
plate	34	A05				
plate	34	A06				
plate	34	A07				
plate	34	A08				
plate	34	A09				
plate	34	A10				
plate	34	A11				
plate	34	A12				
plate	34	B01				
plate	34	B02				

plate	34	B03				
plate	34	B04	hsa-miR-6737-3p	MIMAT0027376	UCUGUGCUUCACCCCUACCCAG	-54.84627629
plate	34	B05	hsa-miR-6742-5p	MIMAT0027385	AGUGGGGUGGGACCCAGCUGUU	-34.87080784
plate	34	B06	hsa-miR-6746-3p	MIMAT0027393	CAGCCGCCGCCUGUCUCCACAG	2.776702022
plate	34	B07	hsa-miR-6762-5p	MIMAT0027424	CGGGGCCAUGGAGCAGCCUGUGU	5.534752813
plate	34	B08	hsa-miR-6786-3p	MIMAT0027473	UGACGCCCCUUCUGAUUCUGCCU	-39.50097595
plate	34	B09	hsa-miR-6789-3p	MIMAT0027479	CGGCGCCCGUGUCUCCUCCAG	-5.826178298
plate	34	B10	hsa-miR-6798-3p	MIMAT0027497	CUACCCCCCAUCCCCCUGUAG	-13.77393413
plate	34	B11	hsa-miR-6799-3p	MIMAT0027499	UGCCCUGCAUGGUGUCCCCACAG	-70.15427418
plate	34	B12				
plate	34	C01				
plate	34	C02	hsa-miR-6801-3p	MIMAT0027503	ACCCCUGCCACUCACUGGCC	-83.6857423
plate	34	C03	hsa-miR-6809-5p	MIMAT0027518	UGGCAAGGAAAGAAGAGGAUCA	-78.99029488
plate	34	C04	hsa-miR-6826-3p	MIMAT0027553	CUCCCCUCUCUUUCCUGUUCAG	-50.18113713
plate	34	C05	hsa-miR-6836-5p	MIMAT0027574	CGCAGGGCCCUGGCGCAGGCAU	-14.40341318
plate	34	C06	hsa-miR-6840-3p	MIMAT0027583	GCCCAGGACUUUGUGCGGGGUG	-29.39667153
plate	34	C07	hsa-miR-6853-3p	MIMAT0027607	UGUUCAUUGGAACCCUGCGCAG	-73.14779676
plate	34	C08	hsa-miR-6857-5p	MIMAT0027614	UUGGGGAUUGGGUCAGGCCAGU	-9.474825371
plate	34	C09	hsa-miR-6859-3p	MIMAT0027619	UGACCCCCAUGUCGCCUCUGUAG	-13.54312514
plate	34	C10	hsa-miR-6860	MIMAT0027622	ACUGGGCAGGGCUGUGGUGAGU	2.779033426
plate	34	C11	hsa-miR-6869-5p	MIMAT0027638	GUGAGUAGUGGCGCGCGGCGGC	-3.256971221
plate	34	C12				
plate	34	D01				
plate	34	D02	hsa-miR-6883-5p	MIMAT0027666	AGGGAGGGUGUGGUAUGGAUGU	20.53733679
plate	34	D03	hsa-miR-6887-3p	MIMAT0027675	UCCCCUCCACUUUCCUCCUAG	-12.63854044
plate	34	D04	hsa-miR-6891-3p	MIMAT0027683	CCCUCAUCUUCCCCUCCUUUC	-24.19764088
plate	34	D05	hsa-miR-6892-3p	MIMAT0027685	UCCCUCUCCCACCCCUUGCAG	-25.39831388
plate	34	D06	hsa-miR-7109-3p	MIMAT0028116	CAAGCCUCUCCUGCCCUUCCAG	-73.20608186
plate	34	D07	hsa-miR-7843-5p	MIMAT0030411	GAGGGCAGAGCCAGCUUCCUGA	-2.844312735
plate	34	D08	hsa-miR-8059	MIMAT0030986	GGGGAACUGUAGAUGAAAAGGC	7.940761618
plate	34	D09	hsa-miR-8060	MIMAT0030987	CCAUGAAGCAGUGGGUAGGAGGAC	-4.753732513
plate	34	D10	hsa-miR-8069	MIMAT0030996	GGAUGGUUGGGGGCGGUCGGCGU	2.862963966
plate	34	D11	hsa-miR-103b	MIMAT0007402	UCAUAGCCCUGUACAAUGCUGCU	-3.709263574
plate	34	D12				
plate	34	E01				
plate	34	E02	hsa-miR-367-5p	MIMAT0004686	ACUGUUGCUAAUAUGCAACUCU	29.83963827
plate	34	E03	hsa-miR-499b-3p	MIMAT0019898	AACAUCACUGCAAGUCUUAACA	26.86243551
plate	34	E04	hsa-miR-554	MIMAT0003217	GCUAGUCCUGACUCAGCCAGU	-47.58395321
plate	34	E05	hsa-miR-1203	MIMAT0005866	CCCGGAGCCAGGAUGCAGCUC	-32.03582073
plate	34	E06	hsa-miR-1265	MIMAT0005918	CAGGAUGUGGUCAAGUGUUGUU	11.6523566
plate	34	E07	hsa-miR-3184-5p	MIMAT0015064	UGAGGGGCCUCAGACCGAGCUUUU	-5.67696845
plate	34	E08	hsa-miR-6837-3p	MIMAT0027577	CCUUCACUGUGACUCUGCUGCAG	2.648474809
plate	34	E09	hsa-miR-372-5p	MIMAT0026484	CCUCAAAUGUGGAGCACUAUUCU	-6.532593674
plate	34	E10	hsa-miR-548ba	MIMAT0031175	AAAGGUAACUGUGAUUUUUGCU	11.33994848
plate	34	E11	hsa-miR-3646	MIMAT0018065	AAAAUGAAAUGAGCCCAGCCCA	-80.2632414
plate	34	E12				
plate	34	F01				
plate	34	F02	hsa-miR-3923	MIMAT0018198	AACUAGUAAUGUUGGAUUAGGG	20.9663151
plate	34	F03	hsa-miR-3938	MIMAT0018353	AAUUCCCUUGUAGAUAACCCGG	37.22319436

plate	34	F04	hsa-miR-4283	MIMAT0016914	UGGGGCUCAGCGAGUUU	-31.43198712
plate	34	F05	hsa-miR-4427	MIMAT0018942	UCUGAAUAGAGUCUGAAGAGU	-63.84782668
plate	34	F06	hsa-miR-4462	MIMAT0018986	UGACACGGAGGGUGGCUUGGGAA	3.737240421
plate	34	F07	hsa-miR-4661-3p	MIMAT0019730	CAGGAUCCACAGAGCUAGUCCA	-51.69421825
plate	34	F08	hsa-miR-4672	MIMAT0019754	UUACACAGCUGGACAGAGGCA	-10.03669371
plate	34	F09	hsa-miR-6734-3p	MIMAT0027370	CCCUUCCCUCACUCUUCUCUCAG	-80.26557281
plate	34	F10	hsa-miR-6754-3p	MIMAT0027409	UCUUCACCUGCCUCUGCCUGCA	-75.34397921
plate	34	F11	hsa-miR-6786-5p	MIMAT0027472	GCGGUGGGGCCGGAGGGGCGU	3.270959645
plate	34	F12				
plate	34	G01				
plate	34	G02	hsa-miR-6814-3p	MIMAT0027529	ACUCGCAUCCUUCCCUUGGCAG	-18.27587502
plate	34	G03	hsa-miR-6818-3p	MIMAT0027537	UUGUCUCUUGUUCCUCACACAG	26.7365397
plate	34	G04	hsa-miR-6846-5p	MIMAT0027592	UGGGGGCUGGAUGGGGUAGAGU	1.862791701
plate	34	G05	hsa-miR-6871-5p	MIMAT0027642	CAUGGGAGUUCGGGGUGGUUGC	2.438648459
plate	34	G06	hsa-miR-6883-3p	MIMAT0027667	UUCCCUAUCUCACUCUCCUCAG	-71.79791391
plate	34	G07	hsa-miR-6884-3p	MIMAT0027669	CCCAUCACCUUUCCGUCUCCCCU	10.40272412
plate	34	G08	hsa-miR-6892-5p	MIMAT0027684	GUAAGGGACCGGAGAGUAGGA	-8.815038073
plate	34	G09	hsa-miR-7848-3p	MIMAT0030423	CUACCCUCGGUCUGCUUACCACA	8.565577858
plate	34	G10	hsa-miR-8055	MIMAT0030982	CUUUGAGCACAUGAGCAGACGGA	-14.02339434
plate	34	G11	hsa-miR-215-3p	MIMAT0026476	UCUGUCAUUUCUUUAGGCCAAUA	-9.237022176
plate	34	G12				
plate	34	H01				
plate	34	H02				
plate	34	H03				
plate	34	H04				
plate	34	H05				
plate	34	H06				
plate	34	H07				
plate	34	H08				
plate	34	H09				
plate	34	H10				
plate	34	H11				
plate	34	H12				
